# Physiological and Proteomic Investigations to Study the Response of Tomato Graft Unions under Temperature Stress

**DOI:** 10.1371/journal.pone.0157439

**Published:** 2016-06-16

**Authors:** Sowbiya Muneer, Chung Ho Ko, Hao Wei, Yuze Chen, Byoung Ryong Jeong

**Affiliations:** 1 Division of Applied Life Science (BK21 Plus), Gyeongsang National University, Jinju, 660–701, Korea; 2 Institute of Agriculture and Life Science, Gyeongsang National University, Jinju, 660–701, Korea; 3 Research Institute of Life Science, Gyeongsang National University, Jinju, 660–701, Korea; Northeast Forestry University, CHINA

## Abstract

**Background:**

Grafting is an established practice for asexual propagation in horticultural and agricultural crops. The study on graft unions has become of interest for horticulturists using proteomic and genomic techniques to observe transfer of genetic material and signal transduction pathways from root to shoot and shoot to root. Another reason to study the graft unions was potentially to observe resistance against abiotic stresses. Using physiological and proteomic analyses, we investigated graft unions (rootstock and scions) of tomato genotypes exposed to standard-normal (23/23 and 25/18°C day/night) and high-low temperatures (30/15°C day/night).

**Results:**

Graft unions had varied responses to the diverse temperatures. High-low temperature, but not standard-normal temperature, induced the production of reactive oxygen species (ROS) in the form of H_2_O_2_ and O_2_^-1^ in rootstock and scions. However, the expression of many cell protection molecules was also induced, including antioxidant enzymes and their immunoblots, which also show an increase in their activities such as superoxide dismutase (SOD), catalase (CAT), and ascorbate peroxidase (APX). The graft interfaces thus actively defend against stress by modifying their physiological and proteomic responses to establish a new cellular homeostasis. As a result, many proteins for cellular defense were regulated in graft unions under diverse temperature, in addition to the regulation of photosynthetic proteins, ion binding/transport proteins, and protein synthesis. Moreover, biomass, hardness, and vascular transport activity were evaluated to investigate the basic connectivity between rootstock and scions.

**Conclusions:**

Our study provides physiological evidence of the grafted plants’ response to diverse temperature. Most notably, our study provides novel insight into the mechanisms used to adapt the diverse temperature in graft unions (rootstock/scion).

## Introduction

Temperature is a critical factor that influences plant growth and development; both yield and quality are reduced when the temperature is above or below optimal levels [1–3]. High-diverse temperature is increasingly prevalent because of global warming, and the harmful effects associated with high temperatures are reduction in the developmental phases of plant life cycle, and the processes like transpiration, photosynthesis, and respiration. The high temperature stress also affect cellular, physiological, biochemical and molecular response including protein denaturation and aggregation, cellular homeostasis disruption and fluidity increases in lipid membranes. An additional damaging effect of high temperatures is the inactivation of enzymes in chloroplasts, and mitochondria [4–6]. These injuries eventually lead to the production of harmful reactive oxygen species (ROS) [7]. To defend against cellular metabolism stress that is triggered by high temperature, plants respond by reprograming their transcriptome, proteome, and metabolome, which alters the levels of several transcripts, proteins, metabolites, and lipids. These changes cause the plant to balance its metabolic processes under high temperature. As, it is well known that temperature plays a central role for plant growth and development and this subject is a cross talk which has attracted a great response from agriculturalists and horticulturists for scrutinizing an optimal temperature for plant growth. The horticulturists widely use grafting technique for plant propagation and breeding to obtain better quality and other hybrid varities of fruits and vegetables however, greenhouse conditions always fluctuate with temperatures. The fluctuations in temperatures sometimes lead to high temperature which results in formation of ROS as described above. The temperature is more sensitive to grafted plants especially at initial stage at the time of grafting due to wounds. These reasons attracted the current study to investigate graft junctions to different temperatures and find out possible mechanisms to stress tolerance for grafted plants. Alternatively, a better approach for defense against biotic or abiotic stress during grafting is the eco-friendly technique of eliminating or reducing stresses such as salt stress [8] and soil-borne pathogens [9]. Previous studies have largely focused on physiological responses and protein synthesis or degradation in response to diverse temperature; however, proteomic analysis of graft unions will reveal novel consequences of diverse temperature (vascular connections) that have not been previously investigated.

Grafting is an asexual plant propagation technique that has been widely used in agricultural practice for thousands of years to aid crop cultivation. In general, woody plants are grafted to facilitate dwarfing, propagation, and firmness, while herbaceous plants are grafted to increase productivity and control the damage caused by biotic or abiotic stresses. Among the herbaceous plants, the highest consumed horticultural crops, such as tomatoes, cucumbers, sweet cherries, and melons, are typically grafted [10–13]. Grafting has been extensively used for decades in signaling pathways research and has led to the discovery of numerous plant processes, including the discovery of the flowering protein FLOWERING LOCUS T [14], the transcriptional changes related to graft interface-specific genes [15–18], and most recently, the rootstock/scion proteins involved in various plant processes such as the transport activity of vascular connections [19]. Rootstock and scion grafts also improve biotic or abiotic stress tolerance [8, 20–21].

Graft union development is an intricate process during which histological and physiological alterations occur, such as the regeneration of organs and even the exchange of genetic materials between rootstocks and scions [22]. Numerous imaging techniques and common histological methods have been predominately used for studying the physiological alterations that occur in rootstocks and scions during graft union development [12, 19, 23–25]. Numerous studies have characterized the morphological and physiological variations between graft unions [26–28], and a few recent studies investigated molecular modifications associated with graft unions [15–17, 29]. However, little is known about proteins or genes that are present in graft unions (specifically between rootstocks and scions) with the exception of our recent findings in watermelons grafted on bottle-gourds [19]. To investigate the proteins in graft unions, proteomics is an ideal tool that may reveal novel insight into the plant processes promoting rootstock/scions vascular connections, because proteomics provides a broad overview of baseline protein signals. Proteomics can also be used to investigate signal transduction pathways following photophosphorylation during plant photosynthesis [30].

The tomato (*Solanum lycopersicum* L.) is one of the most important crop species worldwide due to its high economic significance and its many promising genetic and agronomic features. The tomato has become a model plant for molecular studies that are aiming to improve fruit quality and resistance towards abiotic stresses [31–32]. However, many biotic and abiotic stresses affect the tomato, which cause severe annual yield losses worldwide. The present study was studied to reveal the mechanisms to temperature stress tolerance in graft unions of tomato cultivars. To follow our objective physiological and proteomic analysis were used and measurements included growth parameters such as biomass, hardness of graft unions, vascular transport activity, physiological measurements such H_2_O_2_ and O_2_^-1^ localizations, and molecular aspects such as western blots of stress-responsive proteins and proteomic analysis using 2-DE gel electrophoresis followed by mass spectrometry.

## Materials and Methods

### Plant materials, grafting procedure, and temperature treatments

Three genotypes that are widely used in Asian countries, particularly in Korea, were selected for this study: ‘Super Sunload’ and ‘Super Doterang’ as scions and ‘B-blocking’ as a rootstock. The two different genotypes for Scion ‘Super Sunload’ and ‘Super Doterang’ were used due to variations in their morphological changes under different temperatures as described to us by local growers of Korea. The other reason for selecting these cultivars were because of their popularity in Korea for fruit consumption. Whereas, ‘B-blocking’ were used as a rootstock due to strong firmness of root. The seeds obtained from Jeil Seed Company (Jeungpyeong-gun, Korea) were disinfected using 1% sodium hypochlorite (NaClO) followed by ten washing with 100 ml distilled water were germinated on square plug tray containing commercial Tosilee medium (Tosilee medium, Shinan Precision Co., Jinju, Korea) for four weeks in a greenhouse of Gyeongsang National University, Korea (Department of Horticulture). After germination and appearance of true leaves on all three tomato genotypes, plants were selected for grafting processes.

The grafting process was implemented by sharp dissecting blade four weeks post-germination as described in detail in our previous reports [19] (S1 Fig). The grafted plants were maintained at a relative humidity of 98±2% and a 16 h photoperiod with three different temperatures viz., 23/23°C (day/night), 25/18°C (day/night) (indicates standard-normal temperature) and 30/15°C (day/night) (indicates high-low temperature). The selection of these three particular temperatures were due to several reasons, 23/23°C and 25/18°C are standard temperatures used for plant growth and developments as reported in several studies and also mean of the both temperatures are slightly different from each other. Additionally, the vapour pressure deficit (VPD) was another reason to select these two temperatures. Whereas, 30/15°C was randomly selected as 30°C is considered very high for healing process during grafting and 15°C is very low for tomato plants. After two weeks the grafted plants grown under diverse temperatures, the firm rootstock and scions (vascular portions) were excised smoothly 2–3 cm (possibility of optimal genetic material at this point) around the grafted junction and immediately frozen in liquid N_2_ and stored at a deep-freezer (-80°C) for further analysis. For physiological measurements, the grafted plants were continuously observed from day 2^nd^ of grafting up to 2 weeks. The details about time point selection for physiological and proteomic analysis is given in detail in previous reports [19].

### Measurement of biomass and hardness of vascular connections

The biomass of rootstock and scions (around graft union, 2–3 cm) were constantly weighed on weighing balance from 2 days to 2 weeks as described in detail in our previous reports [19]

### Vascular staining (absorbable flower dye blue staining)

Vascular staining method was followed as described in our previous reports [19]. Samples were fixed in 0.1% absorbable flower dye blue for 20–30 min and were rinsed with water. The rootstock and scion samples were then cut into transverse and longitudinal section with the help of sharp surgical razor/blade and were observed under light microscope (Nikon Eclipse Ci-S/Ci-L, Japan)

### Histochemical localization of H_2_O_2_ and O_2_^-1^

H_2_O_2_ localization were analyzed by the method described by Liu et al. [33]. Briefly free-hand sections from rootstock and scions (2–3 cm graft junctions) were prepared transversely with a razor blade. The sections were soaked in 1% solution of 3,3’-diaminobenzidine (DAB) (Sigma-Aldrich) in Tris–HCl buffer (pH 6.5), vacuum-infiltrated for 5 min and then incubated at room temperature for 2 h in dark conditions. The sections were illuminated until the appearance of brown spots characteristic of the reaction of DAB (3’3-diaminobenzidine) with H_2_O_2_. The sections of rootstock and scions were bleached by immersing in boiling ethanol to visualize the brown spots and were photographed with a digital camera (Nikon Eclipse Ci-S/Ci-L, Japan) at a default resolution of 300 dpi.

*In situ* localization of O_2_^-1^ was detected by Romero-Puertas et al. [34], the transverse sections of rootstock and scions (2–3 cm graft junctions) were immersed in a 0.1% solution of nitro blue tetrazolium (NBT) (Sigma-Aldrich) in K-phosphate buffer (pH 6.4), containing 10 mM Na-azide, and were vacuum infiltrated for 5 min and illuminated until appearance of dark spots, characteristic of blue formazan precipitate. After bleaching in boiling ethanol, the rootstock/scions sections were photographed as described above for H_2_O_2_ localization.

### Antioxidant enzyme activities

For antioxidant enzyme activities about 0.5 g of rootstock/scion (2–3 cm graft junction) were homogenized in liquid nitrogen in precooled pestle and mortar. The grounded powder were extracted in 0.5 M extraction buffer (pH 7.5) by centrifugation at 13, 0000 rpm (Eppendorf centrifuge 5430R) for 20 min at 4°C. SOD activity in the supernatant was assayed for its ability to inhibit the photochemical reduction of nitro blue tetrazolium (NBT) by reading the absorbance at 560 nm as described by Dhindsa et al., [35]. One unit of enzyme activity was defined as the amount of enzyme required to inhibit 50% of the NBT photoreduction in comparison with tubes lacking the plant extract. Ascorbate peroxidase (APX) activity was estimated by measuring the absorbance of a supernatant at 290 nm (extinction coefficient of absorbance 2.8 mM^-1^ cm^-1^ for ascorbate) according to Nokano et al., [36]. One unit of enzyme was expressed as the amount necessary to decompose 1 μmole of ascorbate per min. Catalase (CAT) activity was estimated by measuring the absorbance in supernatant at 240 nm as a result of H_2_O_2_ degradation (extinction coefficient of 36 mM ^-1^ cm^-1^) as described by Aebi et al., [37]. One unit of enzyme determines the amount necessary to decompose 1 μm of H_2_O_2_ per min.

### Immunoblot analysis (Western blot)

For western blotting, the 2–3 cm graft junctions (rootstock/scions) were homogenized in extraction buffer containing 40 mM (w/v) Tris-HCl, pH 7.5, 2 mM (w/v) EDTA, 0.07% (w/v) β-mercaptoethanol, 2% (w/v) PVP and 1% (v/v) Triton X-100 as described in our previous studies [19]. The extract was centrifuged at 13,000 rpm (Eppendorf 5430R) for 10 min at 4°C. The supernatant was mixed with 6-X protein-dye containing 240 mM Tris-HCl (pH 6.8), 40% glycerol, 8% SDS, 0.04% bromophenol blue and 5% beta-mercaptoethanol. The samples containing 40 μg proteins were loaded on 12.5% polyacrylamide gel (Bio-Rad, Hercules, CA, USA). The protein concentration was determined by Bradford method using BSA (bovine serum albumin) as a standard curve. After electrophoresis, the gels were transferred to 0.45 μM nitrocellulose membrane (Sigma-Aldrich). After protein transfer, the blots were blocked with 5% nonfat dry skimmed milk or bovine serum albumin (Sigma-Aldrich). After washing with TBS, the blots were incubated with monoclonal primary antibodies 1:1000 dilution of anti-SOD (Cell Signaling #2770) for superoxide dismutase, 1:1000 dilution of anti-CAT (Cell signaling #12980) for catalase, and 1:1000 dilution of anti-APX/L (Cell signaling #AS08 368) for ascorbate peroxidase for 1:30 h. The blots were treated with 1:1000 dilution HRP-linked anti-rabbit 1gG (Cell Signaling #7074) for 1 h as a secondary antibody. Chemoluminance reactions were performed with super signal west pico chemiluminescent substrates (Cell Signaling SignalFire^TM^ ECL Reagent #6883) on X-ray films.

### Protein sample preparation for 2-DE

About 2–3 cm graft junction (rootstock/scion) from grafted tomato plants under diverse temperature were harvested and homogenized in liquid nitrogen in precooled pestle and mortar. The proteins were extracted in commercially available protein extraction buffer kit (Bio-Rad, Hercules, CA, USA) according to manufacturer’s instructions described in detail in our previous reports [19].

### Two-dimensional gel electrophoresis (2-DE) and silver staining

For isoelectric focusing (IEF), the Multiphor^TM^ II system (GE Healthcare) and IPG strip (pH 4–7, nonlinear, 11 cm, GE Healthcare) were used according to manufacturer’s instructions. The procedure for 2-DE and silver staining is described in detail in previous reports [19].

### Image and data analysis

In each treatment, three independent biological replicates were taken. Gels were taken under constant settings by a photo imager. The protein spots from all 2-DE gels were matched to have the same number across all gels. A master gel image containing matched spots across all gels was auto-generated. The missing spots from the 2-DE gels were resolved using an extensive analysis ‘landmark’ tool and respective spot volumes were normalized according to the total gel image density as recommended by the Progenesis SameSpots TotalLab (New Castle, UK). The gels from all the treatments were compared by creating three different replicate groups, and each replicate group contained the gel images corresponding to a specific treatment. In each group, an average quantity was determined for each spot, and pairwise quantitative and statistical analysis sets were generated by comparing the volume of a given spot across all treatments.

### Protein in gel digestion

The differential protein spots were excised manually from the 2D gels with the help of a clean razor blade and were chopped into small pieces. The description for in-gel digestion is given in detail in our previous reports [19].

### Protein identification using MALDI-TOF MS and MS/MS analysis

The digested peptide solution was spotted onto the MALDI-TOF MS target plate with a pipette. MALDI-MS analysis was performed with a Voyager DE-STR mass spectrometer (Applied Biosystems, Framingham, MA, USA). For full description please refer our previous reports [19].

### Protein functional classifications

The identified proteins were classified into different categories of biological processes in which they are involved according to gene ontology (http://www.geneontology.org/).

### Statistical analysis

For physiological parameters, a complete randomized design was utilized with four replicates. The Tukey’s studentized range test was employed to compare the means of separate replicates. Unless stated otherwise, the conclusions are predicted on differences between the means, with a significance level set at *P* < 0.05. For proteomic data, the coefficient of variation was computed for the matched spot quantities in each set of experiments with the replicate groups. All results were expressed as the Mean±SE. Differences between protein spots in all treatments were determined using two-way analysis of variance followed by a Student’s t-test with *p* < 0.05 as the limit of significance.

## Results and Discussion

### Physiological indices and vascular transport activity in rootstock and scions

**Fig 1:**
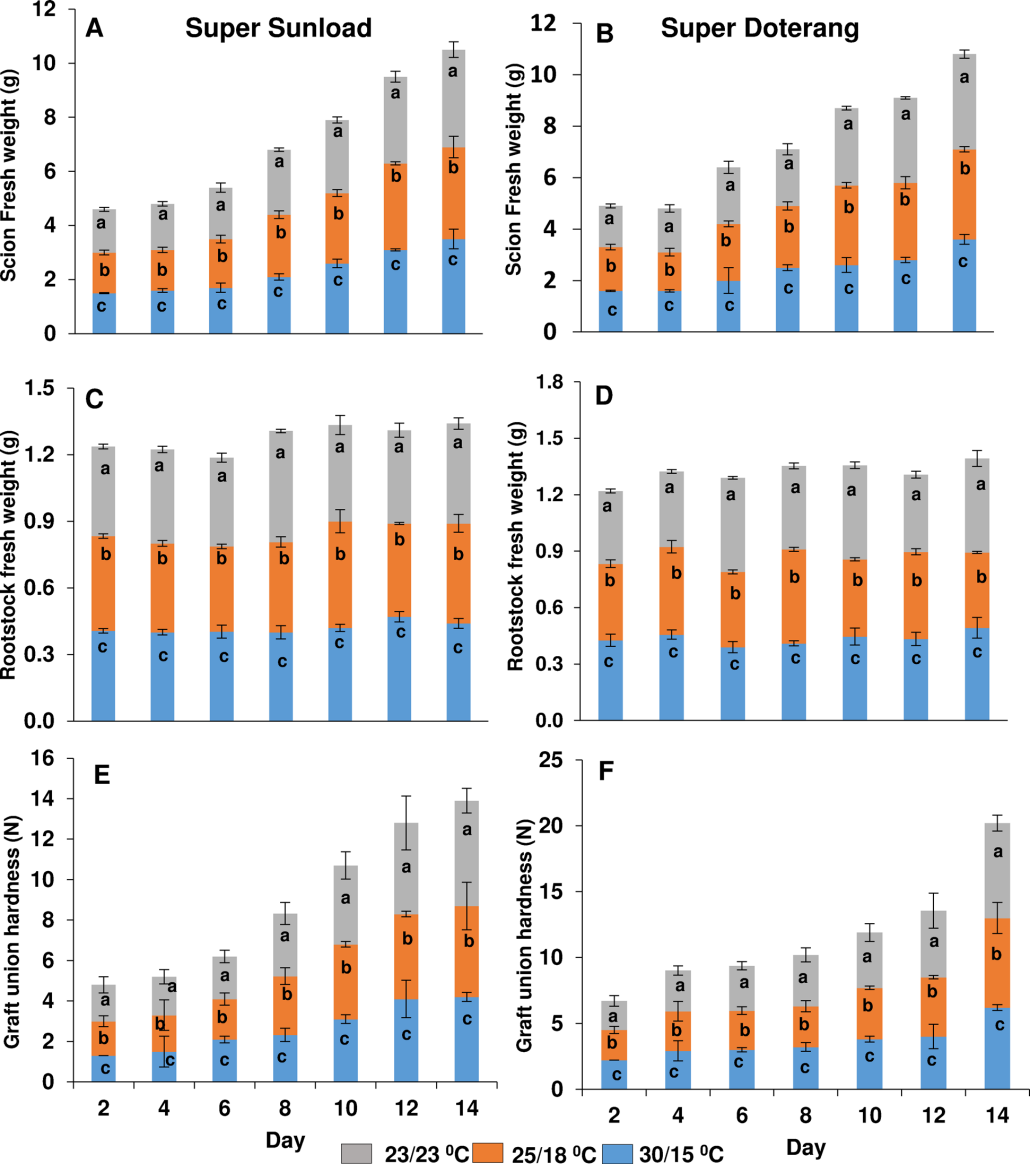
**(A-D) Biomass of rootstock/scions and (E-F) hardness of graft unions in ‘Super Sunload’ and ‘Super Doterang’ tomato scions grafted on ‘B-blocking’ as tomato rootstock grown under diverse day/night temperatures; normal-standard (23/23°C and 25/18**°**C) and high-low (30/15**°**C).** Line diagrams among different treatments represent Mean±SE of four biological replicates (n = 4).

Physiological responses such as the fresh/dry biomass and hardness of ‘Super Sunload’ and ‘Super Doterang’ scions grafted onto ‘B-blocking’ tomato rootstock and grown under diverse temperature (normal-standard and high-low) were evaluated to select the optimal time point for molecular and proteomic analyses. The fresh and dry biomass of the rootstock and scions of all tomato genotypes did not show any changes 48 h post grafting when grown under diverse temperature; however, a significant change was observed after 48 h (Fig 1) as the biomass of the rootstock and scions of all the tomato genotypes markedly decreased under high-low temperature (30/15°C) compared with normal conditions (23/23°C and 25/18°C). Similarly, the evaluation of the graft union pressure revealed a maximum hardness of the graft unions between ‘Super Sunload’ and ‘Super Doterang’ tomato scions grafted on ‘B-blocking’ tomato rootstock (Fig 1) two weeks after grafting under normal temperature conditions (23/23°C and 25/18°C). The high temperature causes loss of cell water content which ultimately lead to reduction in growth [38–39]. The other symptoms of temperature stress on plants are chlorosis and necrosis as also observed in our grafted plants (S1 Fig). Previous studies on non-grafted plants showed that standard day/night temperature regimes of either 23/23°C or 25/18°C, produced the maximum amount of plant biomass [6, 40–41]. Our results reinforce the hypothesis that standard temperature regimes are ideal for plant biomass, even in the case of grafted plants.

**Fig 2:**
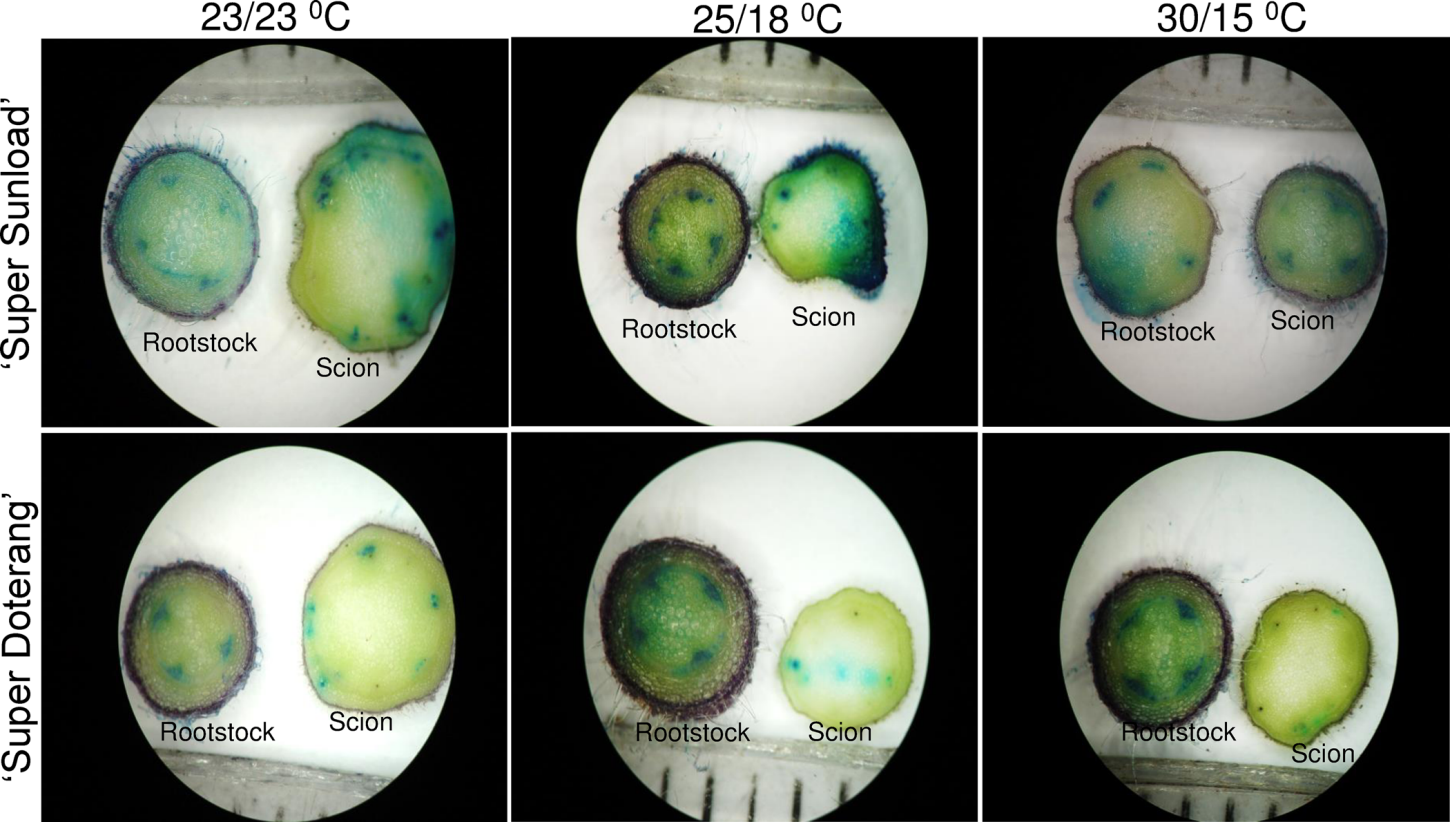
Horizontal sections of graft interfaces for vascular transport activity in ‘Super Sunload’ and ‘Super Doterang’ tomato scions grafted on ‘B-blocking’ as tomato rootstock grown under diverse day/night temperatures; normal-standard (23/23°C and 25/18°C) and high-low (30/15°C). The rootstocks were submerged in absorbable flower dye blue for 10–20 min and rootstock as well as scions were cut into transverse sections with razor blade. The cross sections were observed under light microscope.

In vascular transport we observed the passage of blue coloration from the rootstock to both scion genotypes ‘Super Sunload’ and ‘Super Doterang’, with particularly good passage observed using standard temperature regimes (23/23°C or 25/18°C) (Fig 2, S2 Fig). The transport of blue color was also observed under high-low temperature (30/15°C), but slight variations were observed in the scions of the tomato genotypes. Vascular transport activity was previously detected in many grafted plants, including sweet cherry [12], Arabidopsis [29], and watermelons [19]. Stained regions indicate appropriate and effective connections to vessels and the functional transport of nutrients and other important minerals between the rootstock and scion. The effect of temperature on vascular transport activity in grafted plants has not been previously demonstrated, and our results suggest that standard temperature may adequately facilitate transport of minerals and ions from rootstocks to scions.

### DAB and NBT mediated localization of H_2_O_2_ and O_2_^-1^ in rootstock and scions

**Fig 3:**
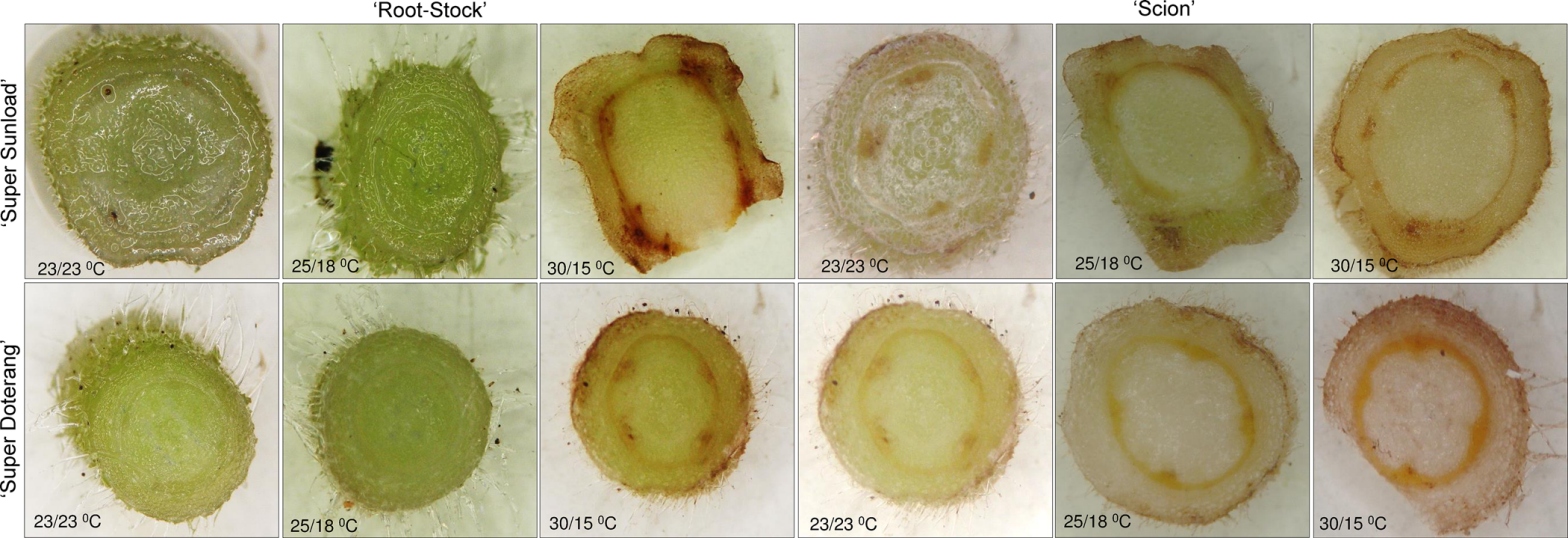
DAB-mediated localizations of H_2_O_2_ in ‘Super Sunload’ and ‘Super Doterang’ tomato scions grafted on ‘B-blocking’ as tomato rootstock grown under diverse day/night temperatures; normal-standard (23/23°C and 25/18°C) and high-low (30/15°C). The rootstocks and scions were cut into transverse sections with razor blade, stained with DAB and observed under light microscope.

**Fig 4:**
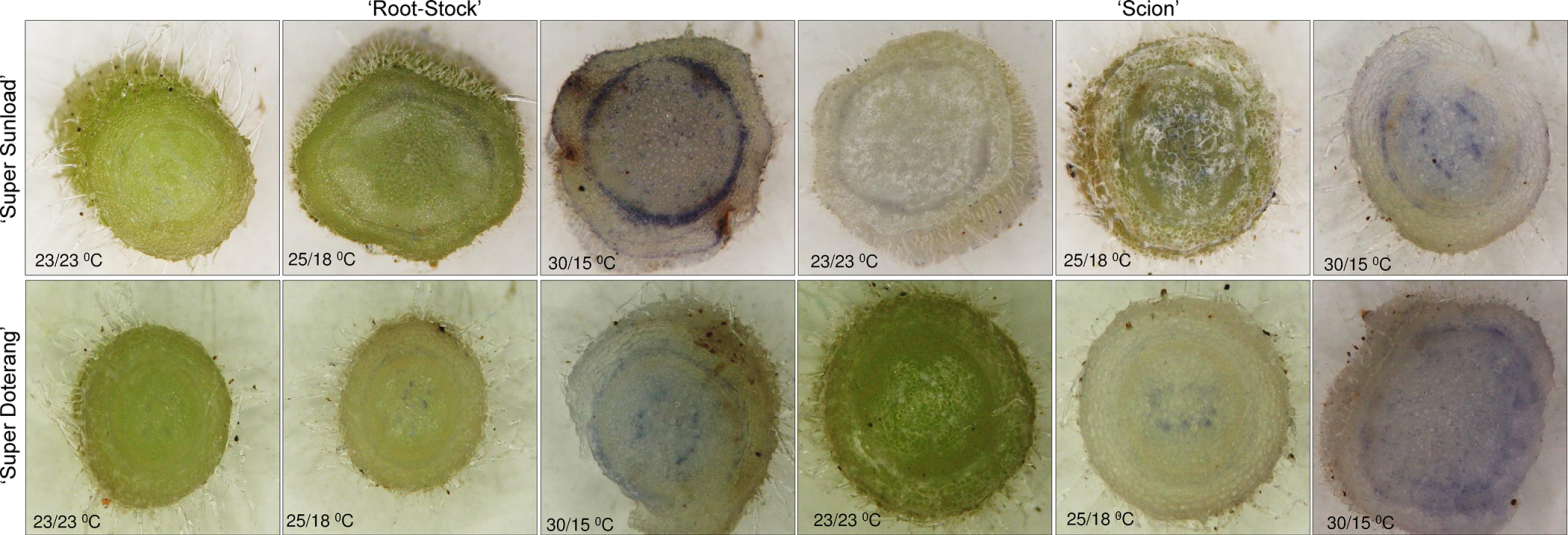
NBT-mediated localizations of O_2_^-1^ in ‘Super Sunload’ and ‘Super Doterang’ tomato scions grafted on ‘B-blocking’ as tomato rootstock grown under diverse day/night temperatures; normal-standard (23/23°C and 25/18°C) and high-low (30/15°C). The rootstocks and scions were cut into transverse sections with razor blade, stained with NBT and observed under light microscope.

The physiological results show that grafted tomato plants exhibited comprehensive fluctuations under high-low temperature (30/15°C) compared to normal-standard temperature (23/23 and 25/18°C). In previous experiments with non-grafted plants under high temperatures [2, 6], plants showed similar trends under instable temperature, and the first and foremost effect was the production of reactive oxygen species (ROS). We investigated ROS formation in rootstock and scions using DAB staining for H_2_O_2_ localization and NBT staining for O_2_^-1^ localization (Figs 3 and 4, respectively). We observed a significant reddish-brown coloration near epidermal and cortical regions of rootstock and scions grown under high-low temperature (30/15°C) (Fig 3). Less to no coloration was observed under normal-standard temperatures (23/23 or 25/15°C). A bluish color in the rootstock and scions that were grown under high-low temperature (30/15°C) showed that O_2_^-1^ was localized near epidermal and cortical regions (Fig 4), whereas no blue coloration was observed in the rootstock and scions that were grown under normal-standard temperatures (23/23 and 25/15°C).

Reactive oxygen species (ROS) accumulate when plants are exposed to abiotic or biotic stress, such as pathogen attack, high light, drought, heat, abnormal temperatures, or metal toxicities [42–44] in non-grafted plants. Oxidative stress in graft interfaces has been also reported under several abiotic stresses [45–46]. ROS involving H_2_O_2_ and O_2_^-1^ are vital signal molecules [47]. Under normal circumstances, ROS are sustained in homeostasis with an appropriate accumulation of antioxidant enzymes and other molecules located in various cell compartments of plants; however, when plants are under stress conditions, ROS are induced to levels that cause oxidative damage, as shown in previous studies [6, 48]. In this study, ROS were produced mostly in epidermal and cortical cells of the rootstock and scions of tomato plants under high-low temperature (30/15°C), whereas a sufficient equilibrium was maintained in normal-standard temperature conditions (23/23 or 25/15°C).

### Antioxidant enzyme activities in rootstock and scions and their western blot expression

**Fig 5:**
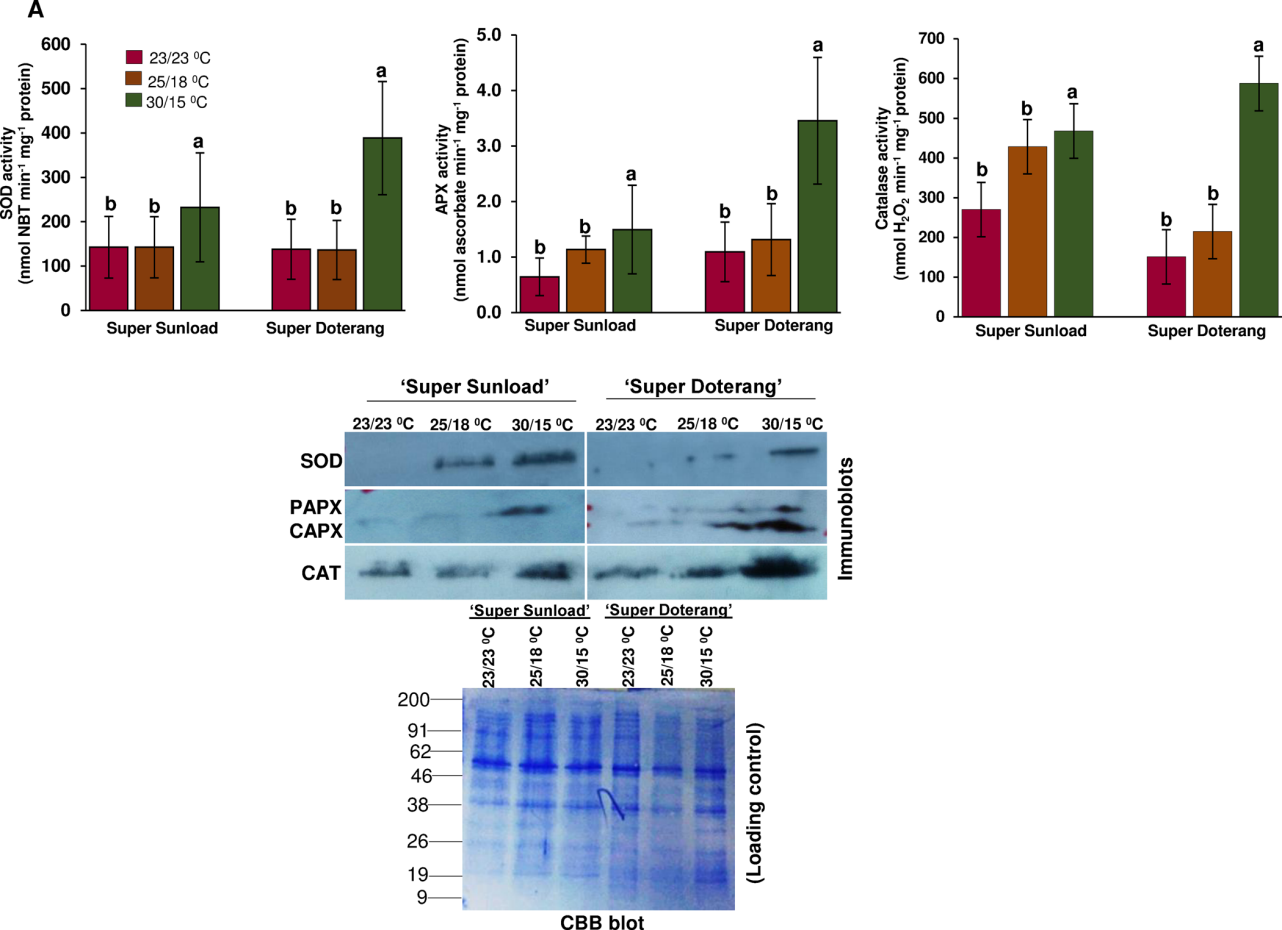
**(A) Antioxidant enzyme activities and (B) western blotting of SOD, APX, and CAT in ‘Super Sunload’ and ‘Super Doterang’ tomato scions grafted on ‘B-blocking’ as tomato rootstock grown under diverse day/night temperatures; normal-standard (23/23°C and 25/18°C) and high-low (30/15°C).** (Note: About 2–3 cm graft union (rootstock/scion) were cut near the junction and were used for the analysis). Bar diagrams represent Mean±SE of four biological replicates (n = 4). Significant differences among treatments are indicated by small letters with p ≤ 0.05 according Tukey’s test.

The H_2_O_2_ and O_2_^-1^ localization experiments in rootstock and scions confirmed formation of ROS under high-low temperature (30/15°C). To investigate the detoxification of oxidative damage, we evaluated three important enzyme activities and their immunoblots. Approximately 2–3 cm of graft junctions (rootstock/scions) from all treatments were selected for this analysis. The activities of antioxidant enzymes that play an essential role in ROS homeostasis in plants, such as superoxide dismutase (SOD), ascorbate peroxidase (APX), and catalase (CAT), were differently affected at the diverse temperature (Fig 5A). When grafted plants were exposed to high-low temperature (30/15°C), a significant increase was detected in all three antioxidant enzymes compared to plants exposed to normal-standard temperatures (23/23 and 25/15°C). Antioxidant enzyme activities (SOD, APX, and CAT) were further confirmed using immunoblot expression analysis (Fig 5B) [For uncropped images please see S3 Fig], which revealed a similar trend to the SOD, APX, and CAT expression patterns observed by Western blot.

Oxidative damage is a key cytotoxic consequence of ROS generation. The SOD enzyme plays an important role in the ascorbate-glutathione cycle by mediating the dismutation of superoxide anions to produce H_2_O_2_ [44]. Our results show a significant increase in relative enzyme activity and immunoblot expression of SOD under high-low temperatures (30/15°C) in the rootstock/scions of grafted tomatoes, which suggests an increase in SOD activity to quench ROS production. Similarly, the antioxidant enzymes APX and CAT play a significant role in ROS control. APX associates with glucose through the pentose phosphate pathway and NADPH to generate the reduced form of GSH from the oxidized disulfide form (GSSG). We observed that enzyme activity of APX and CAT increased markedly in high-low temperatures (30/15°C), which might be attributable to limitations in heme-catalyzed damage to the APX and CAT proteins, as shown by our immunoblots (Fig 5B). These data provide confirmation that grafting may aid plants in enduring abiotic stresses, particularly temperature or heat stress.

### Proteomic analysis in rootstock and scions

To repair the oxidative damage caused by abiotic stresses, including temperature, on cellular metabolism, plants respond by reprograming their transcriptome, proteome, and metabolome, which alters the expression of several transcripts, proteins, metabolites, and lipids. Our physiological data show that rootstock and scions exhibited remarkable changes under diverse temperatures. Previous studies mainly considered the physiological and transcriptome responses of grafted plants [15–16, 49–50]. With the exception of our recent finding on the proteome in graft unions of watermelons [19], there has been little research on protein expression in graft unions (vascular connections) that are under abiotic stress. To investigate proteins in graft unions under diverse temperatures, we used two-dimensional gel electrophoresis followed by mass spectrometry. This proteomic approach revealed novel insight about the graft unions of tomato genotypes for various plant processes, as subsequently discussed in detail.

**Fig 6:**
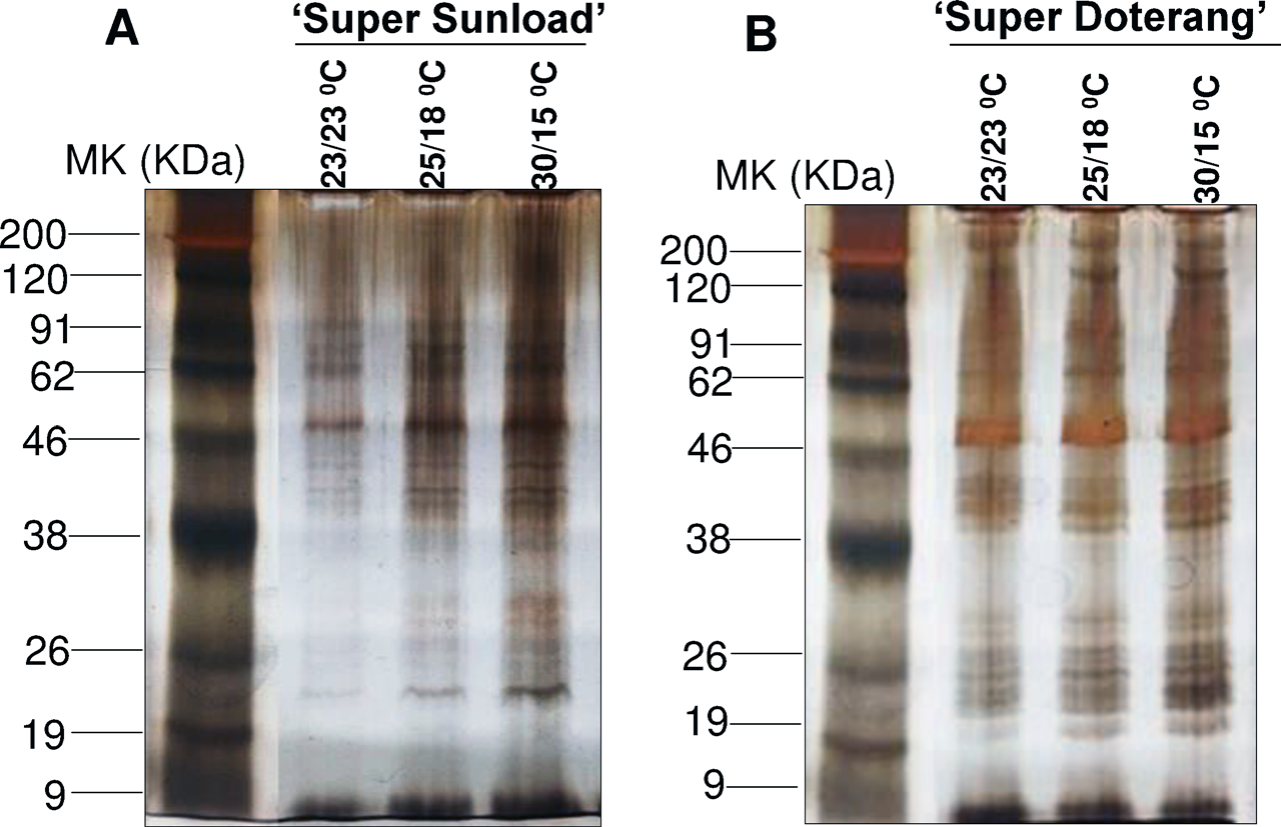
**First dimensional SDS-PAGE of in graft unions of (A) ‘Super Sunload’ (B) ‘Super Doterang’ tomato scions grafted on ‘B-blocking’ as tomato rootstock** grown under diverse day/night temperatures; normal-standard (23/23°C and 25/18°C) and high-low (30/15°C).

**Fig 7:**
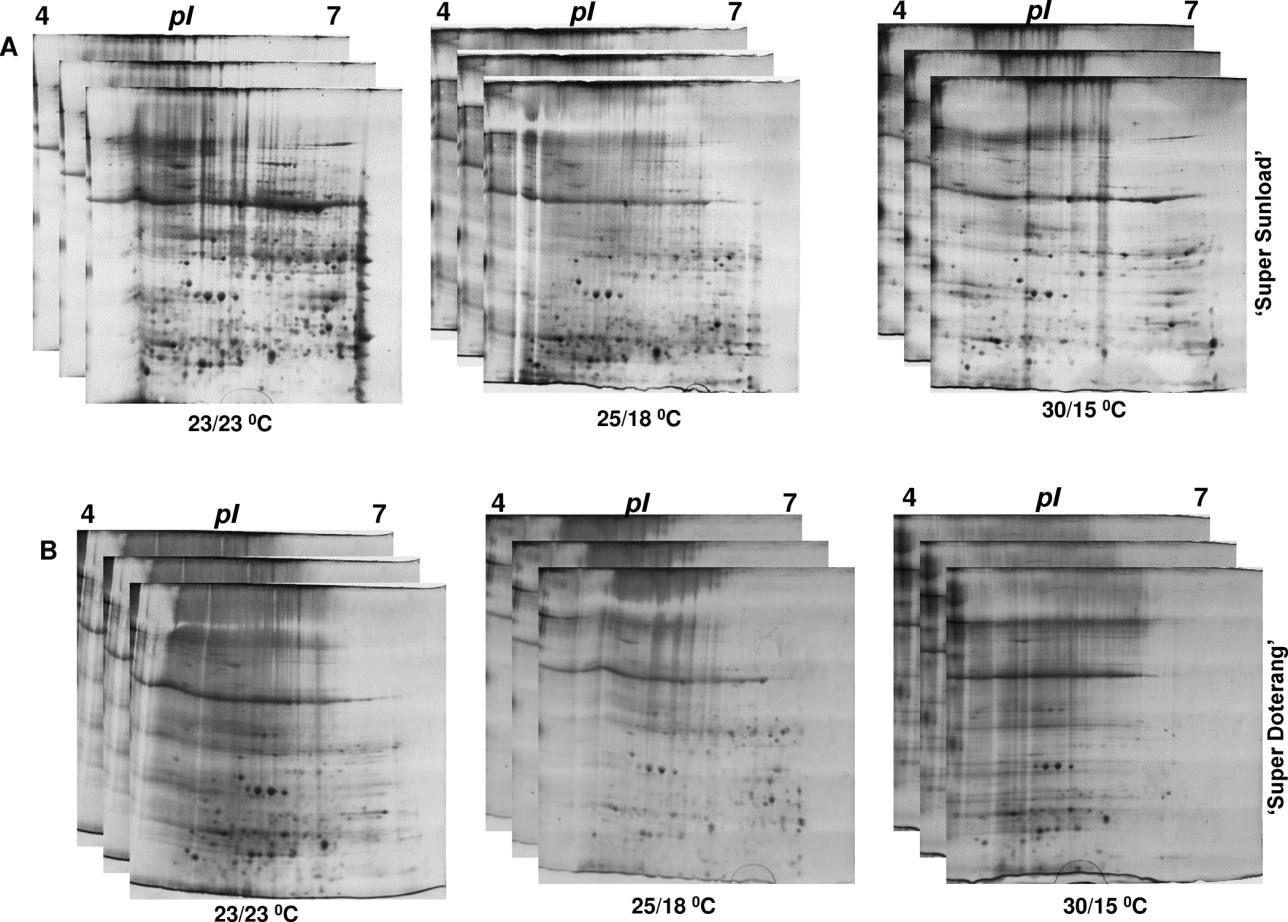
**Representative raw data of 2-DE gels with three technical replicates in graft unions (A) ‘Super Sunload’ (B) ‘Super Doterang’ tomato scions grafted on ‘B-blocking’ as tomato rootstock grown under diverse day/night temperatures; normal-standard (23/23°C and 25/18°C) and high-low (30/15°C).** About 70 μg of proteins from 2–3 cm graft unions were focused on 11 cm IPG strips (*pI* 4–7) for first dimension and separated on 12.5% (w/v) polyacrylamide gels (SDS-PAGE) for second dimension.

**Fig 8:**
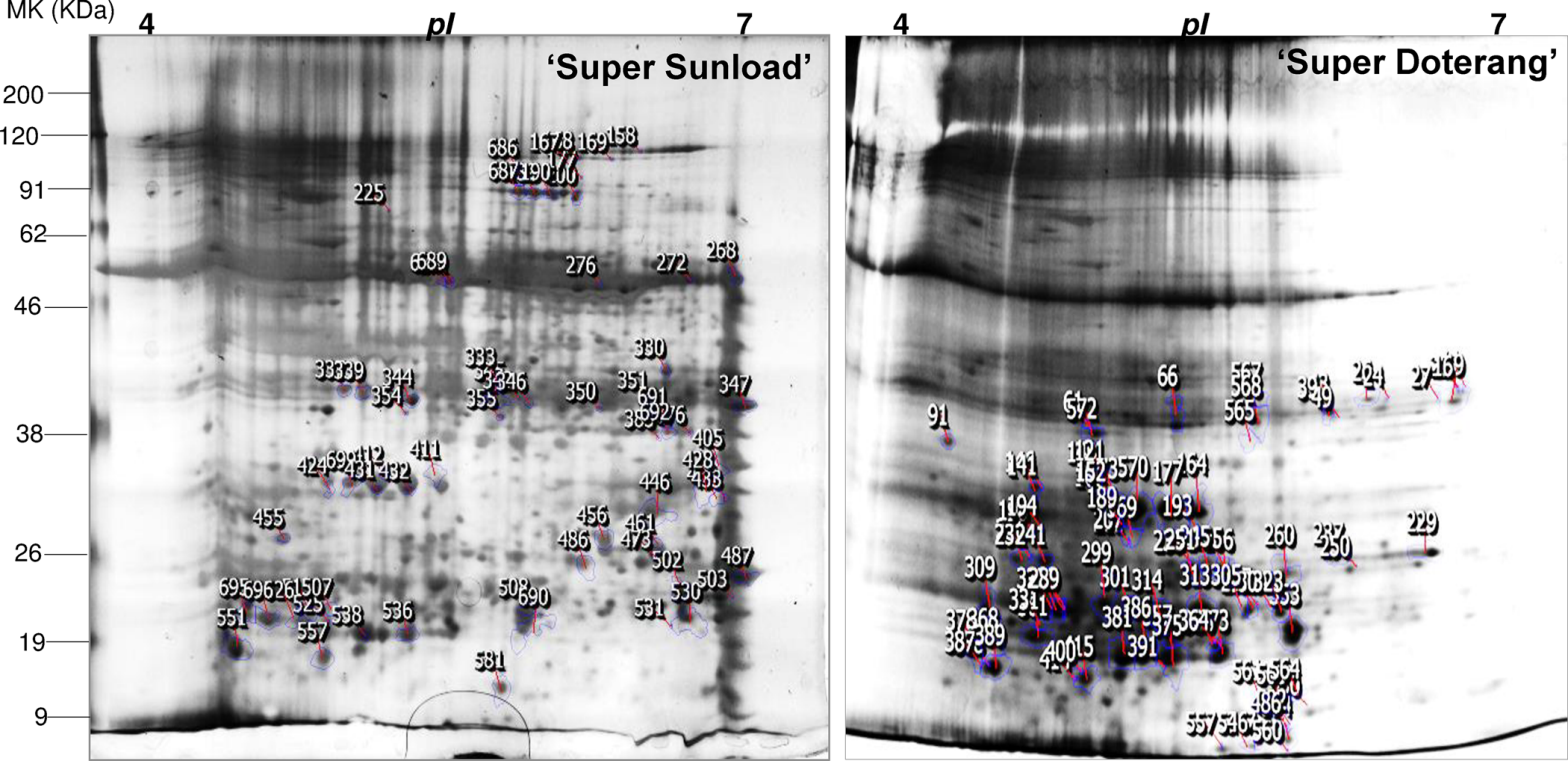
Higher level match set of protein spots detected by 2-DE. **The match set was created from three standard gels for each time point of 2-DE gels as shown in Fig 7.** The numbers on the gel indicate differentially expressed proteins. For descriptive quantification of differentially expressed proteins and easy spot picking please refer to S1 Table, S2 Table, and S5 Fig, which are actually numbered in Table 1 and Table 2.

**Fig 9:**
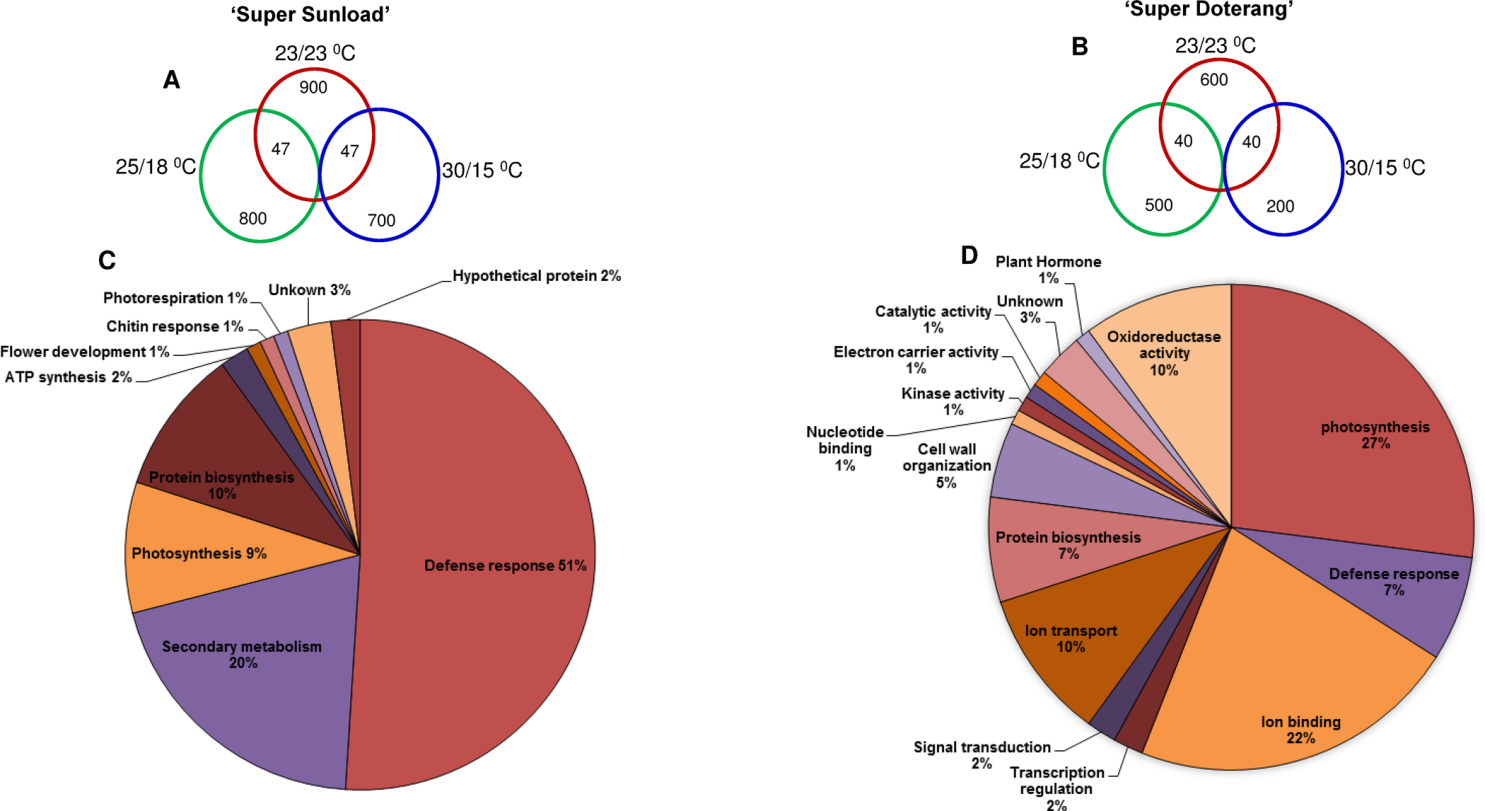
**(A-B) Comparative analysis and (C-D) Functional classification of identified proteins of graft unions in ‘Super Sunload’ and ‘Super Doterang’ tomato scions grafted on ‘B-blocking’ as tomato rootstock grown under diverse day/night temperatures; normal-standard (23/23°C and 25/18°C) and high-low (30/15°C).** The identified proteins (please see Table 1 and Table 2) were classified based on their putative biological functions.

The relative total protein profile was first evaluated in the first dimension using sodium dodecyl polyacrylamide gel electrophoresis (SDS-PAGE) (Fig 6). Comparative proteomic analysis of ‘Super Sunload’ and ‘Super Doterang’ scions grafted on ‘B-blocking’ rootstock was performed using IPG strips (*pI* 4–7) in 2–3 cm graft unions subjected to diverse temperatures (Fig 7, S4 Fig). The results show that 700–900 proteins were detected in the graft union of ‘Super Sunload’ and ‘B-blocking’ (Figs 8 and 9A, S5 Fig), and among the 700–900 proteins, 47 of them were differentially expressed between normal-standard (23/23 and 25/18°C) and high-low (30/15°C) temperatures. When the ‘Super Doterang’ scion was grafted onto ‘B-blocking’ rootstock, 200–600 proteins were detected on each of the 2-DE gels (Figs 8 and 9B), S5 Fig), and among the 200–600 protein, 40 were differentially expressed between normal-standard (23/23 and 25/18°C) and high-low (30/15°C) temperatures. Spot quantification and fold changes between differentially expressed proteins are presented in S1 Table and S2 Table.

### Identification and functional classification of proteins in rootstock and scions

The differentially expressed proteins from graft unions were excised from 2-DE gels and evaluated using MALDI-TOF MS (For spectra screen shots please see S1 File and S2 File) Approximately 90% of the 47 differentially expressed proteins from the graft union of ‘Super Sunload’ and ‘B-blocking’ and 40 from that of ‘Super Doterang’ and ‘B-blocking’ were successfully identified (Table 1 and Table 2, For MS-MS ion search please refer to S3 Table and S4 Table). Most of the proteins identified by mass spectrometry had functional annotations in the universal protein data bank, whereas only a few of the proteins were unknown or uncharacterized, with no functional annotations. The results indicate that most of the proteins identified under diverse temperatures had a significant relationship to the defense response, photosynthesis, ion transport or ion binding, and protein synthesis. These results provide an overview of the proteins involved in graft unions and unique insight for acclimatization under diverse temperatures. Using gene ontology analysis (www.geneontology.com), we classified 47 proteins identified from ‘Super Sunload’ grafts, and 40 proteins from ‘Super Doterang’ grafts, into different functional groups (Fig 9C and 9D).

**Table 1:** Proteins identified by MALDI-TOF MS in tomato ‘Super Sunload’ as the Scion and ‘B blocking’ as rootstock.

Spot No.	Protein Name	Plant species	gi number	Protein score	Biological function	Mr value	Calcu. *pI/* Exp. *pI*	Sequence coverage
1	-	*-*	-	-	-	-	-	-
2	Glycine rich RNA binding protein	*Brassica rapa*	gi|685366678	156	Defense response	16189	7.7/5.6	77
3	Chaperone protein ClpB3, chloroplastic isoform X1	*Eucalyptus grandis*	gi|702465984	150	Defense response	108781	6.1/5.7	47
4	BnaC08g04510D	*Brassica napus*	gi|674915372	149	Secondary Metabolism	17555	6.4/5.8	87
5	-	*-*	-	-	-	-	-	-
6	Kinesis-like protein NACK2	*Camelina sativa*	gi|727647144	51	Chloroplast accumulation movement	141541	4.8/5.1	44
7	Phosphoglycerate kinase, cytosolic like	*Phoenix dactylifera*	gi|672171816	62	Defense response	50730	8.2/7.0	58
8	Chaperone protein ClpD2, chlroplasticlike	*Setaria italica*	gi|514801217	60	Defense response	102421	8.5/4.5	48
9	F-box protein PP2-B10	*Solanum lycopersicum*	gi|723751136	152	Protein biosynthesis	31143	5.4/4.6	79
10	OJ1005_B10.30	*Oryza sativa*	gi|21902113	150	Unknown	18481	10.0/5.0	76
11	-	*-*	-	-	-	-	-	-
12	Hypothetical protein	*Capsella rubella*	gi|565483670	159	-	56015	9.4/4.5	63
13	Isopentenyl-diphosphate delta isomerase	*Medicago truncatula*	gi|357507779	153	Protein biosynthesis	32914	6.1/4.6	73
14	Elongation factor Ts, mitochondrial	*Auxenochlorella protothecoides*	gi|760438191	60	Protein biosynthesis	38838	6.4/5.0	71
15	-	*-*	-	-	-	-	-	-
16	Serine/threonine protein kinase TOUSLED	*Arabidopsis thaliana*	gi|18420352	50	ATP synthesis	78101	8.2/5.7	46
17	Malate dehydrogenase (NADP), chloroplastic	*Aegilops tauschii*	gi|475591288	152	Photosynthesis	41976	5.3/5.8	53
18	Hypothetical protein	*Populus trichocarpa*	gi|566187036	48	-	39813	4.5/6.0	59
19	Oxygen evolving enhancer protein 1–2, chloroplastic	*Camelina sativa*	gi|727467168	156	Photosynthesis	35401	6.0/6.8	64
20	Respiratory burst oxidase like protein C	*Glycine soja]*	gi|734403915	154	Defense response	15223	4.6/6.7	89
21	Guanine nucleotide binding protein subunit beta like protein	*Glycine soja*	gi|734377181	66	Defense response	24355	7.5/6.7	41
22	Salutaridine reductase like	*Vitis vinifera*	gi|731394250	50	Secondary Metabolism	22903	5.3/6.9	48
23	14-3-3-like protein	*Medicago truncatula*	gi|357465085	64	Defense response	23284	5.5/7.0	41
24	Predicted protein	*Hordeum vulgare*	gi|326526473	55	Unknown	69541	8.4/4.4	57
25	-	*-*	-	-	-	-	-	-
26	Hypothetical protein	*Eutrema salsugineum*	gi|567215911	62	-	26822	7.6/4.5	70
27	Petal death protein	*Eucalyptus grandis*	gi|702433120	65	Flower development	55227	6.9/4.4	57
28	Naringenin,2oxoglutarate 3 dioxygenase	*Gossypium arboreum*	gi|728819992	162	Protein biosynthesis	31082	7.0/4.5	84
29	Unnamed protein	*Coffea canephora*	gi|661887453	56	Unknown	122350	5.1/4.6	40
30	Specific tissue protein 1, putative	*Theobroma cacao*	gi|590685892	157	Defense response	39675	5.0/5.0	80
31	Hypothetical protein	*Zea mays*	gi|413947743	51	-	21539	4.9/5.1	82
32	Peroxisome biogenesis factor 10 isoform X2	*Nicotiana tomentosiformis*	gi|697125368	152	Photorespiration	35271	9.5/6.0	72
33	U box domain containing protein 27	*Setaria italica*	gi|514801386	152	Chitin response	45408	6.4/7.0	38
34	Predicted protein	*Micromonas sp*.	gi|255073969	58	Unknown	162180	6.0/6.8	37
35	Dehydrodolichyl diphosphate synthase	*Citrus sinensis*	gi|568825414	157	Protein biosynthesis	40876	5.9/5.9	72
36	Putative porphobilinogen deaminase	*Ostreococcus tauri*	gi|308803813	149	Secondary Metabolism	47732	9.5/6.0	60
37	UDPG pyrophosphorylase	*Gentiana triflora*	gi|685051837	61	Secondary Metabolism	52332	5.5/6.8	54
38	Serine/threonine protein kinase TIO	*Beta vulgaris*	gi|731321632	63	ATP synthesis	145082	8.4/6.9	16
39	Glycerate dehydrogenase isoform X1	*Phoenix dactylifera*	gi|672174015	63	Photosynthesis	46653	8.8/7.0	37
40	Dynamin related protein 5A	*Tarenaya hassleriana*	gi|729360263	152	Secondary Metabolism	90674	6.8/5.7	54
41	Dalanyldalanine carbox peptidase, putative	*Ricinus communis*	gi|255602672	152	Secondary Metabolism	29299	7.7/4.6	65
42	-	*-*	-	-	-	-	-	-
43	14-3-3-like protein	*Medicago truncatula*	gi|357465085	54	Defense response	23284	5.5/4.1	40

**Table 2:** Proteins identified by MALDI-TOF MS in tomato ‘Super Doterang’ as the Scion and ‘B blocking’ as rootstock.

Spot No.	Protein Name	Plant species	gi number	Protein score	Biological function	Mr value	Calcu. *pI/* Exp. *pI*	Sequence coverage
1	TRIGALACTOSYLDIACYLGLYCEROL 2, chloroplastic	*Prunus mume*	gi|645231498	52	Ion transport	40819	8.9/4.5	68
2	Histone-lysine N-methyltransferase MEDEA-like isoform X3	*Brassica rapa*	gi|685369870	93	Protein biosynthesis	67832	6.7/4.9	53
3	20 kDa chaperonin, chloroplastic	*Triticum urartu*	gi|473945885	100	Defense response/Photosynthesis	25891	8.8/5.2	67
4	B-box zinc finger protein	*Bambusa oldhamii*	gi|194245129	98	Transcription regulation	27083	4.9/5.5	59
5	histidine triad family protein	*Arabidopsis lyrata*	gi|297800468	60	Catalytic activity	16772	7.8/6.2	63
6	glyceraldehyde 3phosphate dehydrogenase GAPC1, chlroplasticlike	*Oryza brachyantha*	gi|573918618	77	photosynthesis	40309	8.2/6.3	69
7	toll/interleukin1 receptor like protein	*Eucalyptus grandis*	gi|702303259	144	Defense response	19220	5.1/7.0	82
8	ATPdependent Cip protease proteolytic subunit 2, partial	*Auxenochlorella protothecoides*	gi|760447615	146	Ion transport	24634	9.3/4.5	75
9	ATP synthase beta subunit, partial (chloroplast)	*Coleochaete soluta*	gi|19033065	152	Ion transport	43360	4.9/4.9	70
10	Hypothetical protein	*Gossypium raimondii*	gi|763772676	62	Unknown	49204	9.3/5.0	51
11	F-box/Kelchrepeat protein At3g23880like	*Fragaria vesca*	gi|470140284	152	Protein ubiquitination	45716	5.3/5.1	72
12	PREDICTED: flavonoid 3'monooxygenaselike	*Brachypodium distachyon*	gi|721620334	151	Ion binding	57732	7.8/5.2	67
13	Probable cinnamyl alcohol dehydrogenase 9	*Musa acuminata*	gi|695066375	154	Oxidoreductase activity	38475	6.2/5.3	79
14	Glycine rich RNAbinding protein	*Glycine soja*	gi|734418868	144	Transcription regulation	16409	5.8/4.5	89
15	Grx_S12 glutaredoxin subgroup I	*Zea mays*	gi|226505492	62	Electron carrier activity	18063	9.6/4.6	85
16	Kinesinlike calmodulinbinding protein	*Glycine soja*	gi|734422866	159	Oxidoreductase activity	79863	6.4/5.3	50
17	Root phototropism protein 3like	*Prunus mume*	gi|645222231	63	Signal transduction	66044	8.9/5.5	48
18	Unknown	*Lotus japonicus*	gi|388498152	52	-	26725	5.2/6.0	70
19	Helicase, Cterminal, Argonaute and Dicer protein, PAZ, Ribonuclease III, bacterial isoform 2	*Theobroma cacao*	gi|590720646	153	Ion binding	180152	5.9/6.2	30
20	NADPH:quinone reductase, partial	*Paeonia californica*	gi|723437961	147	Oxidoreductase activity	11425	5.3/6.8	90
21	Calciumbinding mitochondrial protein	*Brachypodium distachyon*	gi|357136915	156	Ion transport	66589	8.2/7.0	40
22	Clathrin adaptor complexes medium subunit family protein isoform 4	*Theobroma cacao*	gi|590675715	144	Protein biosynthesis	40101	9.1/4.5	51
23	Glycine rich cell wall structural protein 1.0like isoform X6	*Camelina sativa*	gi|727588178	150	Cell wall organization	28133	9.7/4.6	61
24	Glycine-rich cell wall structural protein-like isoform X2	*Nicotiana sylvestris*	gi|698478559	150	Cell wall organization	32597	10.2/4.7	57
25	GDPmannose pyrophosphorylase, partial	*Moringa oleifera*	gi|697651840	144	Defense response	37446	6.2/4.6	65
26	Hypothetical protein	*Erythranthe guttata*	gi|604331105	61	-	24832	6.8/5.0	81
27	Putative adenylate kinase	*Ostreococcus tauri*	gi|308803679	149	Kinase activity	28185	7.6/5.1	56
28	Phosphoenolpyruvate carboxylase, partial	*Alloteropsis cimicina*	gi|380691800	147	photosynthesis	96184	6.0/4.9	46
29	Photosystem I subunit VII	*Selaginella moellendorffii*	gi|255961344	147	photosynthesis	8792	8.6/5.1	89
30	AspartatetRNA ligase, cytoplasmic	*Tarenaya hassleriana*	gi|729465175	150	Ion transport	63293	6.1/5.2	58
31	PremRNAsplicing factor SLU7like isoform X1	*Phoenix dactylifera*	gi|672135838	156	Protein biosynthesis	62250	6.1/5.3	58
32	Cytochrome b5like heme/steroid binding domain	*Ostreococcus tauri*	gi|693500036	57	Ion binding	17920	5.0/5.2	89
33	DEAD-box ATPdependent RNA helicase 5	*Morus notabilis*	gi|703152401	149	Ion binding	34228	9.5/5.5	44
34	Pentatricopeptide (PPR) repeat protein	*Medicago truncatula*	gi|657389740	149	Nucleotide binding	78684	8.1/5.6	46
35	Hypothetical protein	*Eucalyptus grandis*	gi|629117084	43	-	36176	6.1/6.0	62
36	oxygen evolving enhancer protein 12	*Arabidopsis thaliana*	gi|15230324	153	Photosynthesis	34998	5.9/6.1	75
37	Arogenate dehydratase/prephenate dehydratase 1, chloroplastic isoform X1	*Vitis vinifera*	gi|731404317	150	Photosynthesis	43557	6.7/6.0	66
38	Auxin response factor 18like	*Vitis vinifera*	gi|225439992	152	Plant Hormone	77435	6.8/6.1	44
39	DNA ligase 1like	*Brachypodium distachyon*	gi|357140737	145	Ion binding	100742	9.3/5.4	28
40	glyoxylate/succinic semialdehyde reductase 2, chloroplastic isoform X1	*Eucalyptus grandis*	gi|702376573	147	photosynthesis	37774	8.8/5.5	67

### Proteins related to defense response

Stress responsive proteins play an important role in detoxification of numerous abiotic stresses [51–53]. The proteins identified in this study were identified as having defense/stress-response functions, and the results are summarized in Table 1 and Table 2. Under high-low temperature conditions, the proteins identified as defense/stress-response proteins triggered differential expressions in 2-DE gels, which suggests that the rootstocks might cause some stress on their scions, and vice versa. Stress-responsive proteins have also been previously observed in grafted rubber trees [51]. Additionally, several genes related to stress-response have been observed in grafted grapevine [15–16]. Our results also show putative up-regulation of proteins related to stress tolerance under high-low temperature, which was confirmed by immunoblot analysis and analysis of the activities of critical stress responsive proteins (Fig 5). Proteins related to defense/stress-response are hypothesized to play a role in healing during grafting [9, 16, 19]. Thus, our identification of stress/defense responsive proteins strongly implicate a robust antioxidant mechanism in graft unions under diverse temperature. Moreover, stress/defense responsive proteins were detected that have not been previously studied in graft unions.

### Proteins related to photosynthesis

Photosynthesis is an essential plant process and is sensitive to many environmental stresses including temperature and heat stress. In a proteomic study of graft unions, we observed that many proteins involved in the C_3_ or C_4_ (photosynthesis) cycle were down-regulated in response to diverse temperatures (Table 1 and Table 2). The differential expression of photosynthesis proteins suggests that cell communication related to photosynthetic activity is increased in graft unions. Although several photosynthetic proteins that respond to temperature/heat stress have been detected in non-grafted plants [7, 54–55], only a few genes related to photosynthesis have been observed in grafted apple and rubber trees [51]. The differential expression of photosynthetic proteins suggests a significant increase in diverse temperature in graft unions which may strongly interrupt the regulatory network of the photosynthetic system in the aerial parts of scions. The identification of photosynthetic proteins provides two important confirmations: the occurrence of anomalies in temperature for proper photosynthesis and the novel detection of photosynthetic proteins in graft unions.

### Proteins related to ion binding/ion transport

Proteins related to transport or binding of ions play a vital role in the vascular tissues of plants [19]. The abundant number of ion transport/binding proteins has been well documented in the vascular cambium of plants [56–57]. The proteomic analysis of graft unions in tomatoes revealed an increased number of ion binding/transport proteins (Table 1 and Table 2), which suggests appropriate communication between the rootstock and scions. Our 2-DE gels of tomato graft unions showed down-regulation of ion transport/binding under diverse temperature. This indicates that oxidative damage can disrupt the transport of minerals and ions. However, graft unions may be able to tolerate stress conditions to a certain point for optimal transport activity by increasing stress-responsive proteins, as shown by our immunoblot analysis and our analysis of the enzyme activities of stress-responsive proteins (Fig 5). The tolerance level for transport activity can also be observed in our vascular transport activity experiments (Fig 2), where the passage of blue dye (flower staining dye used to analyze transport activity) was at optimal levels, even under diverse temperature.

### Proteins related to protein synthesis

Protein synthesis in plants plays important physiological roles in the response to unfavorable conditions. The expression of genes that play a crucial role in the synthesis of proteins has been previously observed in many plants under diverse temperature [58–61]. Our proteome data (Table 1 and Table 2) of graft unions also identified several differentially expressed proteins that are related to protein synthesis. The presence of proteins related to protein synthesis in graft unions indicates the enhancement of translational processes, and may be a function of ribosomal activity for appropriate protein synthesis under diverse temperature. Our results also identified proteins related to protein synthesis that had not been previously observed in graft unions, with the exception of our previous study in grafted watermelon [19].

## Concluding Remarks

This study reports a systematic physiological and proteomic analysis of tomato plants grown under diverse temperatures using ‘Super Sunload’ and ‘Super Doterang’ as scions and ‘B-blocking’ as a rootstock. A total of 87 proteins were identified that responded to diverse temperatures, and these are involved in a wide range of cellular processes including defense/stress response, ion binding/transport, photosynthesis, and protein synthesis. In addition, a physiological analysis provided useful information for understanding the important role of temperature on graft unions. Our study provides unique insight on graft union (rootstock/scion) adaptation under diverse temperatures.

## Supporting Information

S1 Fig **Representative images of grafted tomato plants grown under diverse temperatures (day/night; 23/23** ^**0**^**C, 25/18** ^**0**^**C, and 30/15** ^**0**^**C) under greenhouse conditions** (A) ‘B-blocking’ used as rootstock and ‘Super Sunload’ as scion (B) ‘B-blocking’ used as rootstock and ‘Super Doterang’ as scion.
(JPG)

S2 Fig Longitudinal sections of graft unions analyzing vascular transport activity of ‘Super Sunload’ and ‘Super Doterang’ tomato scions grafted on ‘B-blocking’ as tomato rootstock grown under diverse day/night temperatures; normal-standard (23/23°C and 25/18°C) and high-low (30/15°C). The rootstocks were submerged in absorbable flower dye blue for 10–20 min and rootstock as well as scions were cut into transverse sections with razor blade. The cross sections were observed under light microscope.
(JPG)

S3 Fig Uncropped images of western blots of SOD, APX, and CAT in ‘Super Sunload’ and ‘Super Doterang’ tomato scions grafted on ‘B-blocking’ as tomato rootstock grown under diverse day/night temperatures; normal-standard (23/23°C and 25/18°C) and high-low (30/15°C). (Note: About 2–3 cm graft union (rootstock/scion) were cut near the junction and were used for the analysis).
(JPG)

S4 Fig **Representative raw data of 2-DE gels in graft unions (A) ‘Super Sunload’ (B) ‘Super Doterang’ tomato scions grafted on ‘B-blocking’ as tomato rootstock grown under diverse day/night temperatures; normal-standard (23/23°C and 25/18°C) and high-low (30/15°C).** About 70 μg of proteins from 2–3 cm graft unions were focused on 11 cm IPG strips (*pI* 4–7) for first dimension and separated on 12.5% (w/v) polyacrylamide gels (SDS-PAGE) for second dimension.
(JPG)

S5 Fig Higher level match set of protein spots detected by 2-DE. **The match set was created from three standard gels for each time point of 2-DE gels as shown in Fig 8.** The numbers on the gel indicate differentially expressed proteins. For descriptive quantification of differentially expressed proteins please refer to S1 Table and S2 Table.
(JPG)

S1 File Raw files/Screen shots of MS MS spectra of proteins in graft unions of ‘Super Sunload’ tomato scions grafted on ‘B-blocking’ as tomato rootstock grown under diverse day/night temperatures; normal-standard (23/23°C and 25/18°C) and high-low (30/15°C). (ZIP)

S2 File Raw files/Screen shots of MS MS spectra of proteins in graft unions of ‘‘Super Doterang’ tomato scions grafted on ‘B-blocking’ as tomato rootstock grown under diverse day/night temperatures; normal-standard (23/23°C and 25/18°C) and high-low (30/15°C). (ZIP)

S1 Table Spot quantification analyzed by Progenesis software in graft unions of ‘Super Sunload’ tomato scions grafted on ‘B-blocking’ as tomato rootstock grown under diverse day/night temperatures; normal-standard (23/23°C and 25/18°C) and high-low (30/15°C). (XLSX)

S2 Table Spot quantification analyzed by progenesis software in graft unions of ‘Super Doterang’ tomato scions grafted on ‘B-blocking’ as tomato rootstock grown under diverse day/night temperatures; normal-standard (23/23°C and 25/18°C) and high-low (30/15°C). (XLSX)

S3 Table MS MS ion search in graft unions of ‘Super Sunload’ tomato scions grafted on ‘B-blocking’ as tomato rootstock grown under diverse day/night temperatures; normal-standard (23/23°C and 25/18°C) and high-low (30/15°C). (XLSX)

S4 Table MS MS ion search in graft unions of ‘Super Doterang’ tomato scions grafted on ‘B-blocking’ as tomato rootstock grown under diverse day/night temperatures; normal-standard (23/23°C and 25/18°C) and high-low (30/15°C). (XLSX)

## Abbreviations

ROS: reactive oxygen species
H_2_O_2_: hydrogen peroxide
O_2_^-1^: singlet oxygen
SOD: superoxide dismutase
APX: ascorbate peroxidase
CAT: catalase
2-DE: second dimensional gel electrophoresis
IEF: isoelectric focusing
IPG: immobilized pressure gradient
MALDI-TOF MS: matrix-assisted laser desorption/ionization time of flight mass spectrometer
