# Supplementary figures and images for: Physiological and Proteomic Investigations to Study the Response of Tomato Graft Unions under Temperature Stress
Source: PLoS One. 2016 Jun 16;11(6):e0157439. doi: 10.1371/journal.pone.0157439 (PMC4911148; doi:10.1371/journal.pone.0157439)

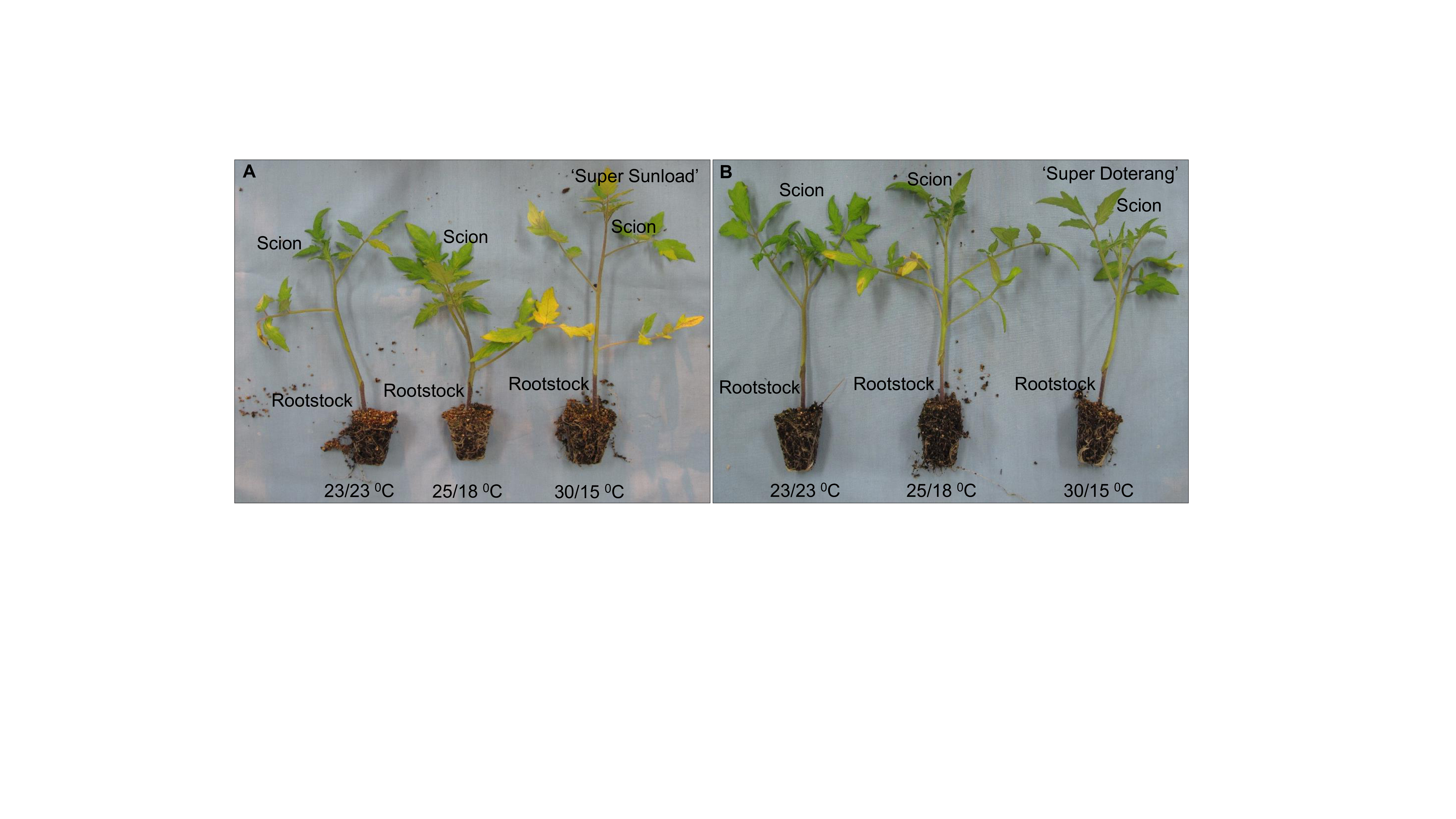

Supplement: S1 Fig — Representative images of grafted tomato plants grown under diverse temperatures (day/night; 23/23 0C, 25/18 0C, and 30/15 0C) under greenhouse conditions (A) ‘B-blocking’ used as rootstock and ‘Super Sunload’ as scion (B) ‘B-blocking’ used as rootstock and ‘Super Doterang’ as scion. (JPG) [file pone.0157439.s001.jpg]

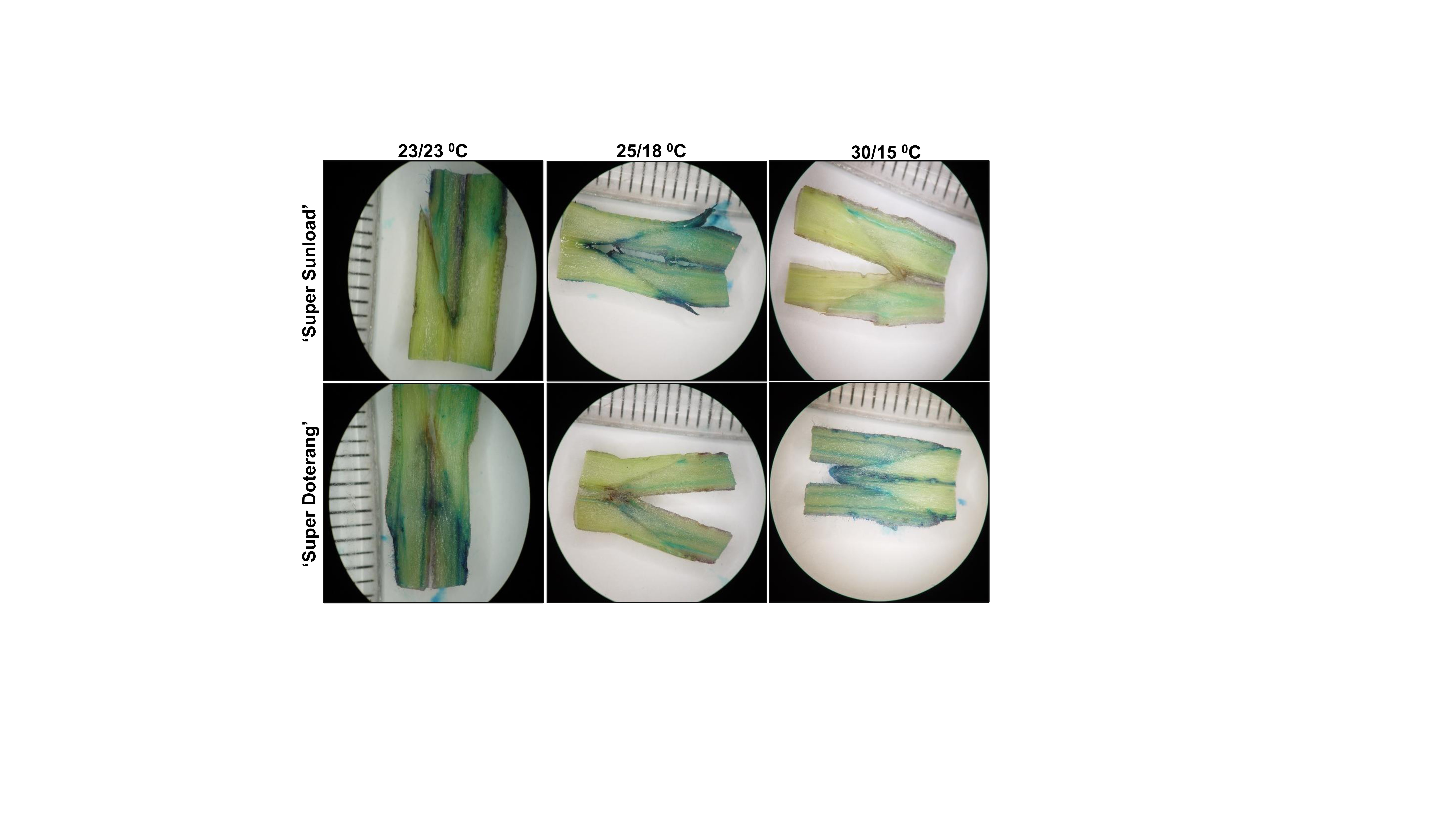

Supplement: S2 Fig — The rootstocks were submerged in absorbable flower dye blue for 10–20 min and rootstock as well as scions were cut into transverse sections with razor blade. The cross sections were observed under light microscope. (JPG) [file pone.0157439.s002.jpg]

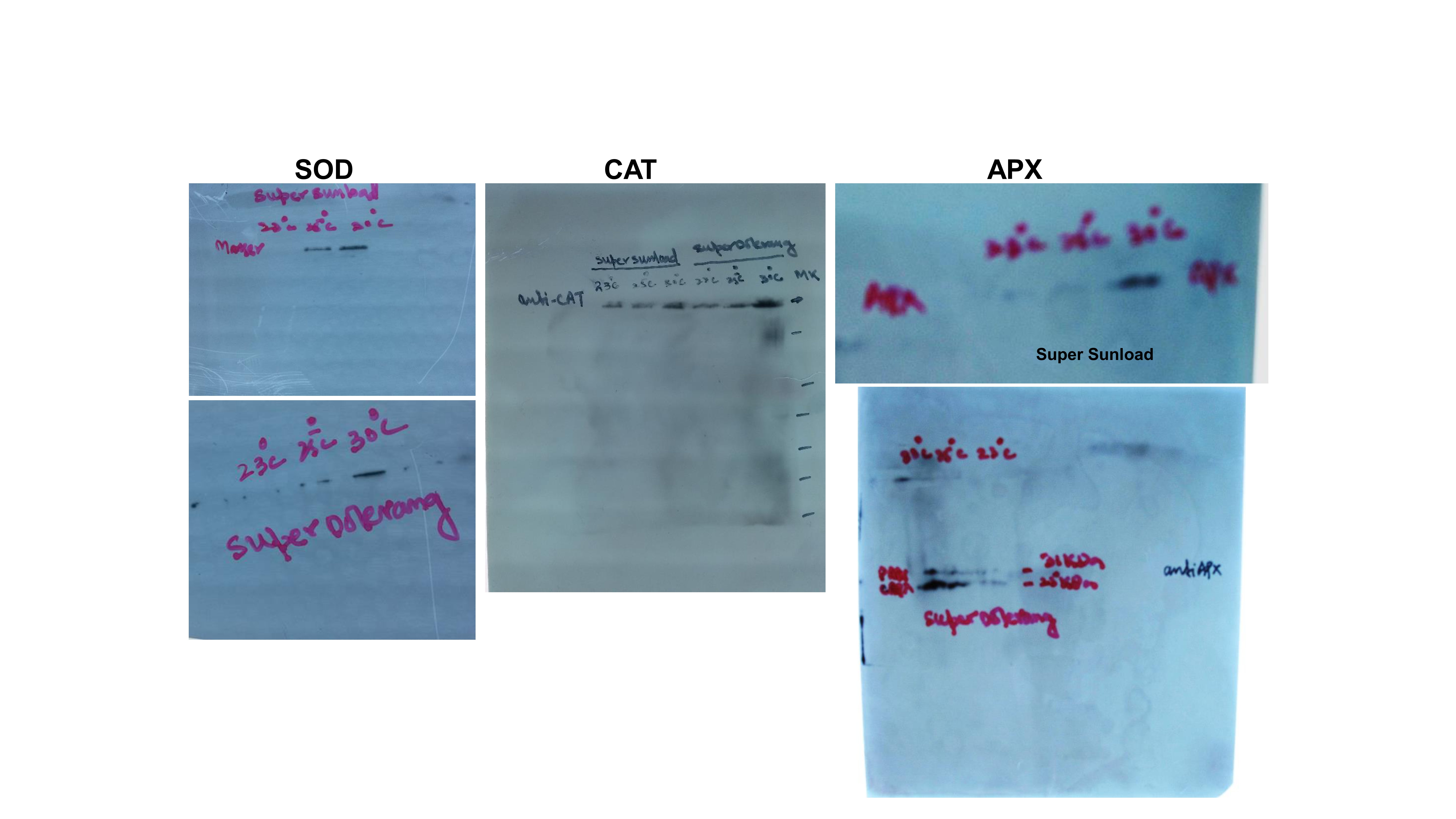

Supplement: S3 Fig — (Note: About 2–3 cm graft union (rootstock/scion) were cut near the junction and were used for the analysis). (JPG) [file pone.0157439.s003.jpg]

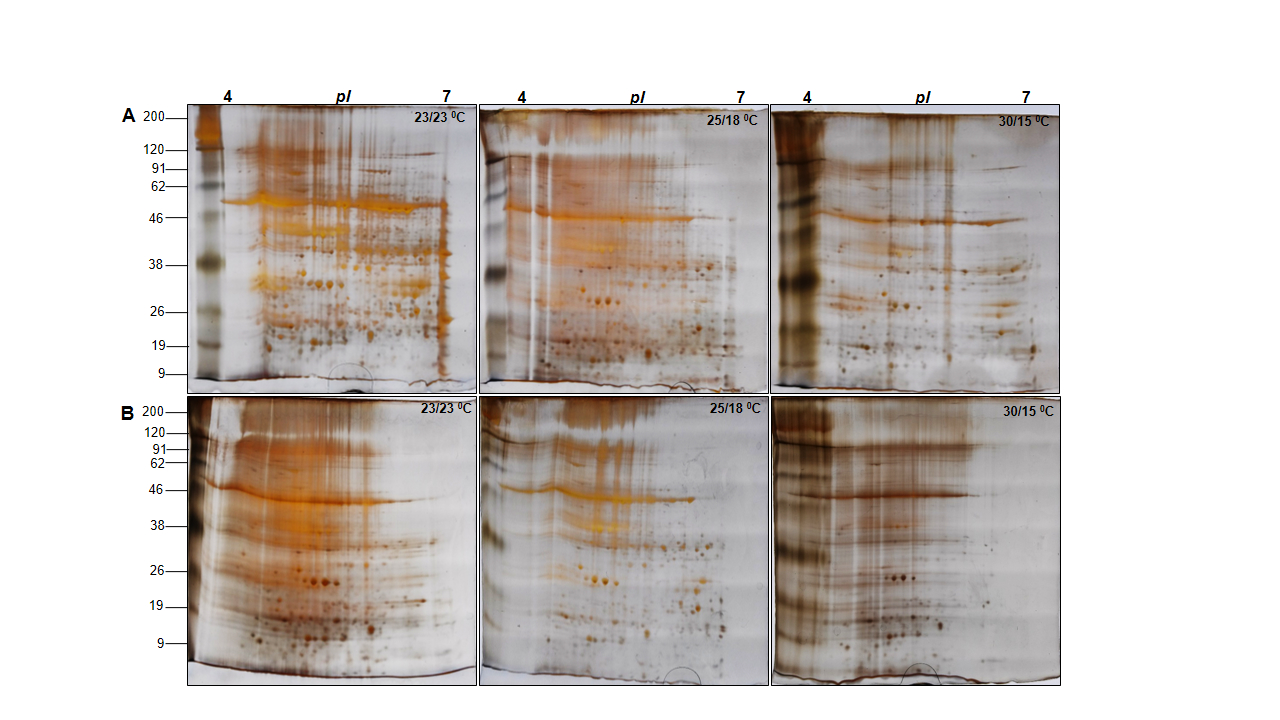

Supplement: S4 Fig — Representative raw data of 2-DE gels in graft unions (A) ‘Super Sunload’ (B) ‘Super Doterang’ tomato scions grafted on ‘B-blocking’ as tomato rootstock grown under diverse day/night temperatures; normal-standard (23/23°C and 25/18°C) and high-low (30/15°C). About 70 μg of proteins from 2–3 cm graft unions were focused on 11 cm IPG strips (pI 4–7) for first dimension and separated on 12.5% (w/v) polyacrylamide gels (SDS-PAGE) for second dimension. (JPG) [file pone.0157439.s004.jpg]

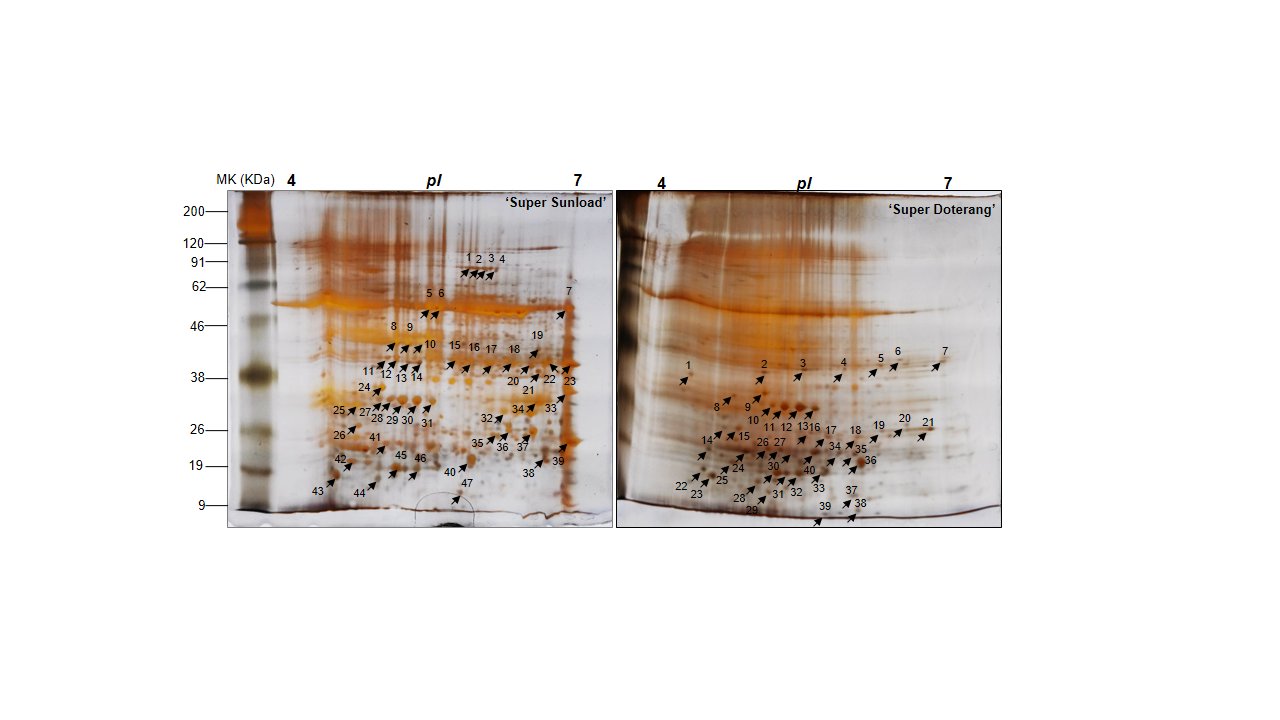

Supplement: S5 Fig — The match set was created from three standard gels for each time point of 2-DE gels as shown in Fig 8. The numbers on the gel indicate differentially expressed proteins. For descriptive quantification of differentially expressed proteins please refer to S1 Table and S2 Table. (JPG) [file pone.0157439.s005.jpg]

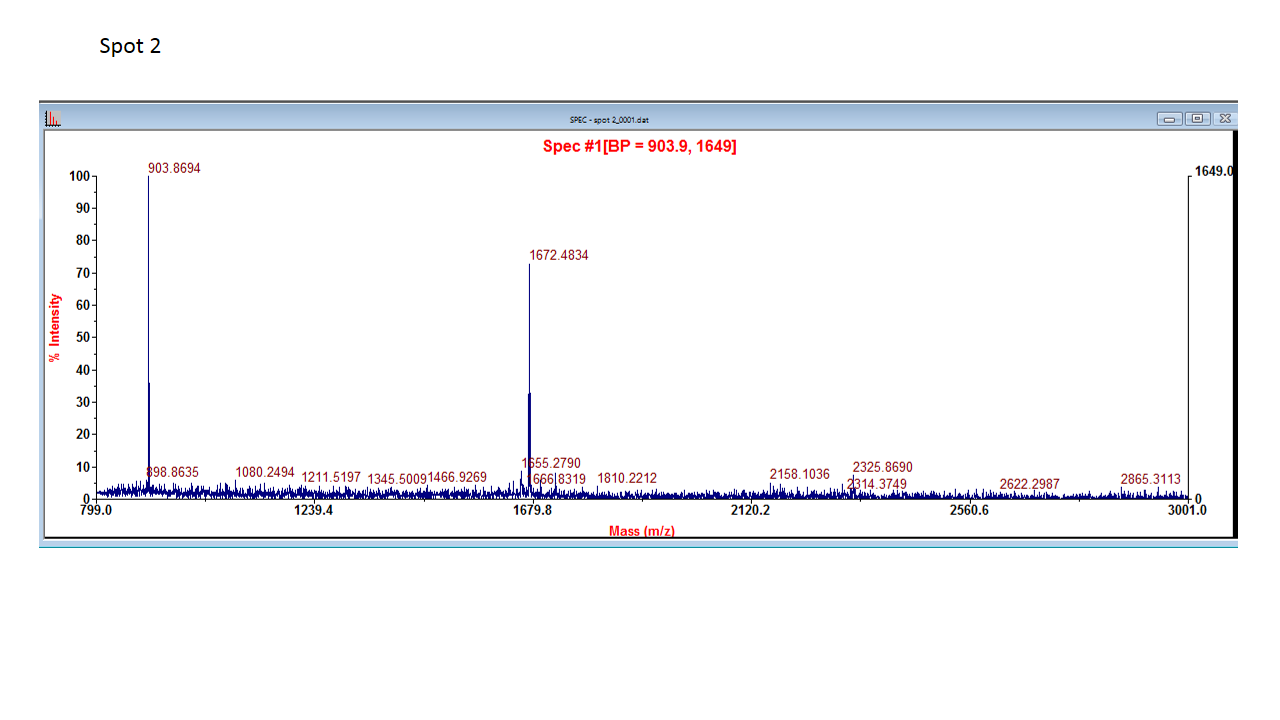

Supplement: S1 File — (ZIP) [file pone.0157439.s006.zip › Slide1.TIF]

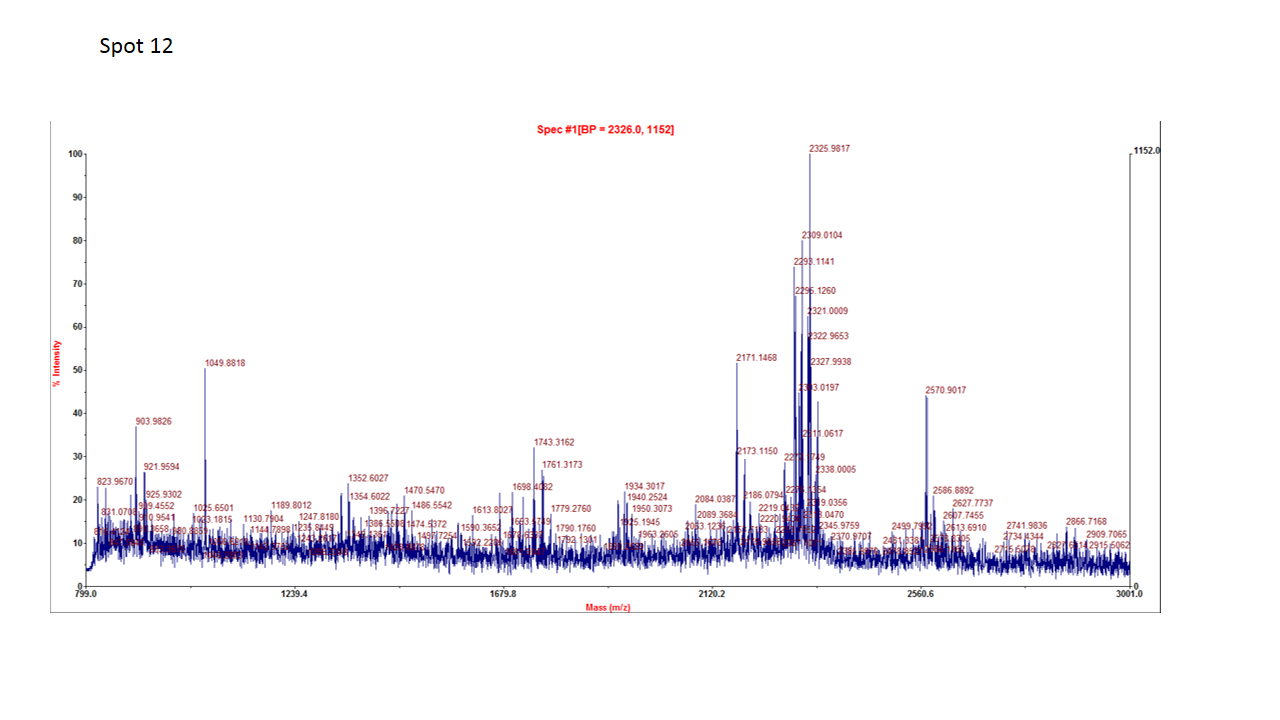

Supplement: S1 File — (ZIP) [file pone.0157439.s006.zip › Slide10.TIF]

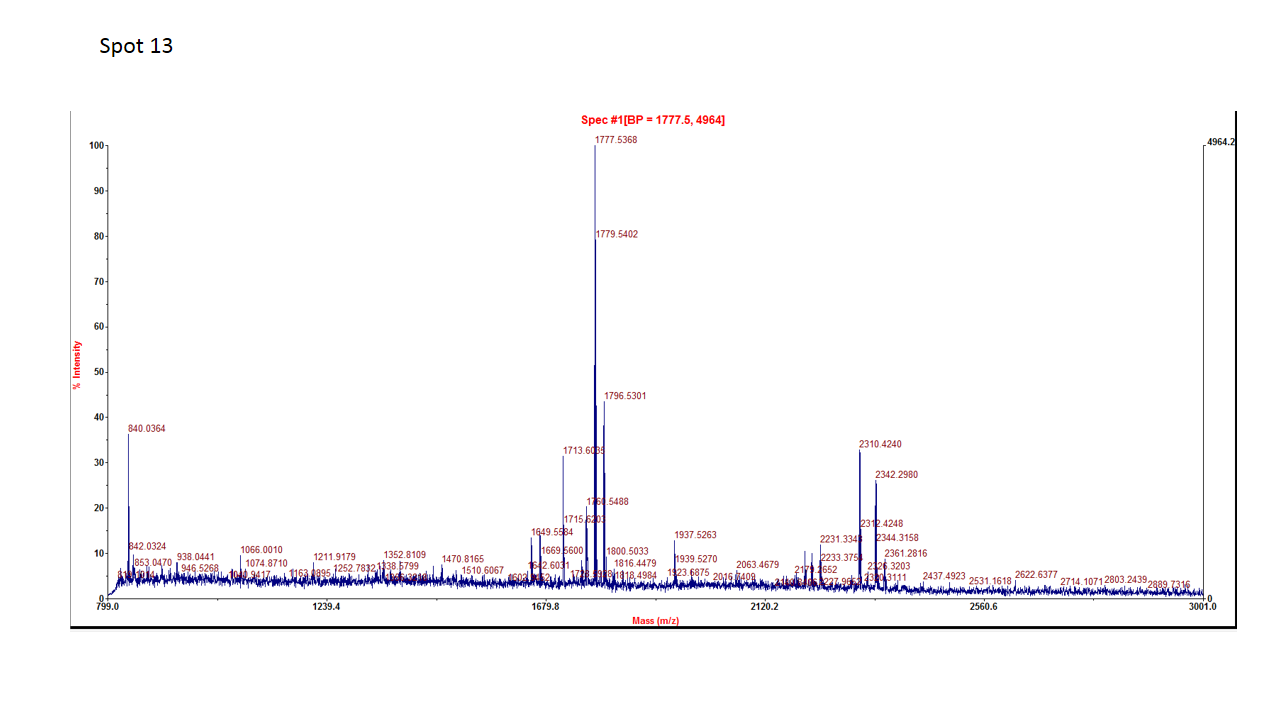

Supplement: S1 File — (ZIP) [file pone.0157439.s006.zip › Slide11.TIF]

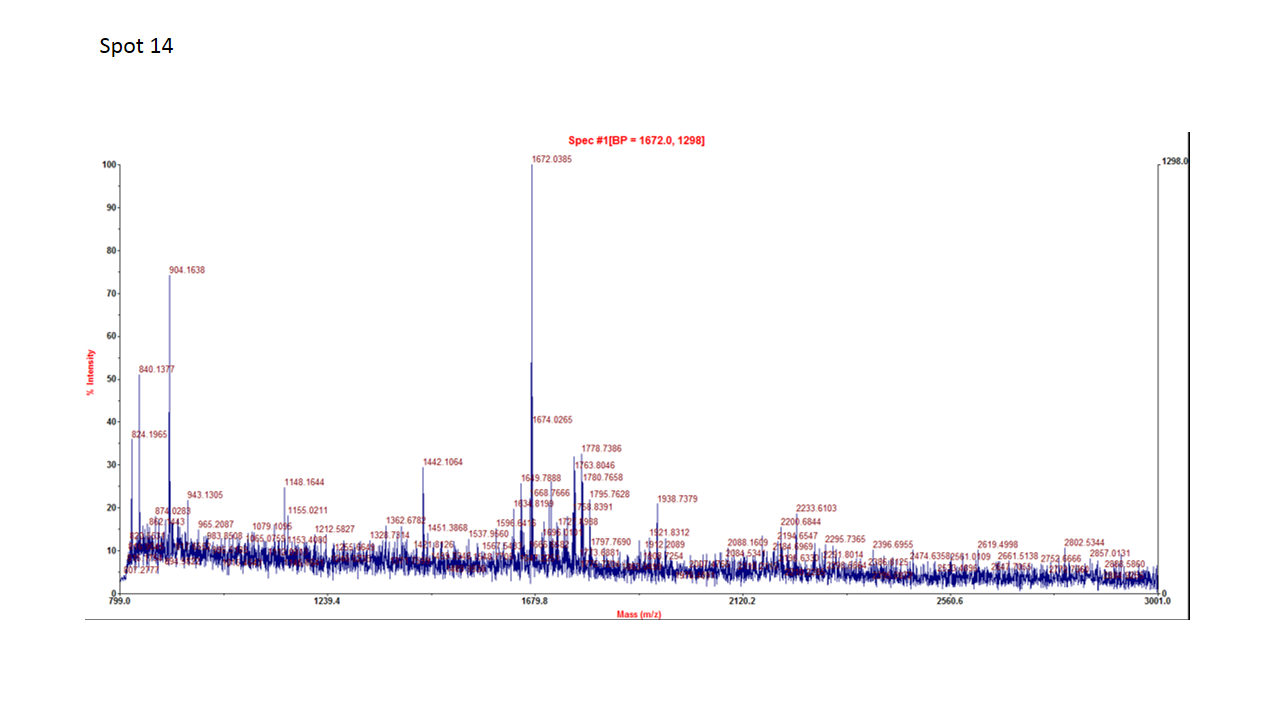

Supplement: S1 File — (ZIP) [file pone.0157439.s006.zip › Slide12.TIF]

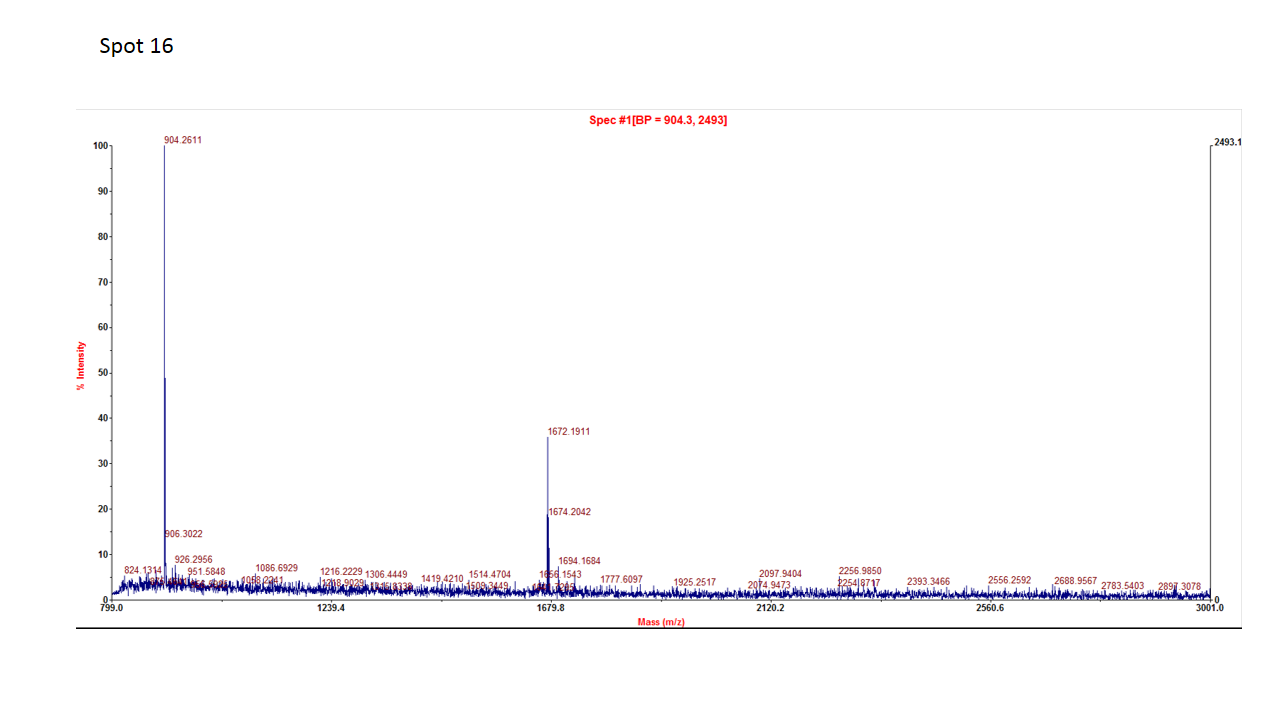

Supplement: S1 File — (ZIP) [file pone.0157439.s006.zip › Slide13.TIF]

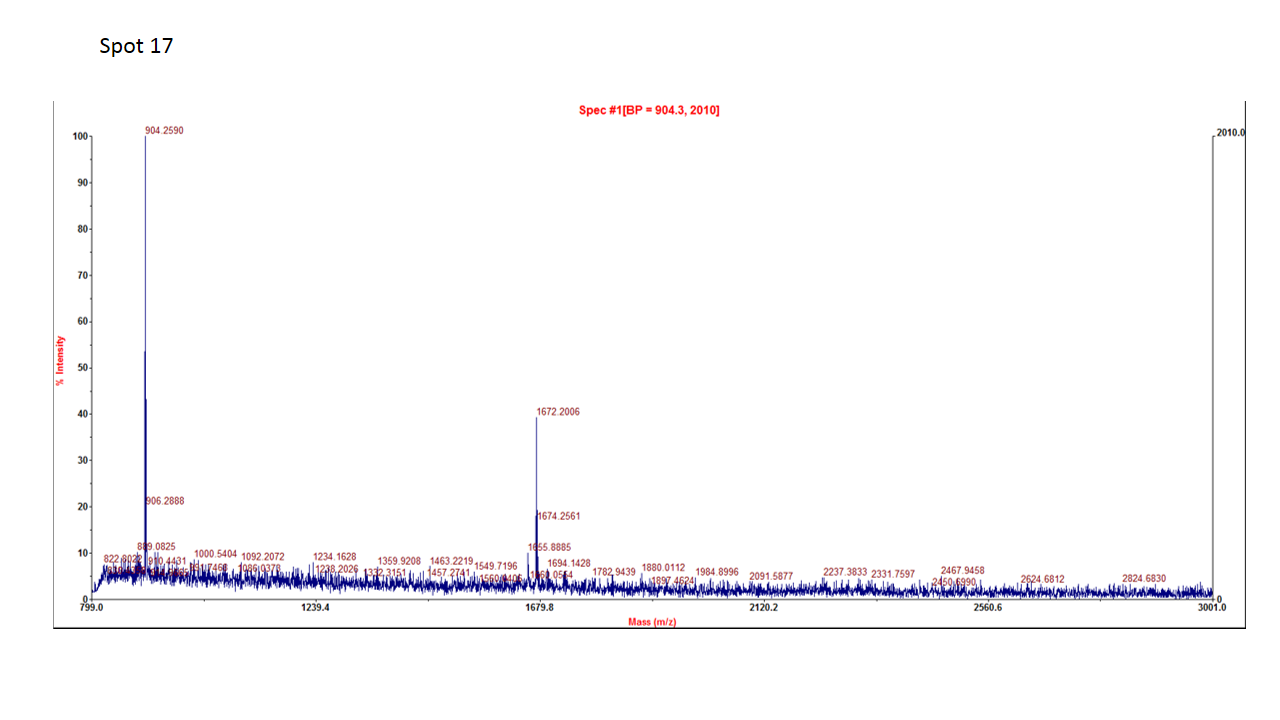

Supplement: S1 File — (ZIP) [file pone.0157439.s006.zip › Slide14.TIF]

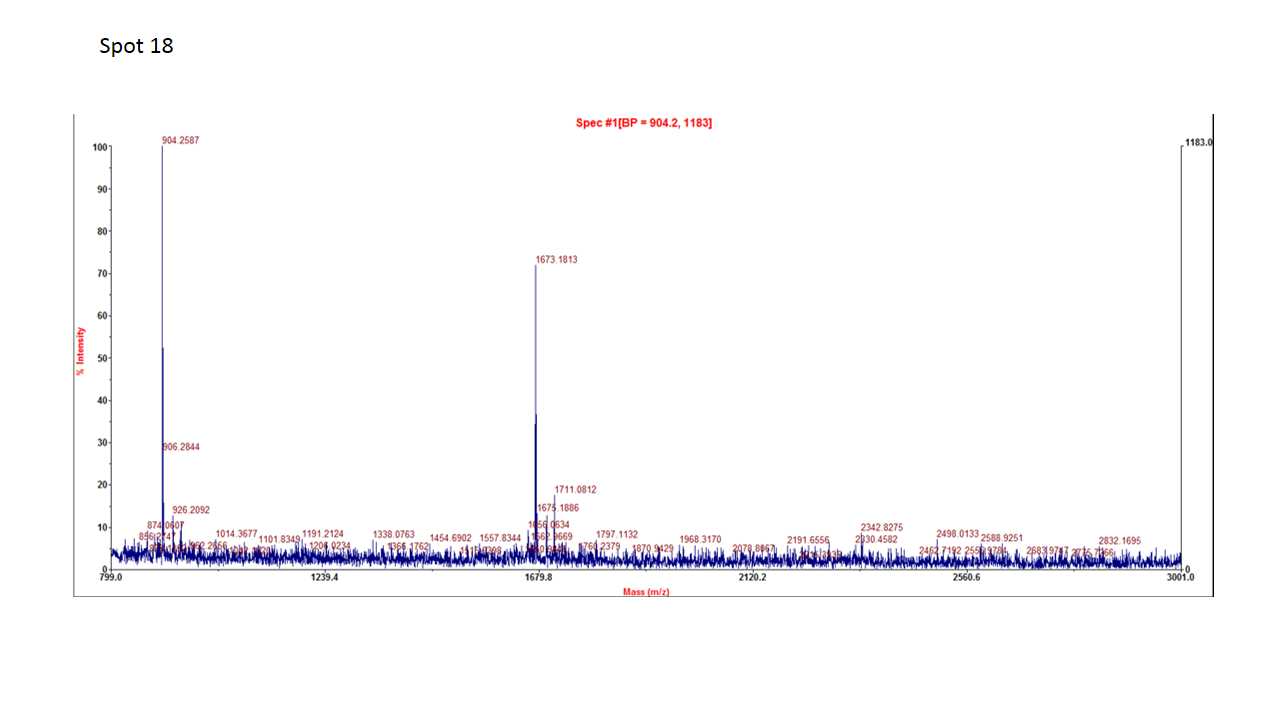

Supplement: S1 File — (ZIP) [file pone.0157439.s006.zip › Slide15.TIF]

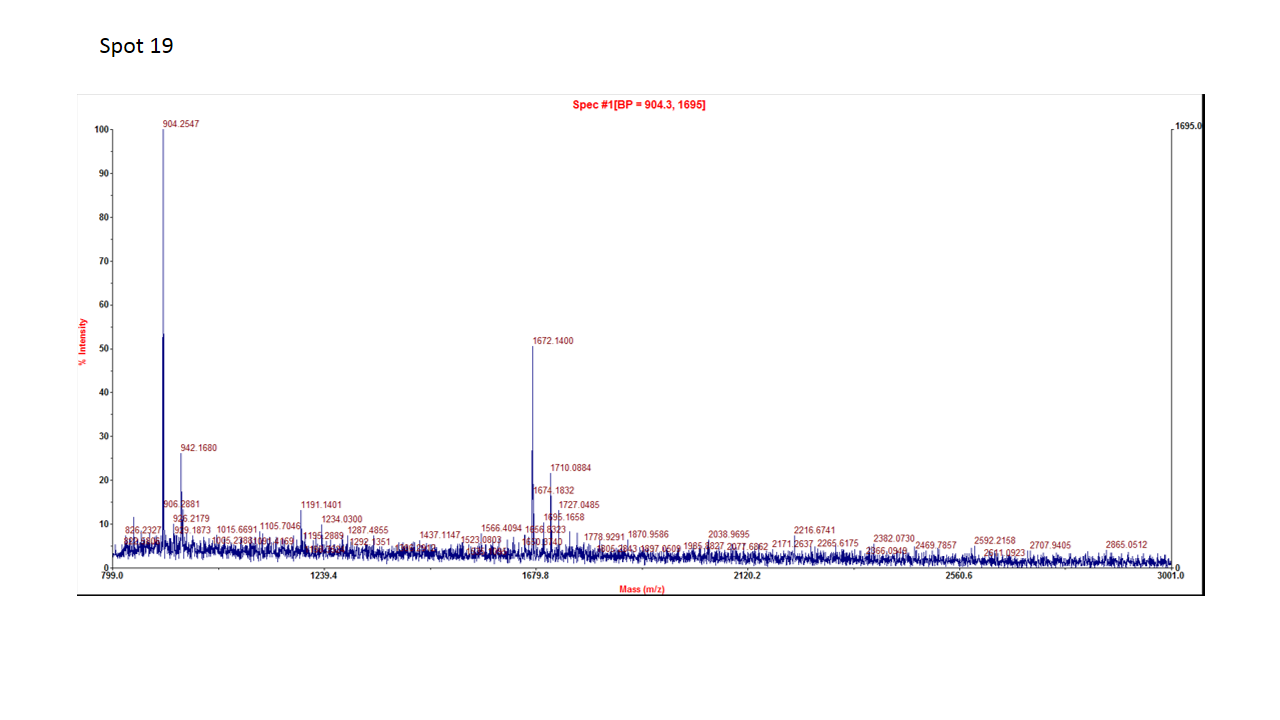

Supplement: S1 File — (ZIP) [file pone.0157439.s006.zip › Slide16.TIF]

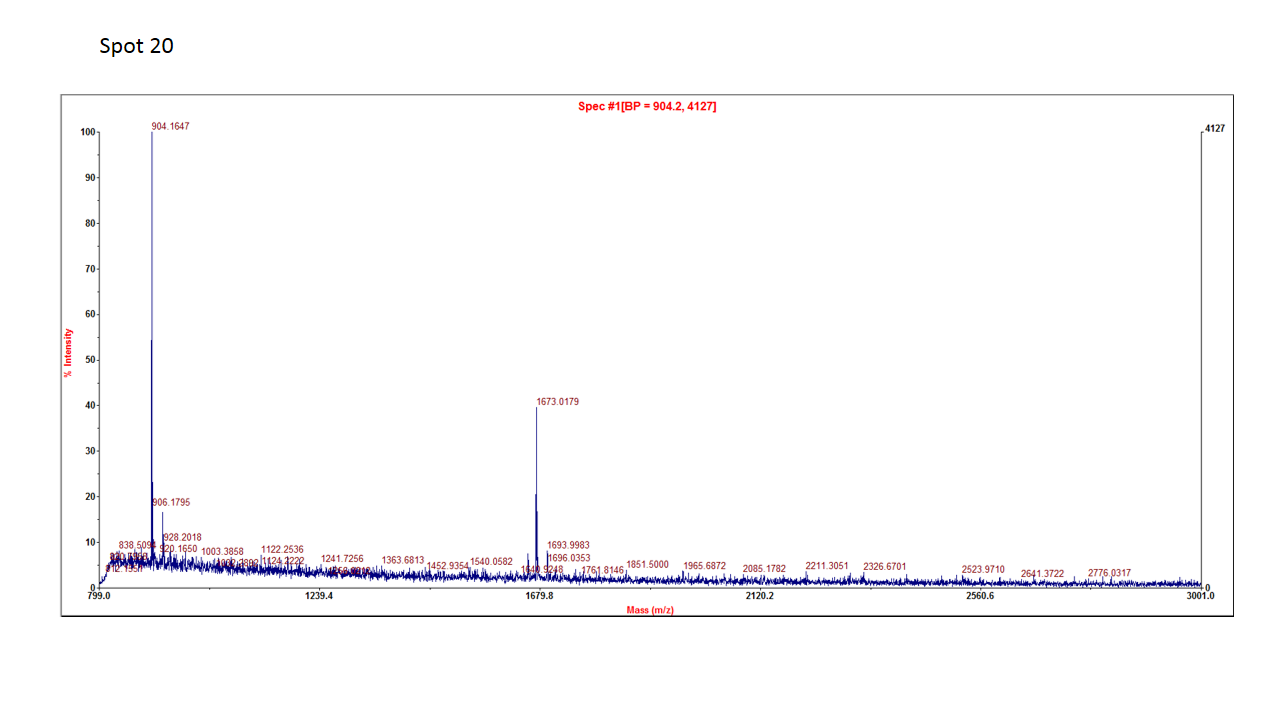

Supplement: S1 File — (ZIP) [file pone.0157439.s006.zip › Slide17.TIF]

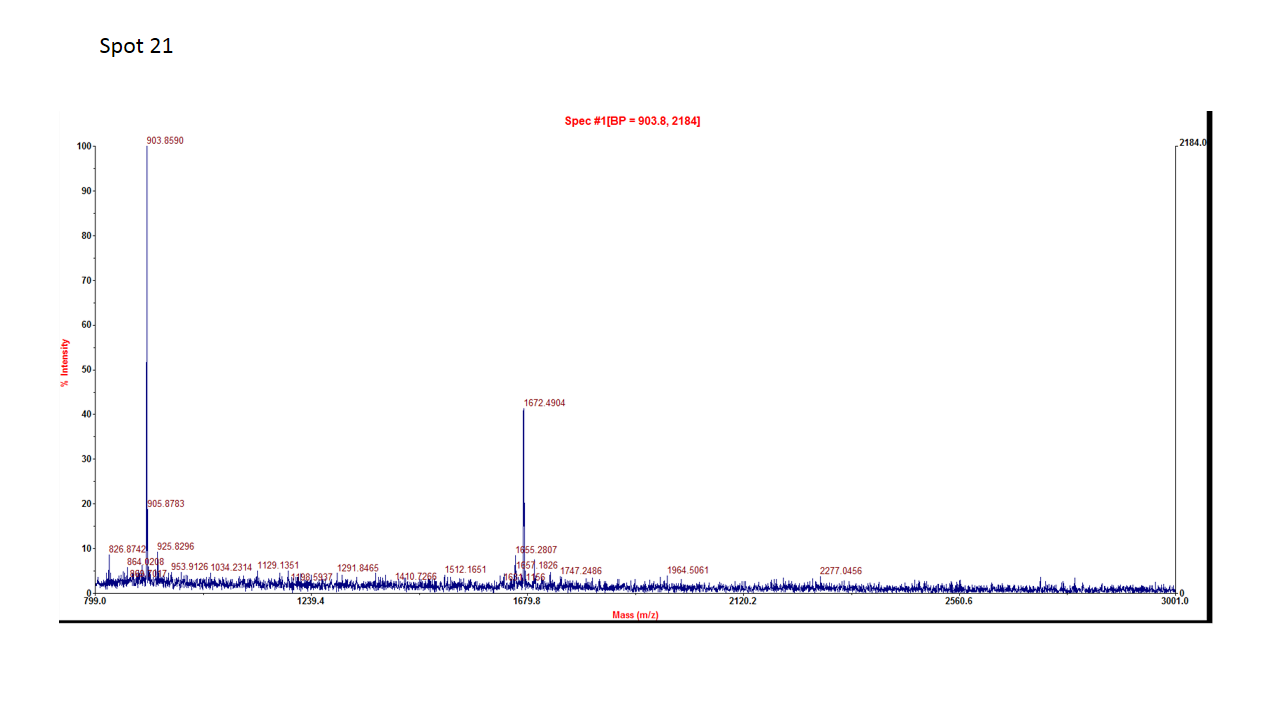

Supplement: S1 File — (ZIP) [file pone.0157439.s006.zip › Slide18.TIF]

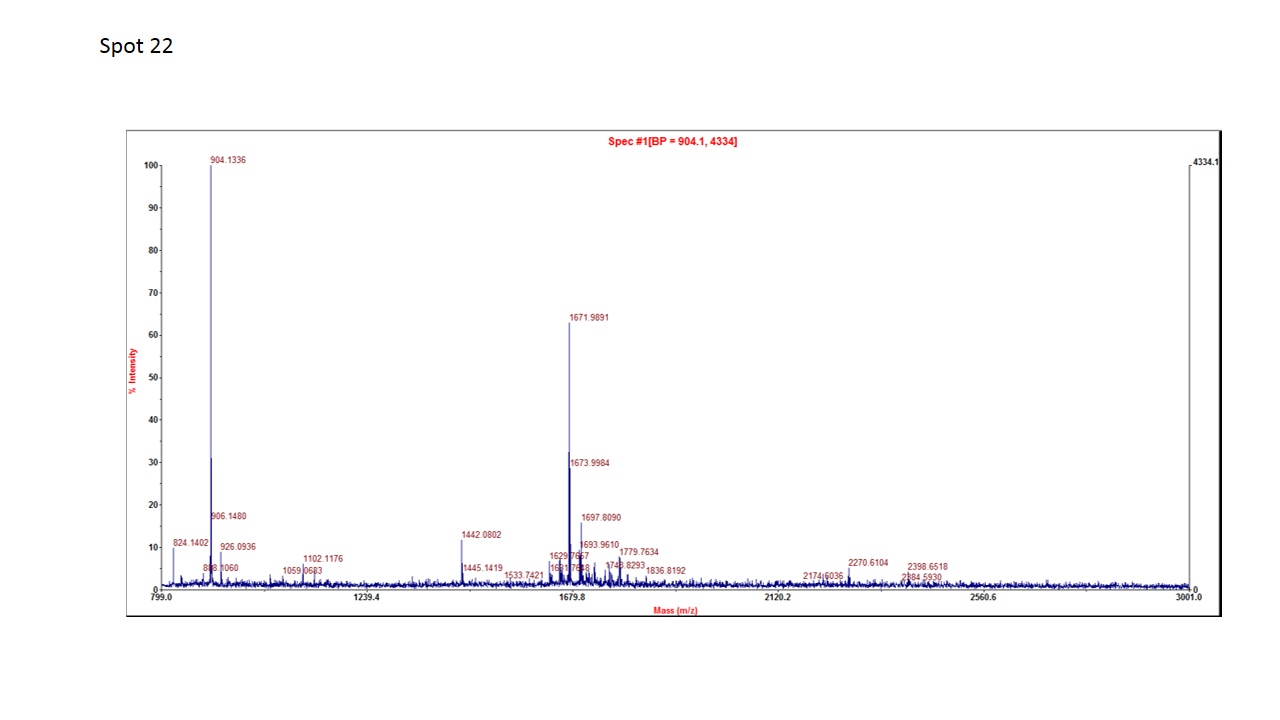

Supplement: S1 File — (ZIP) [file pone.0157439.s006.zip › Slide19.TIF]

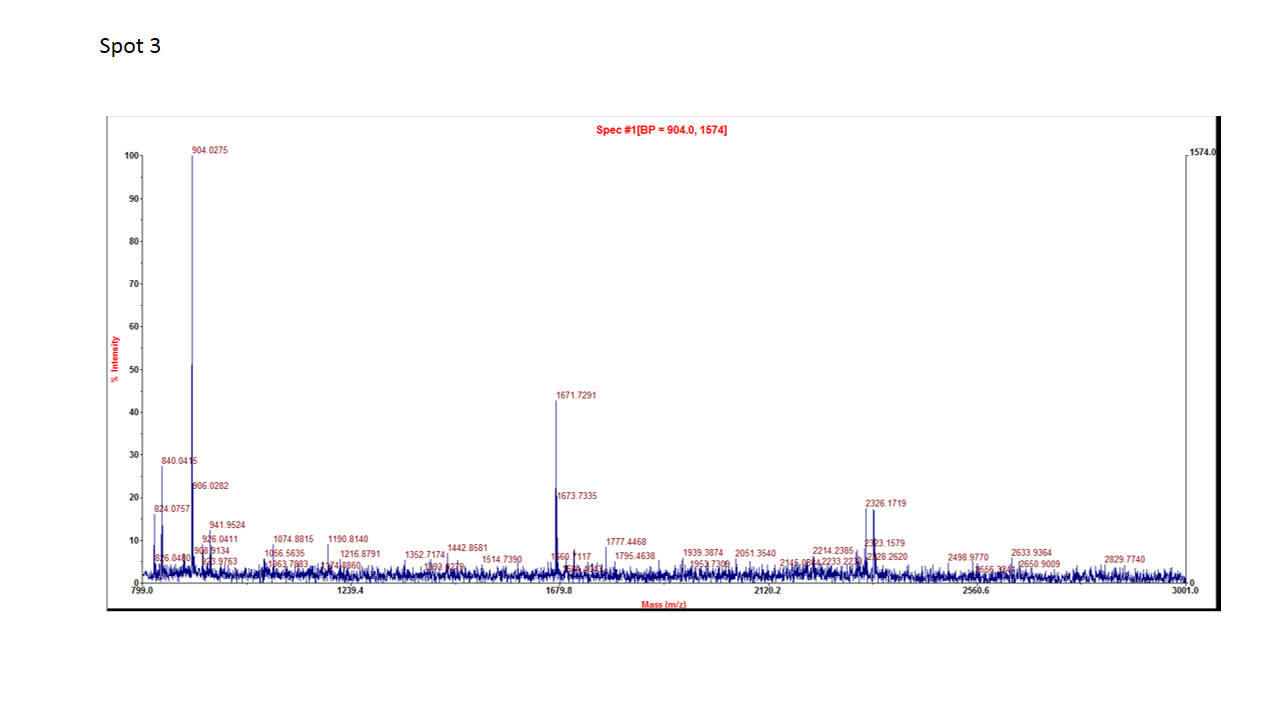

Supplement: S1 File — (ZIP) [file pone.0157439.s006.zip › Slide2.TIF]

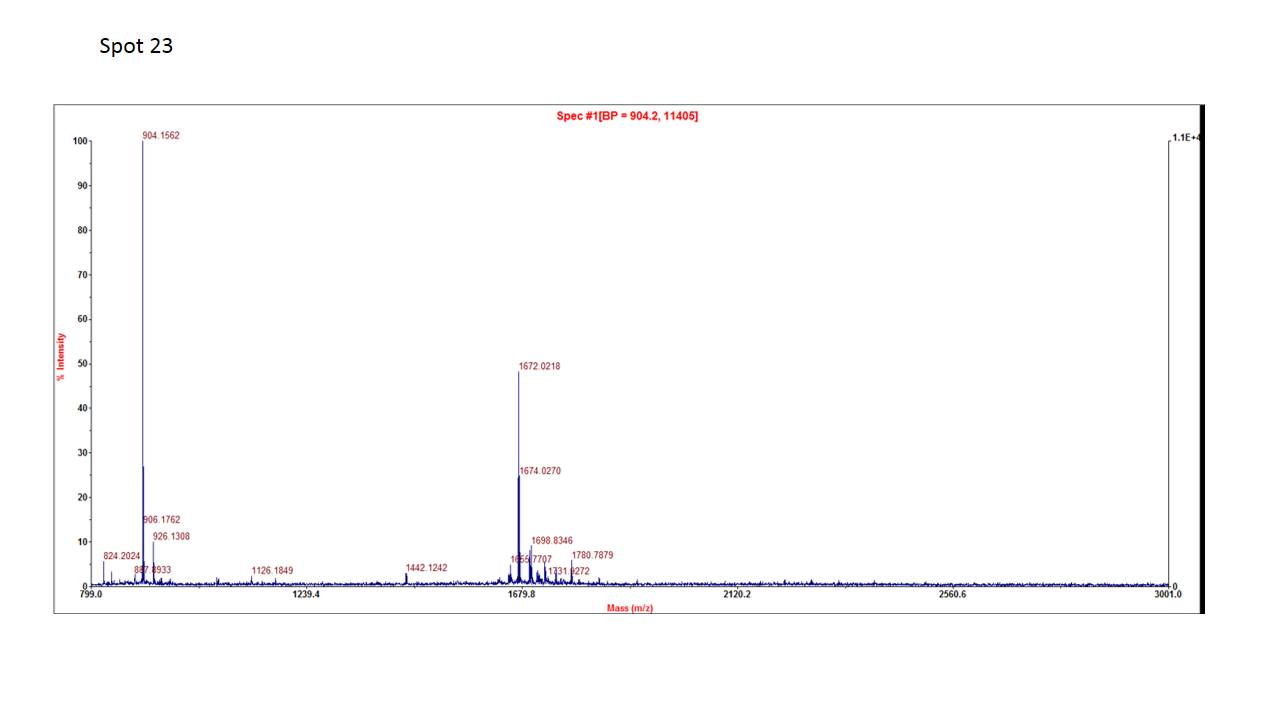

Supplement: S1 File — (ZIP) [file pone.0157439.s006.zip › Slide20.TIF]

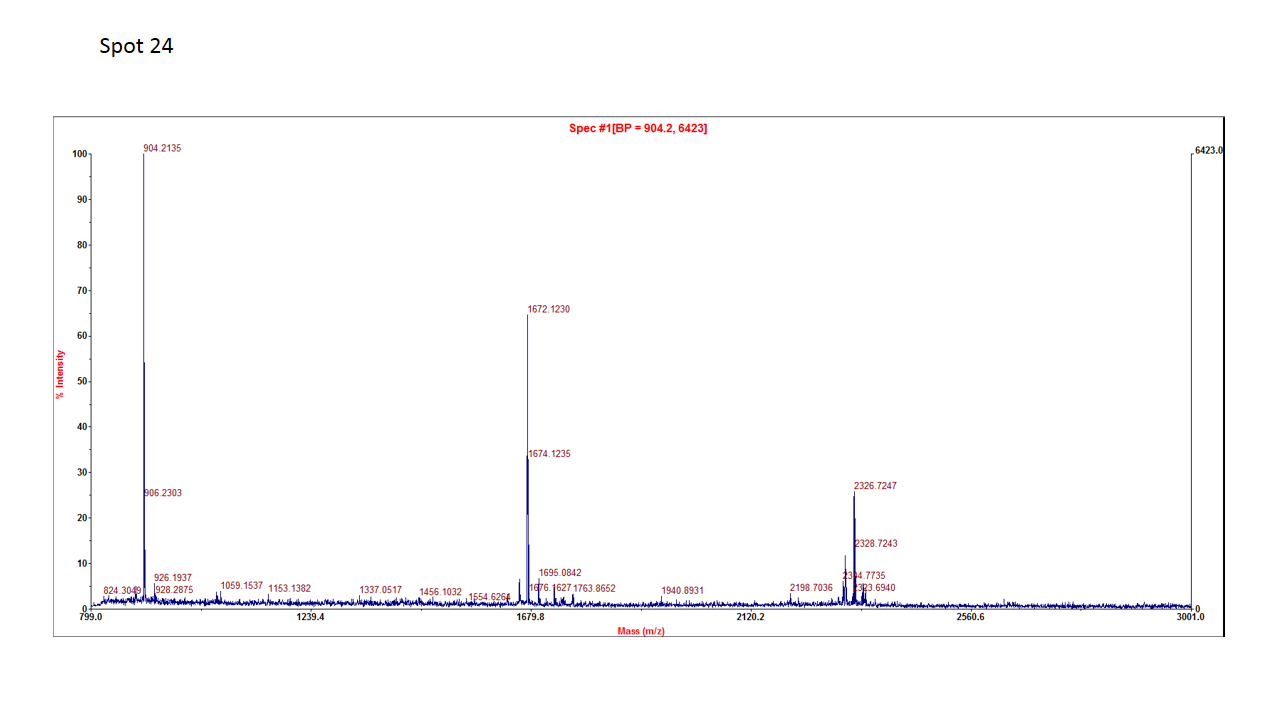

Supplement: S1 File — (ZIP) [file pone.0157439.s006.zip › Slide21.TIF]

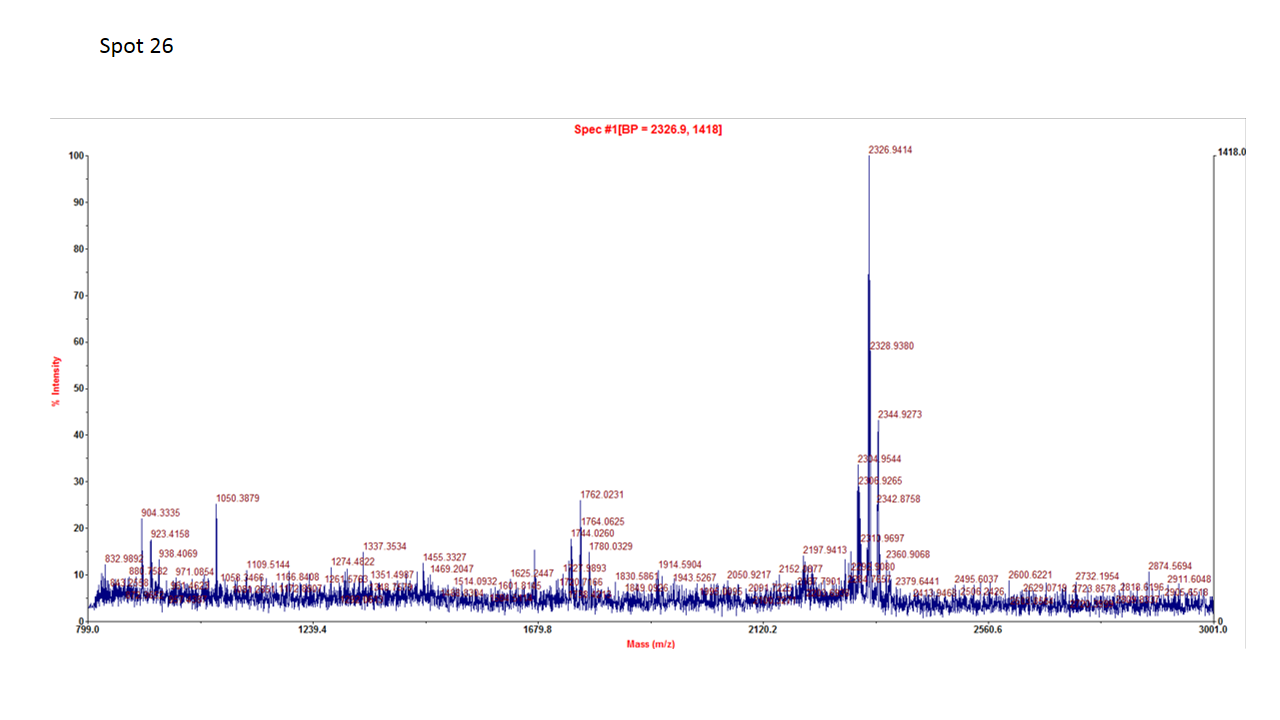

Supplement: S1 File — (ZIP) [file pone.0157439.s006.zip › Slide22.TIF]

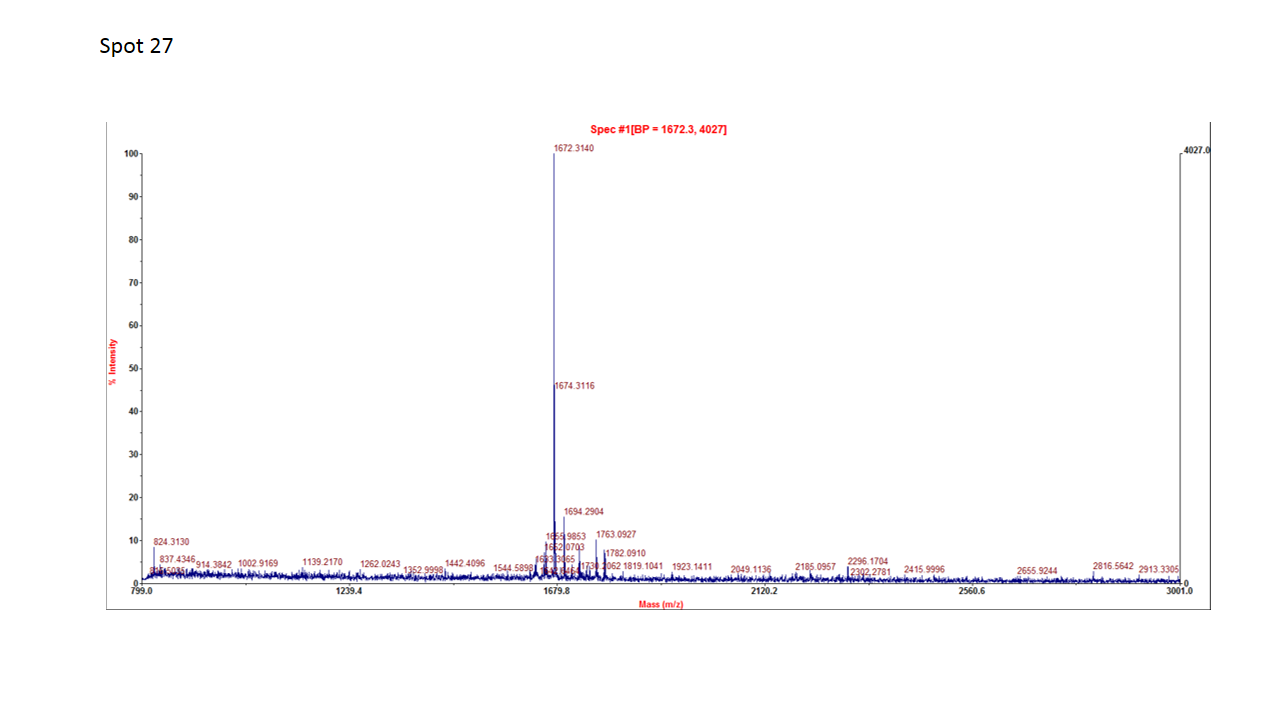

Supplement: S1 File — (ZIP) [file pone.0157439.s006.zip › Slide23.TIF]

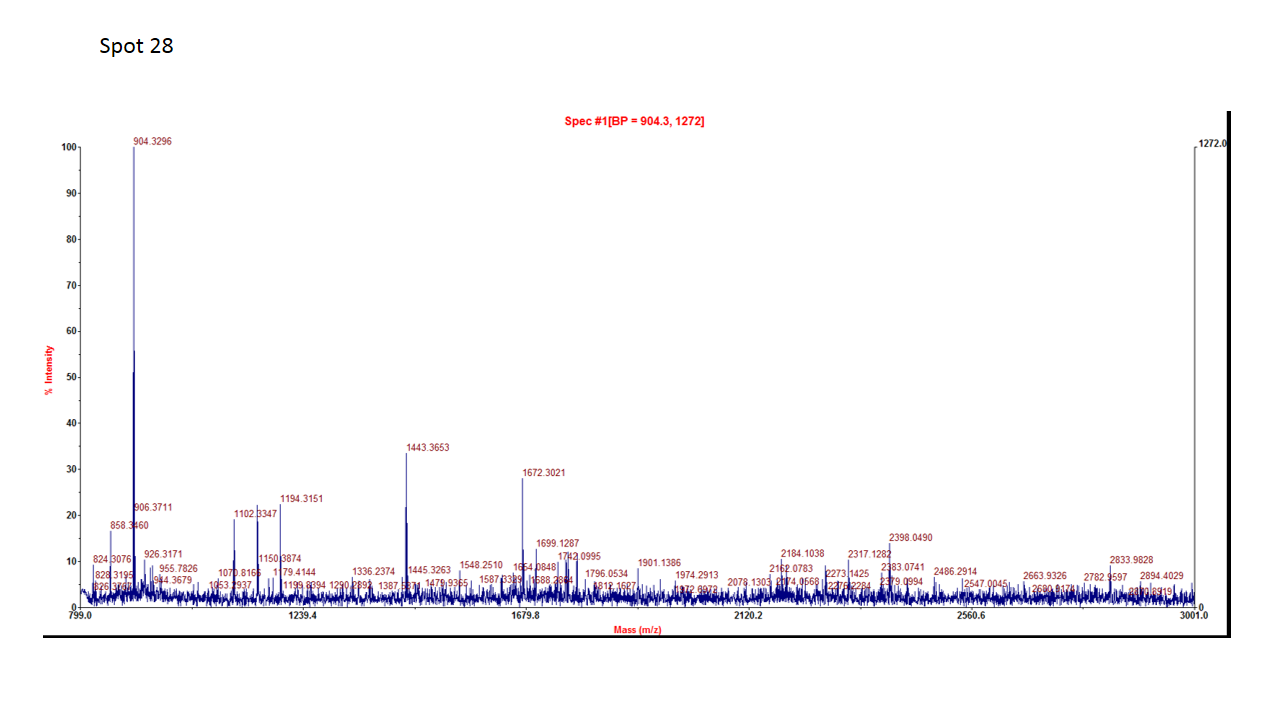

Supplement: S1 File — (ZIP) [file pone.0157439.s006.zip › Slide24.TIF]

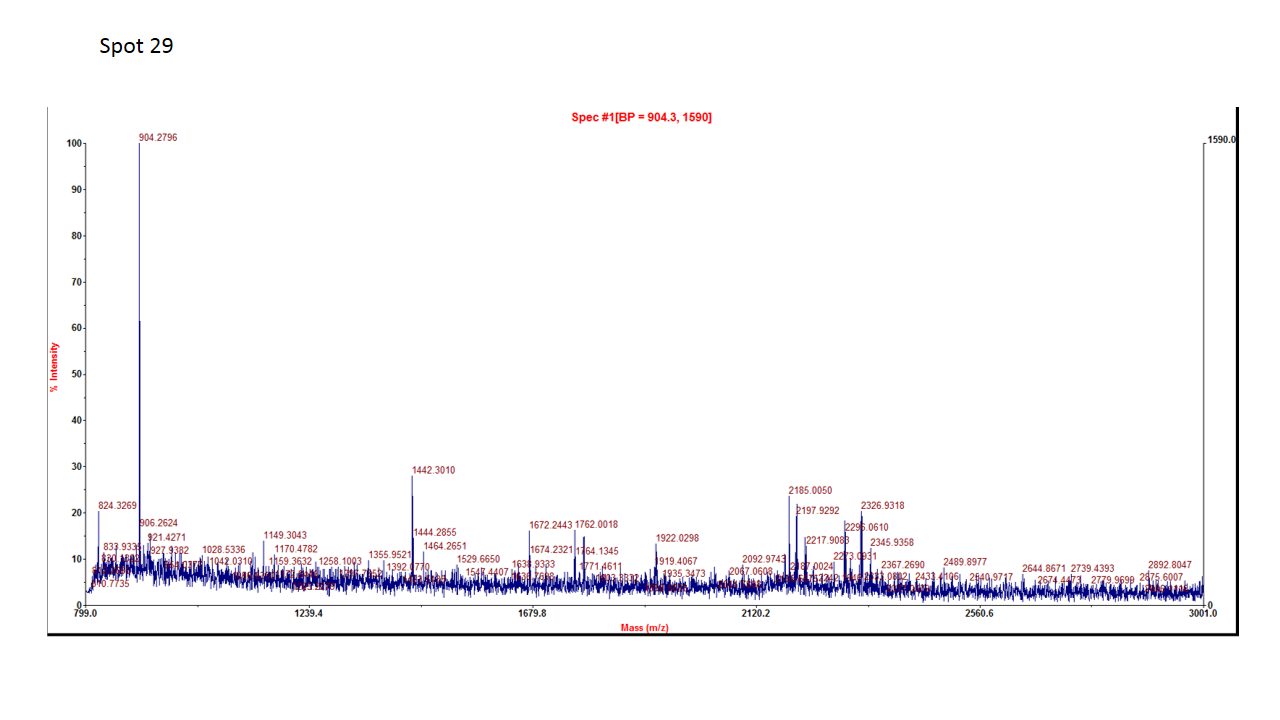

Supplement: S1 File — (ZIP) [file pone.0157439.s006.zip › Slide25.TIF]

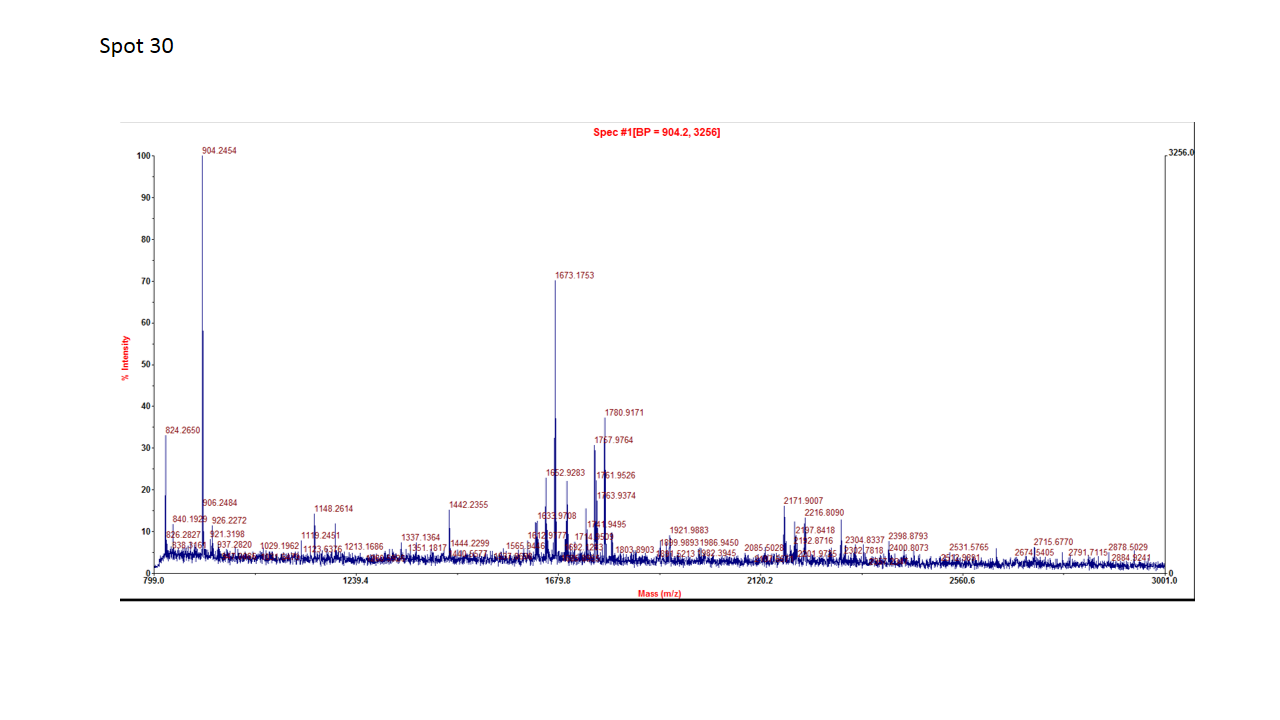

Supplement: S1 File — (ZIP) [file pone.0157439.s006.zip › Slide26.TIF]

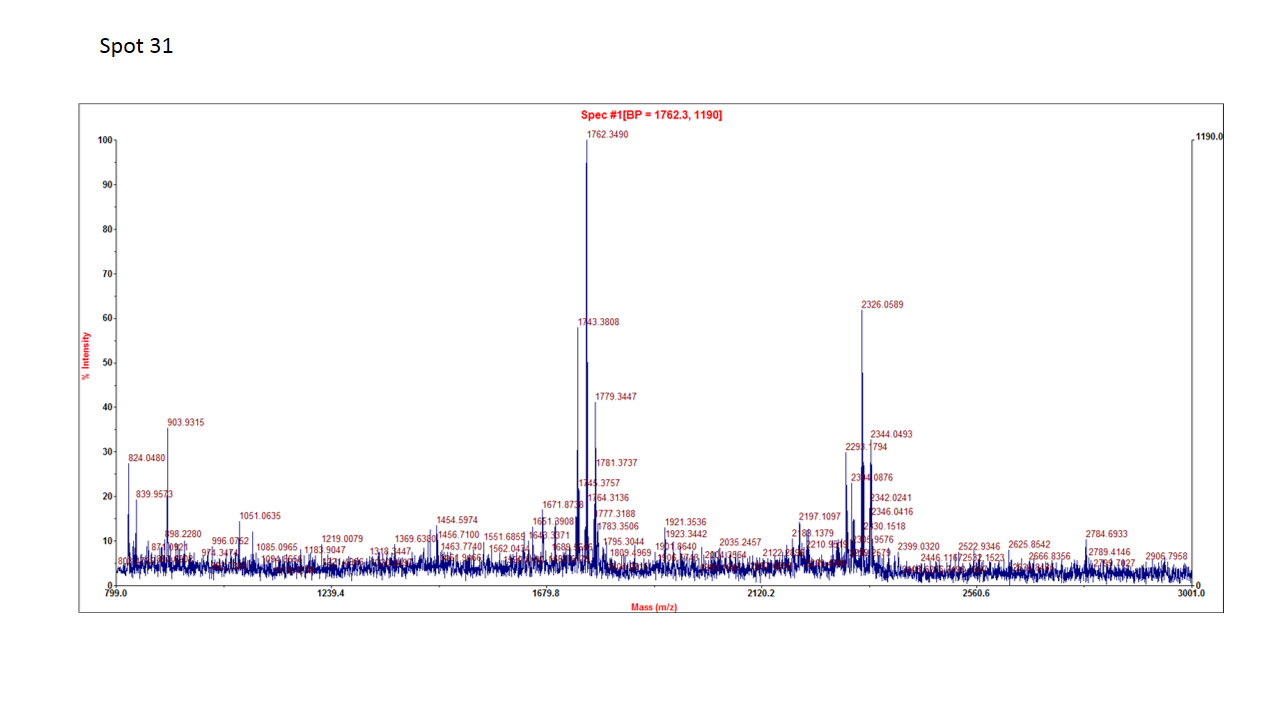

Supplement: S1 File — (ZIP) [file pone.0157439.s006.zip › Slide27.TIF]

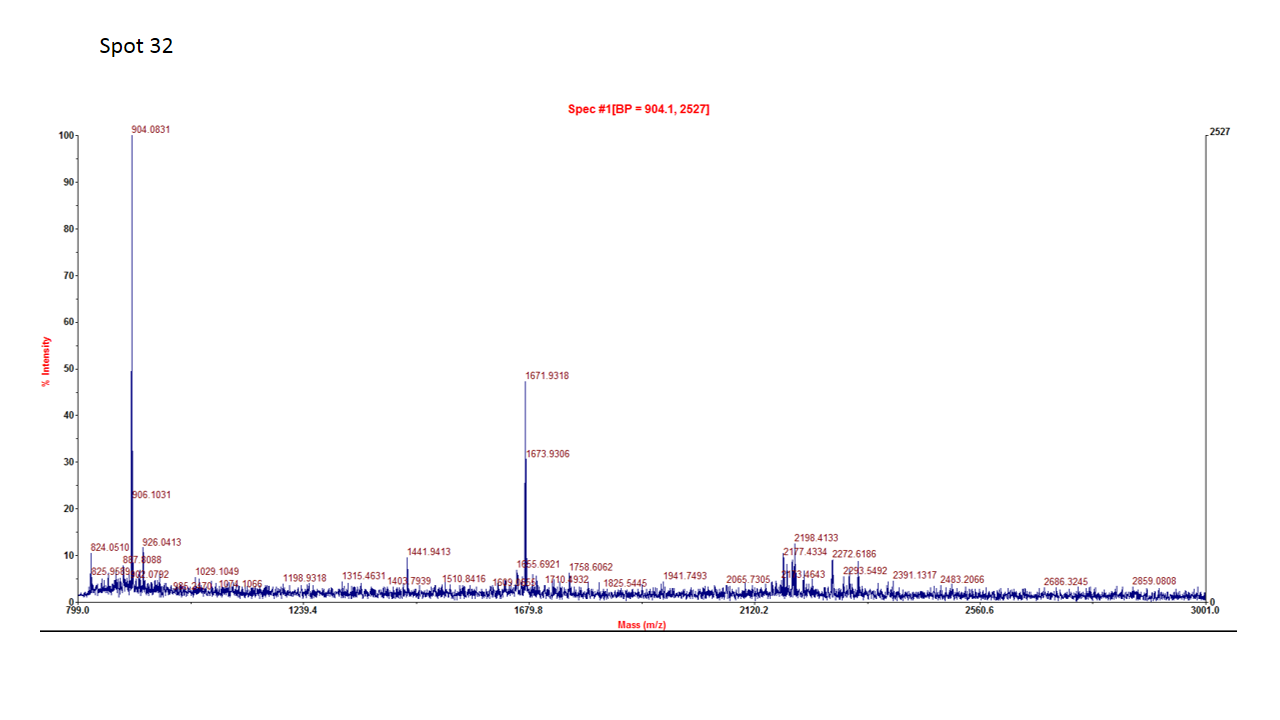

Supplement: S1 File — (ZIP) [file pone.0157439.s006.zip › Slide28.TIF]

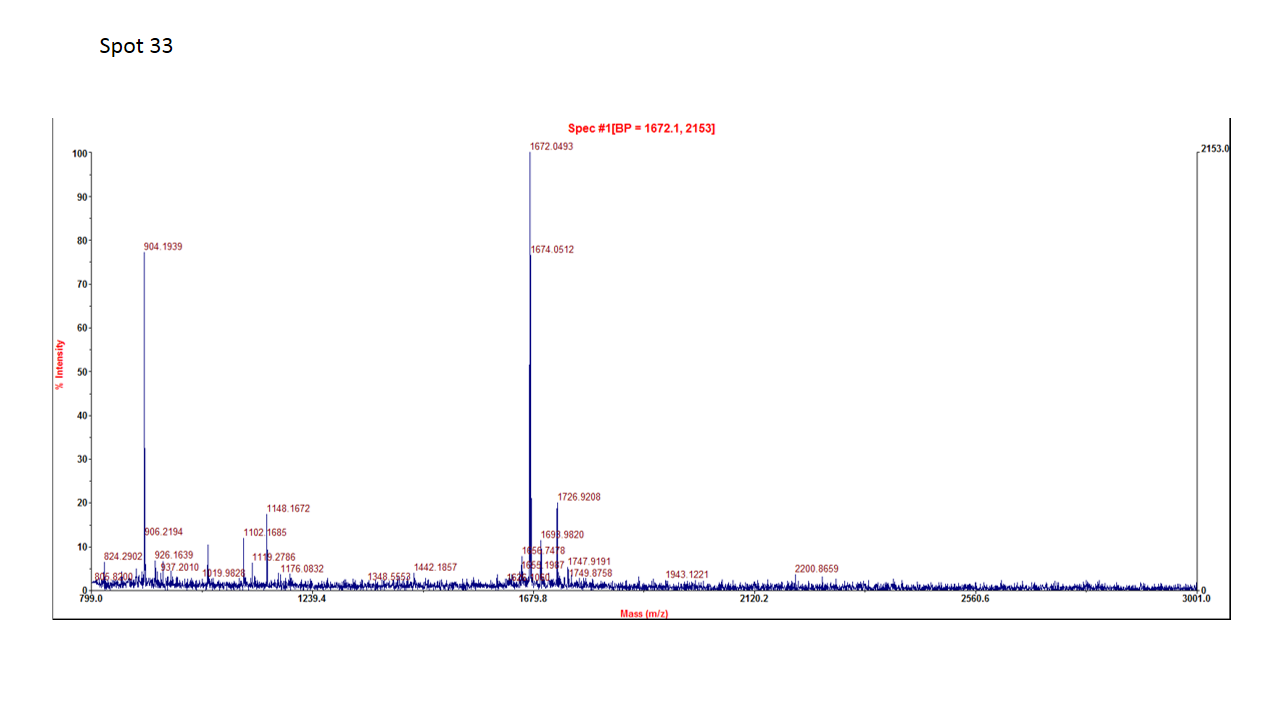

Supplement: S1 File — (ZIP) [file pone.0157439.s006.zip › Slide29.TIF]

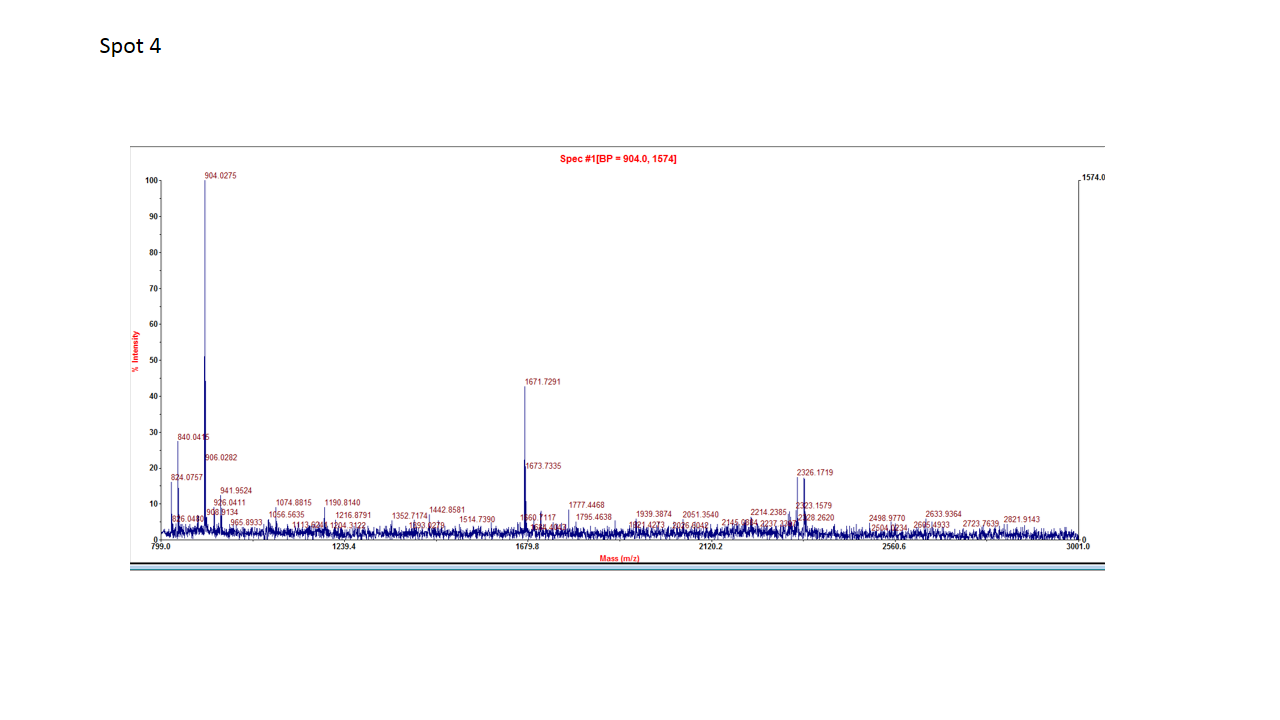

Supplement: S1 File — (ZIP) [file pone.0157439.s006.zip › Slide3.TIF]

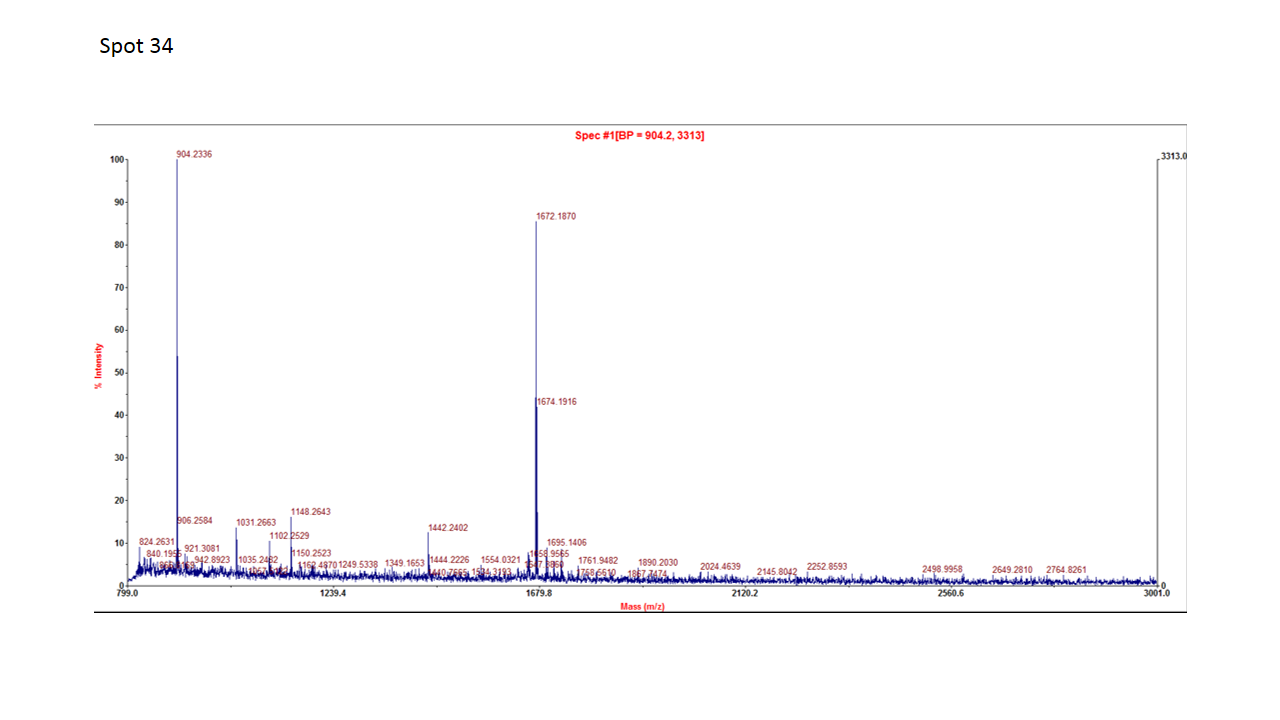

Supplement: S1 File — (ZIP) [file pone.0157439.s006.zip › Slide30.TIF]

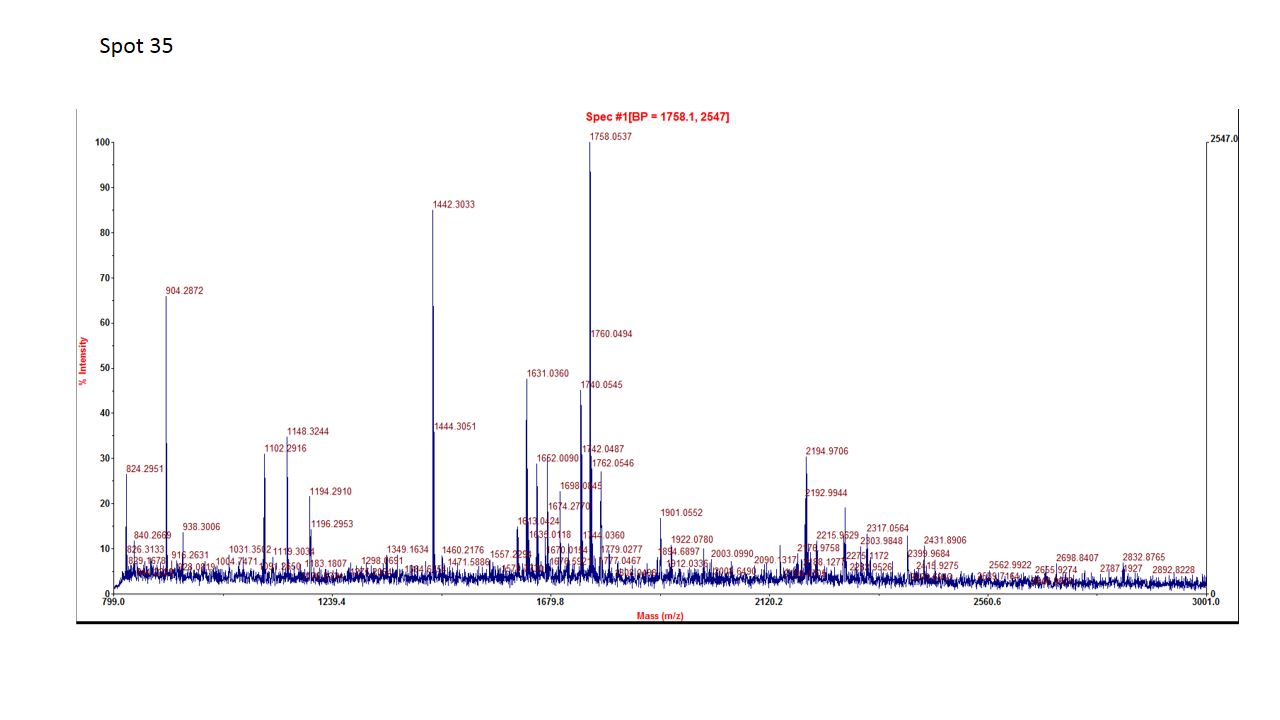

Supplement: S1 File — (ZIP) [file pone.0157439.s006.zip › Slide31.TIF]

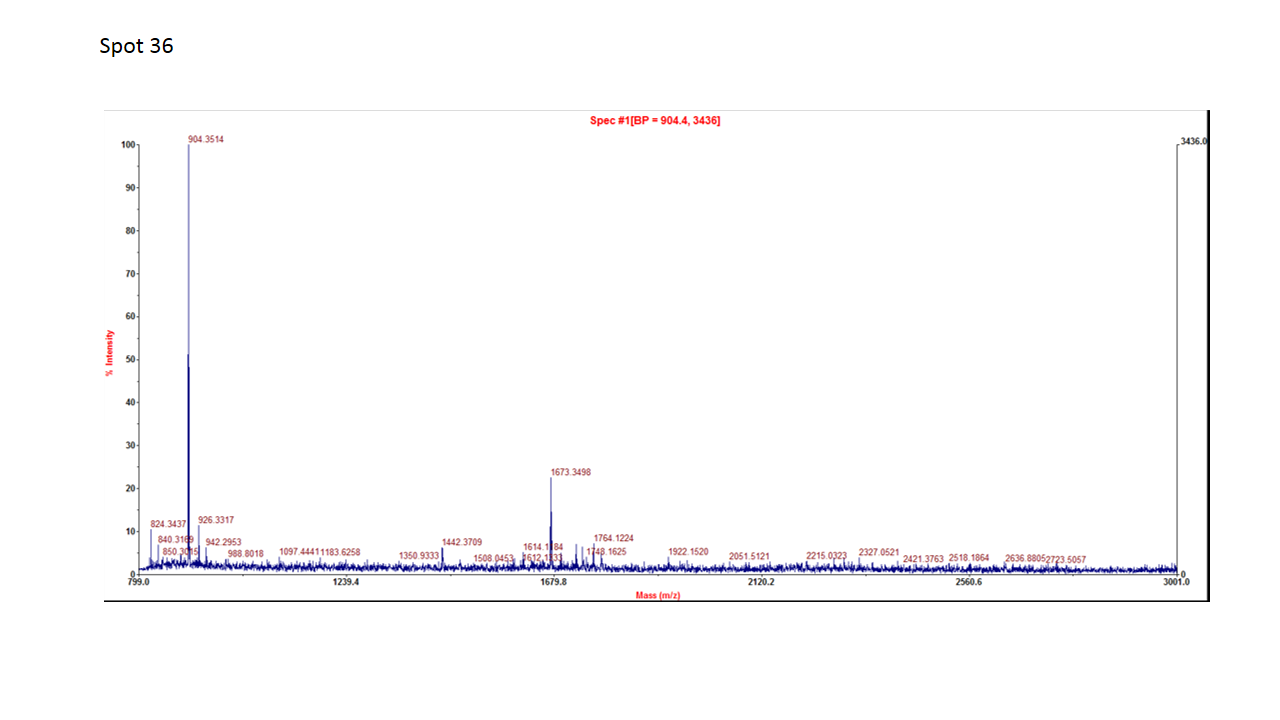

Supplement: S1 File — (ZIP) [file pone.0157439.s006.zip › Slide32.TIF]

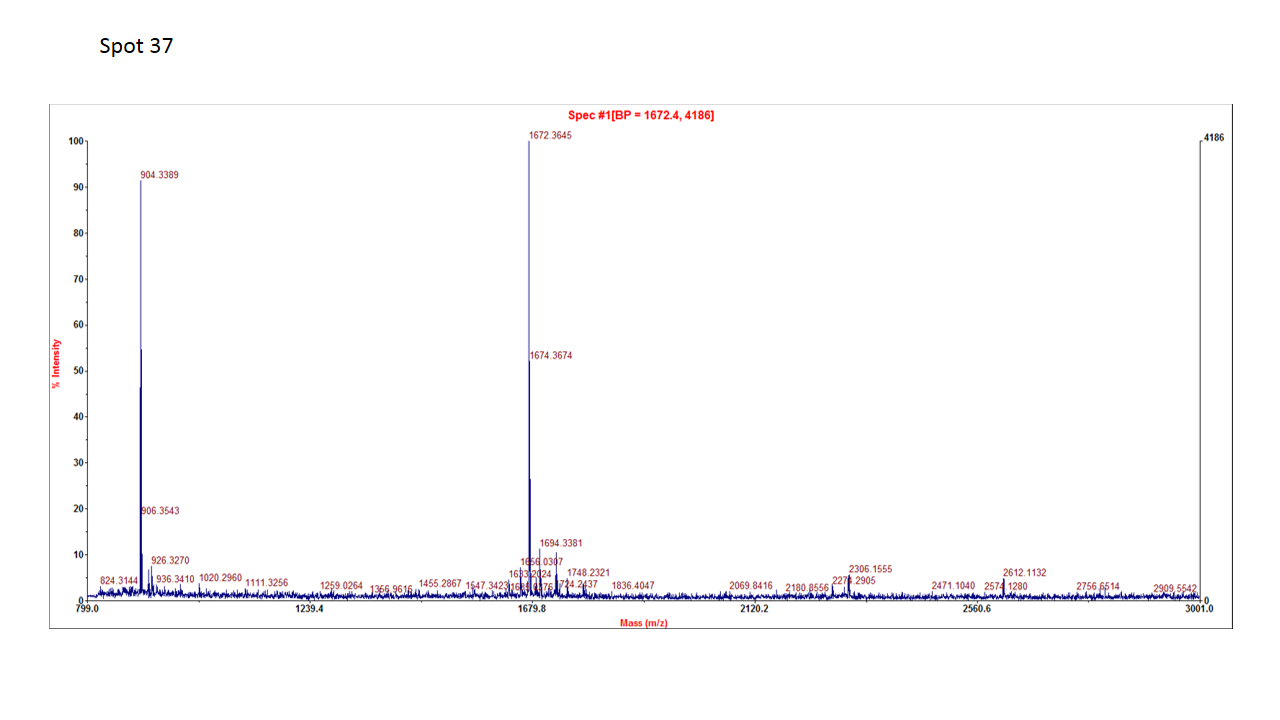

Supplement: S1 File — (ZIP) [file pone.0157439.s006.zip › Slide33.TIF]

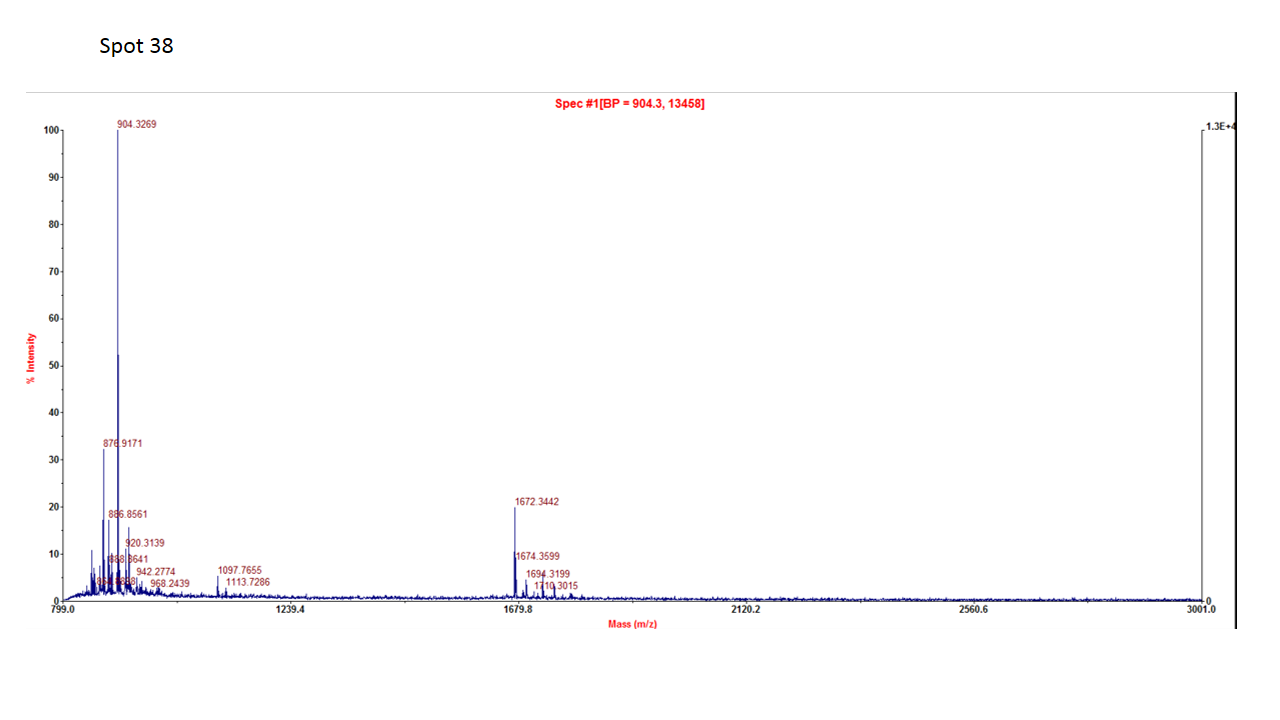

Supplement: S1 File — (ZIP) [file pone.0157439.s006.zip › Slide34.TIF]

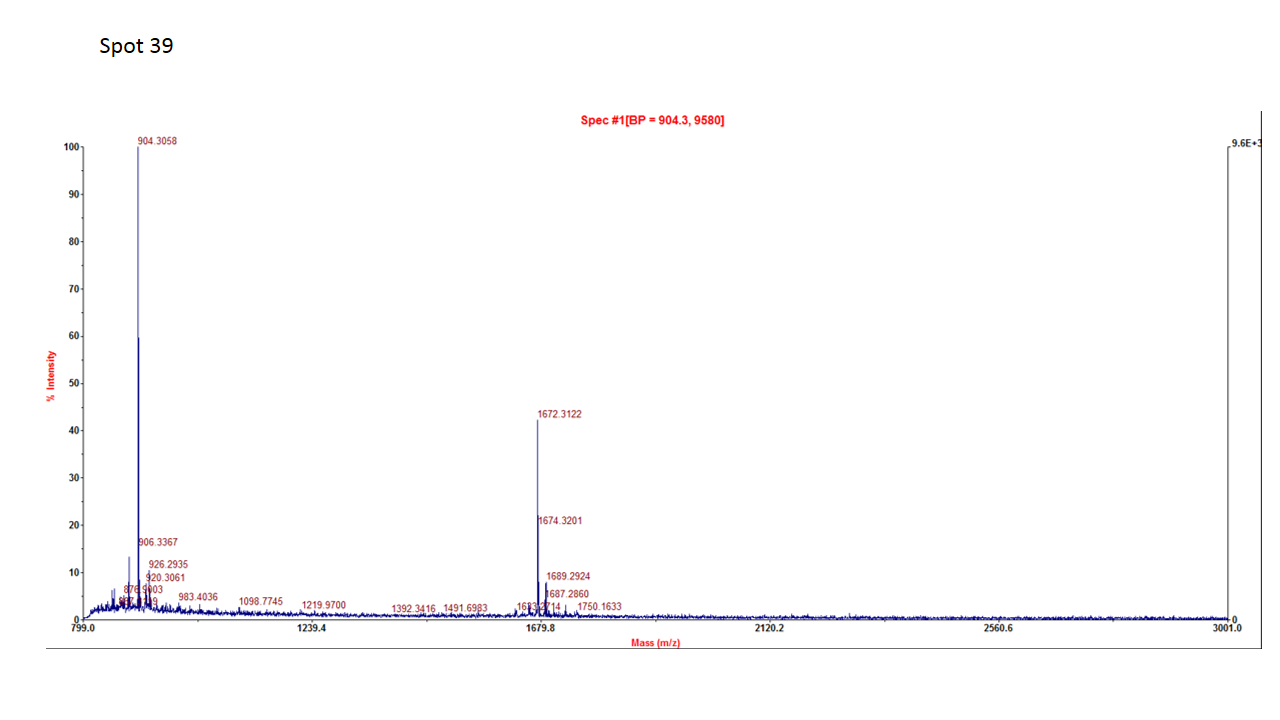

Supplement: S1 File — (ZIP) [file pone.0157439.s006.zip › Slide35.TIF]

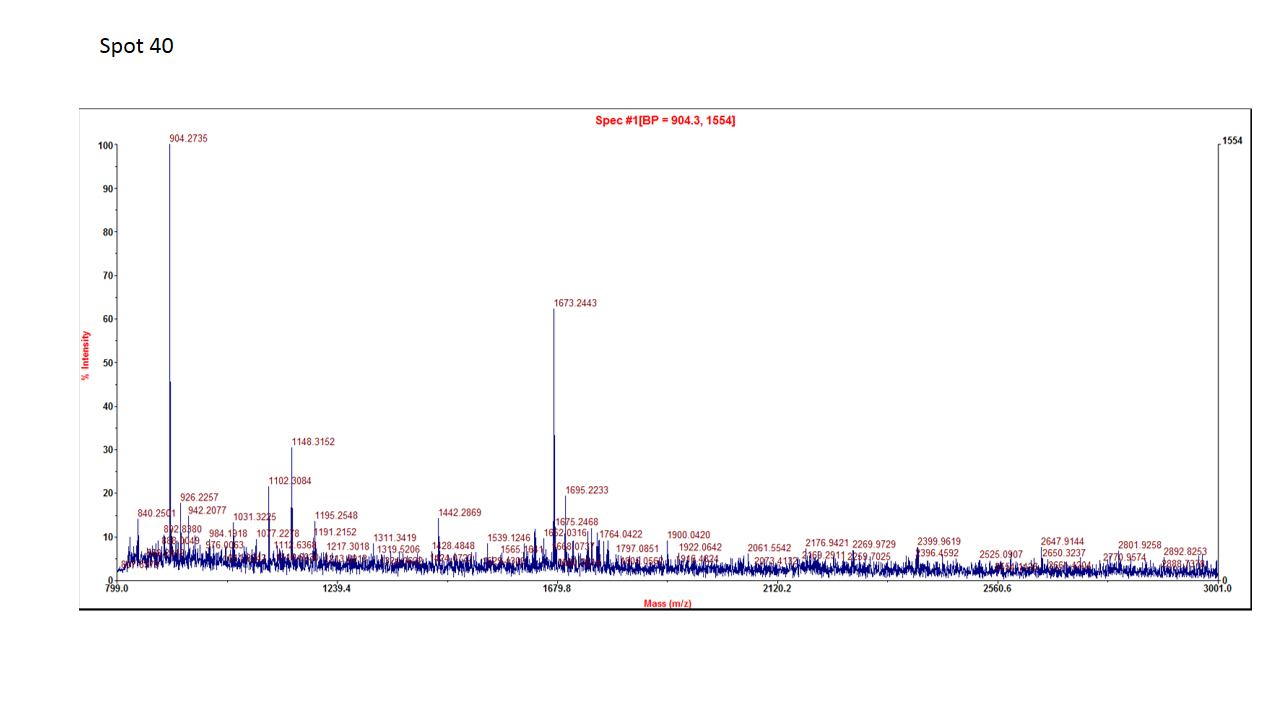

Supplement: S1 File — (ZIP) [file pone.0157439.s006.zip › Slide36.TIF]

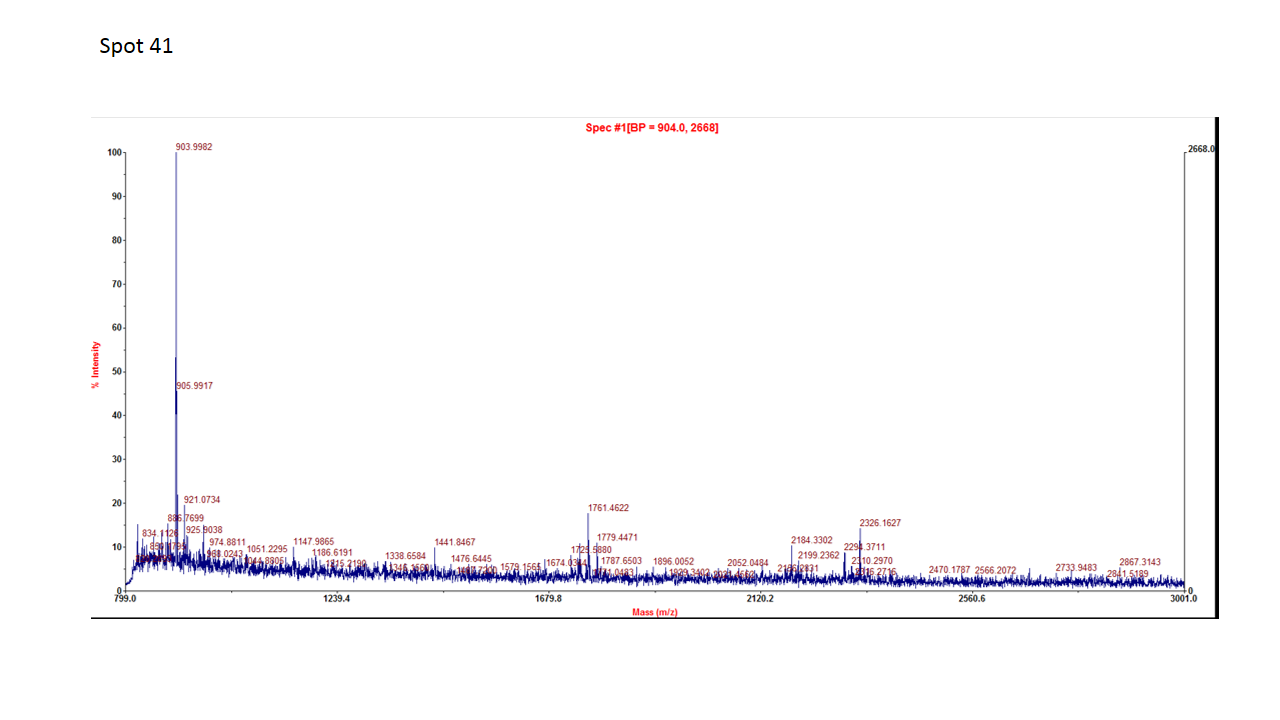

Supplement: S1 File — (ZIP) [file pone.0157439.s006.zip › Slide37.TIF]

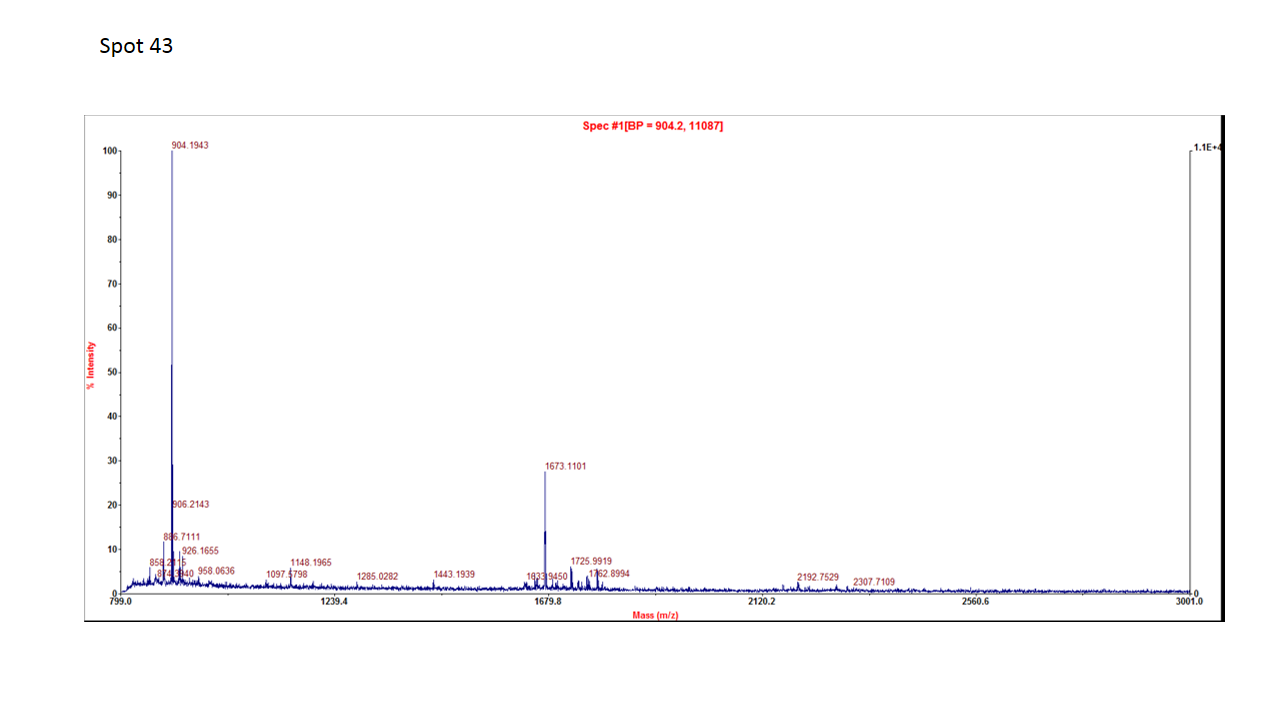

Supplement: S1 File — (ZIP) [file pone.0157439.s006.zip › Slide38.TIF]

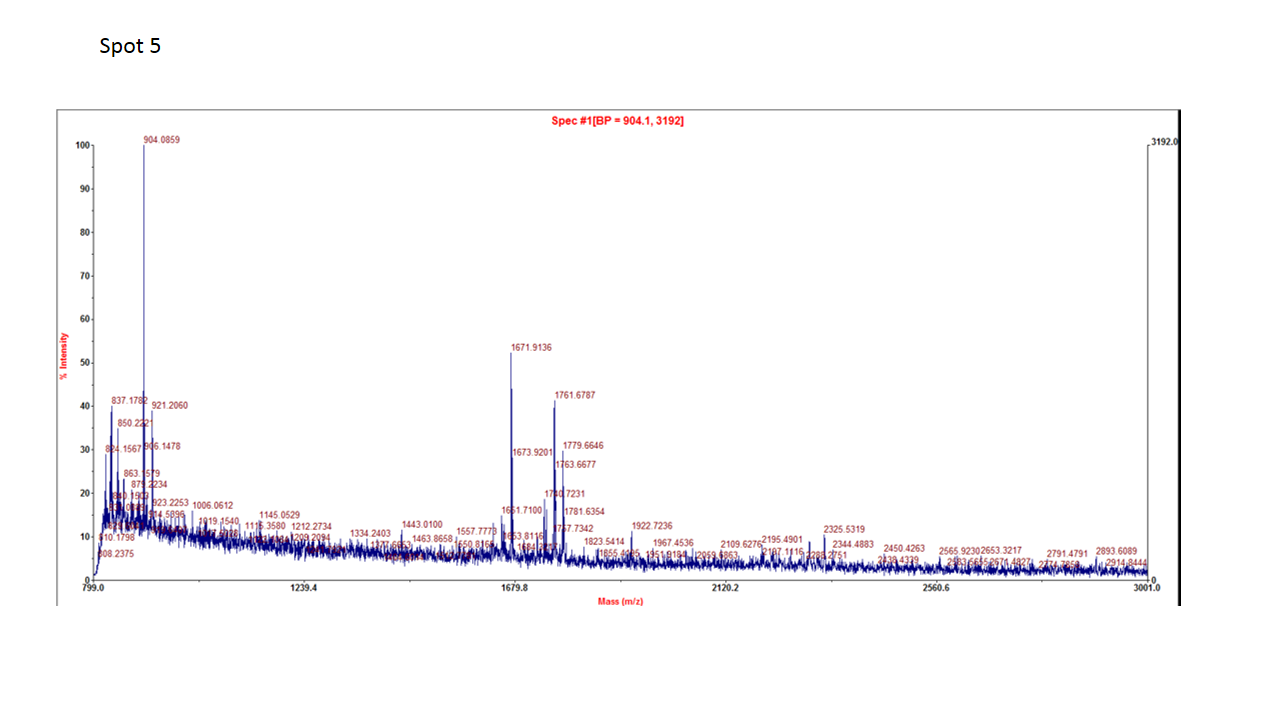

Supplement: S1 File — (ZIP) [file pone.0157439.s006.zip › Slide4.TIF]

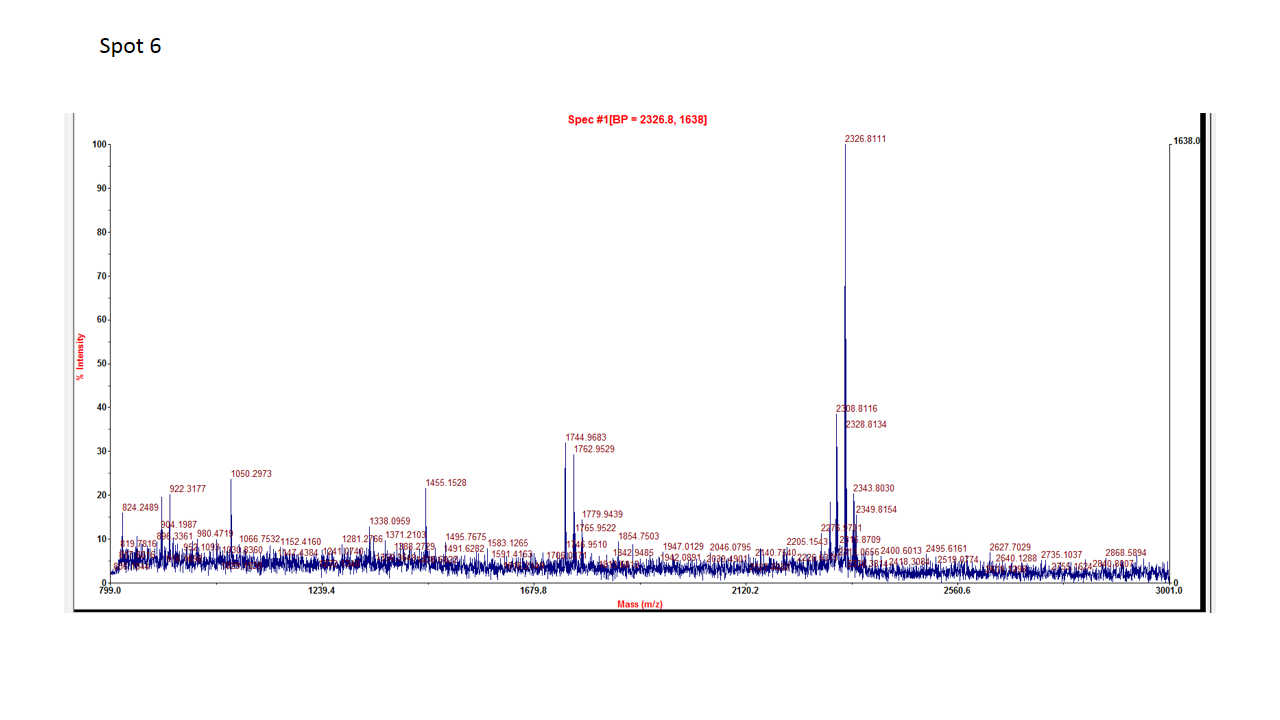

Supplement: S1 File — (ZIP) [file pone.0157439.s006.zip › Slide5.TIF]

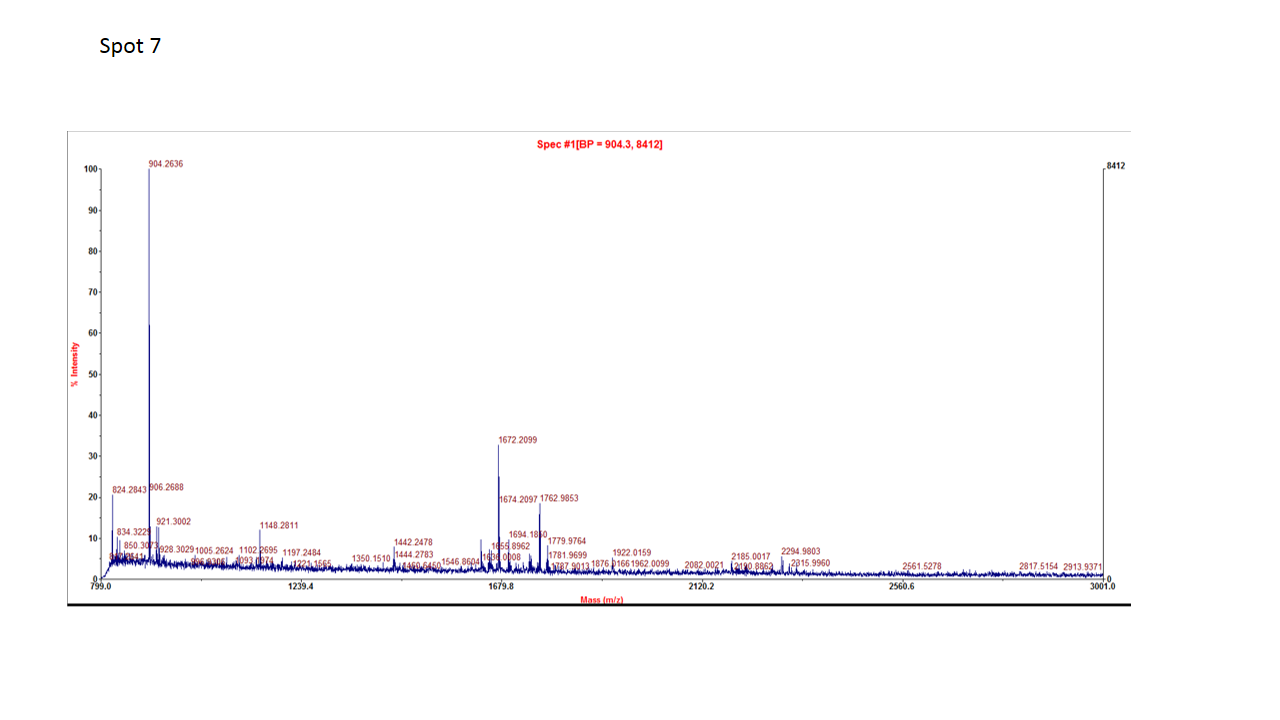

Supplement: S1 File — (ZIP) [file pone.0157439.s006.zip › Slide6.TIF]

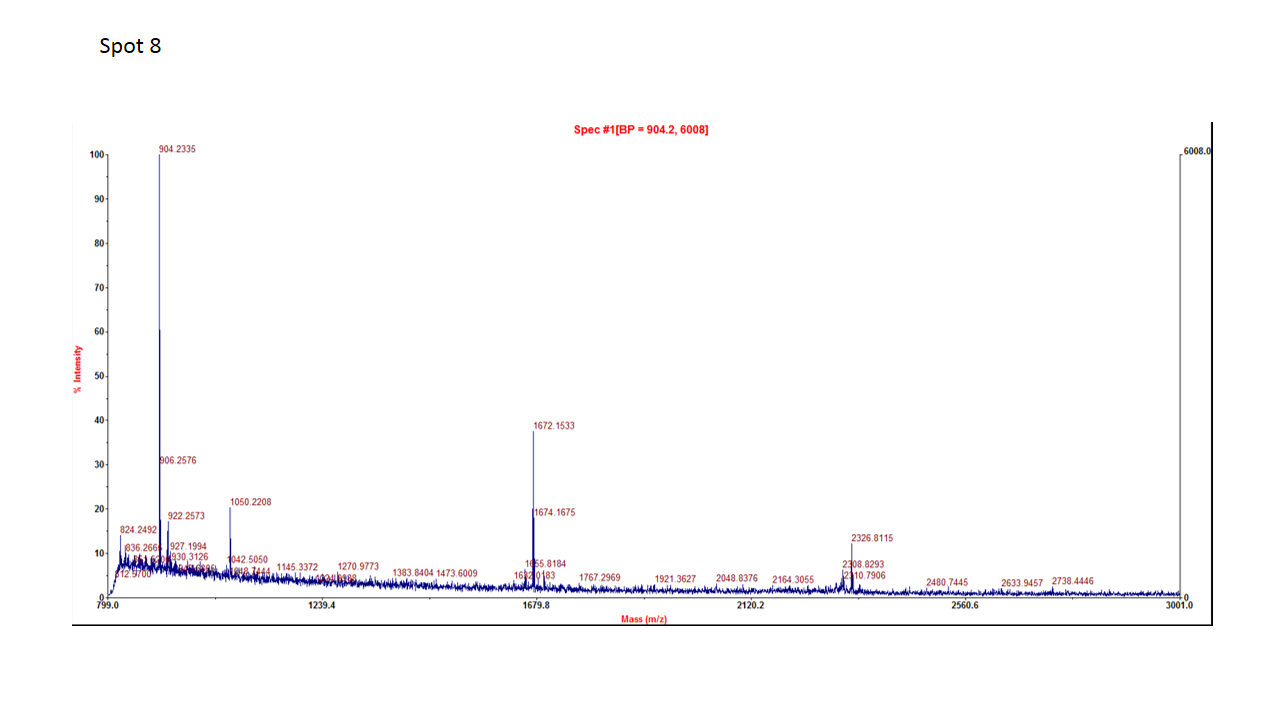

Supplement: S1 File — (ZIP) [file pone.0157439.s006.zip › Slide7.TIF]

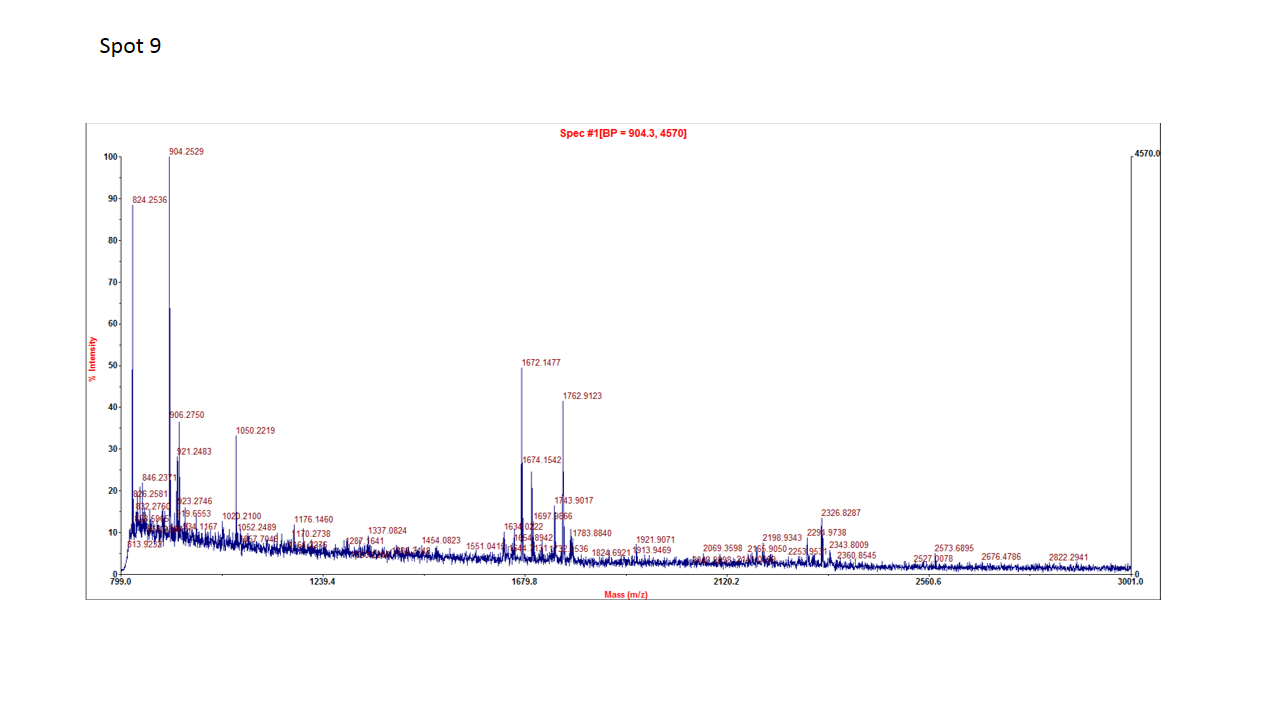

Supplement: S1 File — (ZIP) [file pone.0157439.s006.zip › Slide8.TIF]

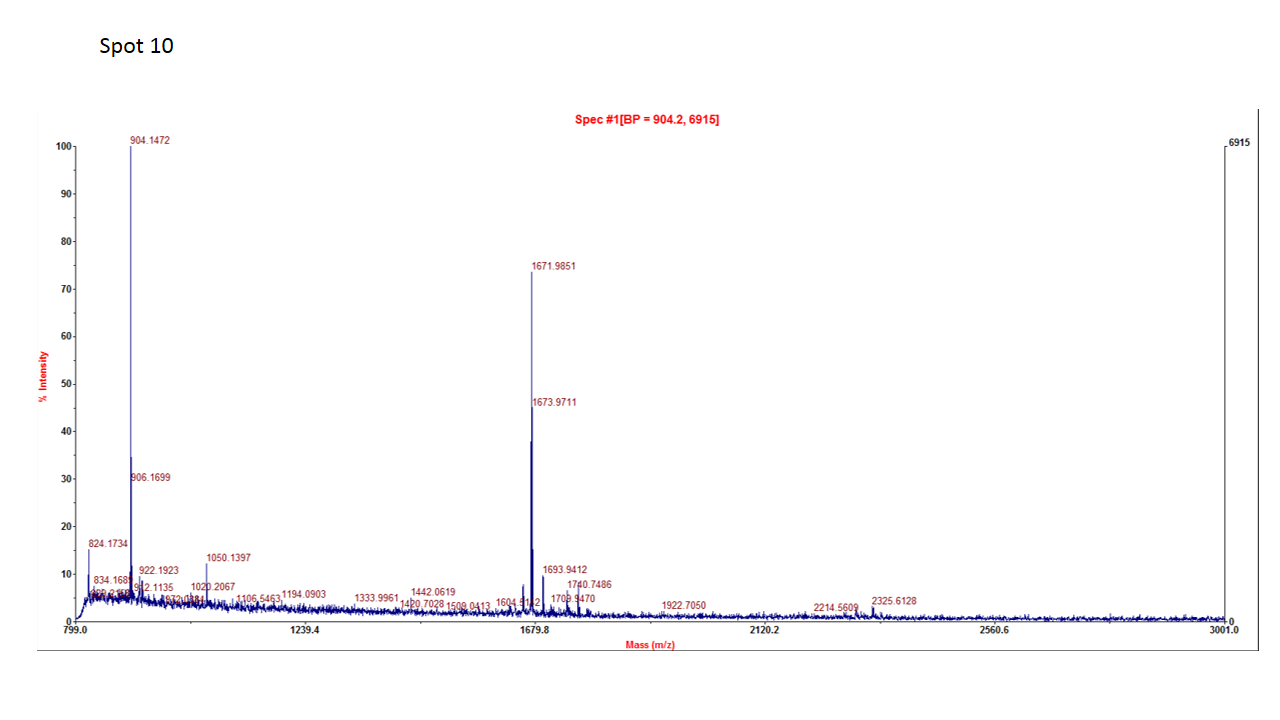

Supplement: S1 File — (ZIP) [file pone.0157439.s006.zip › Slide9.TIF]

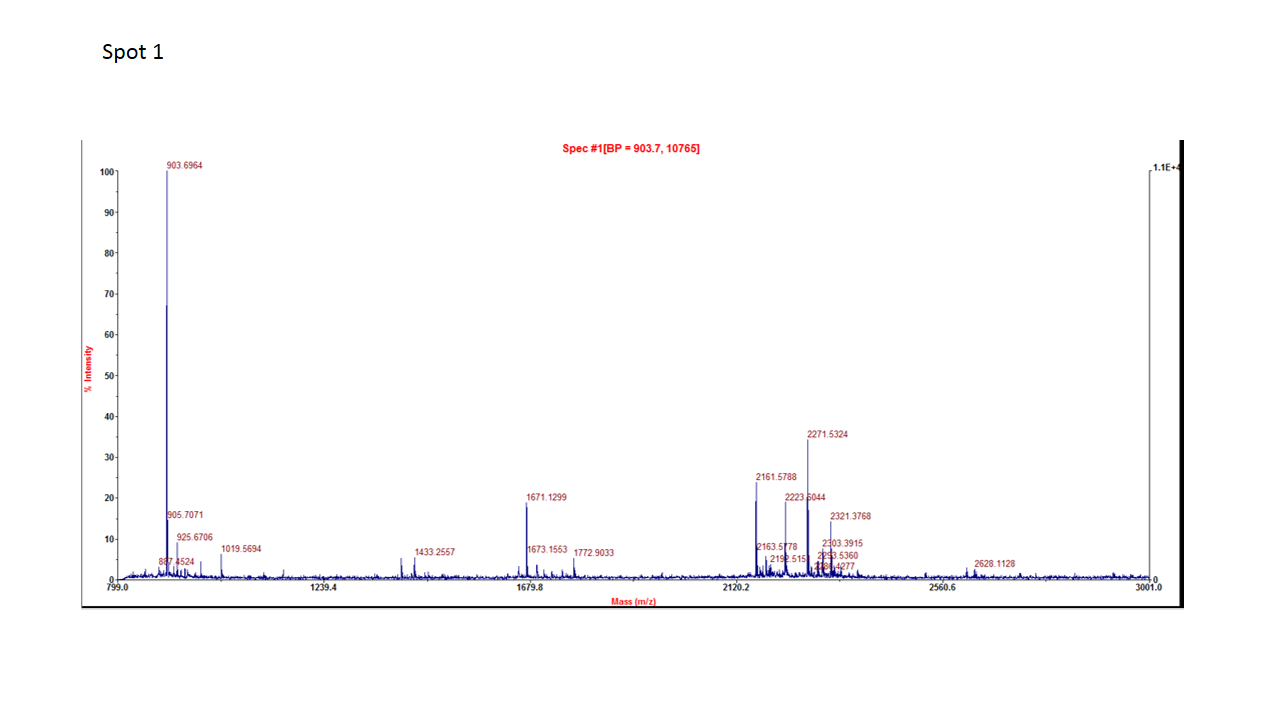

Supplement: S2 File — (ZIP) [file pone.0157439.s007.zip › Slide1.TIF]

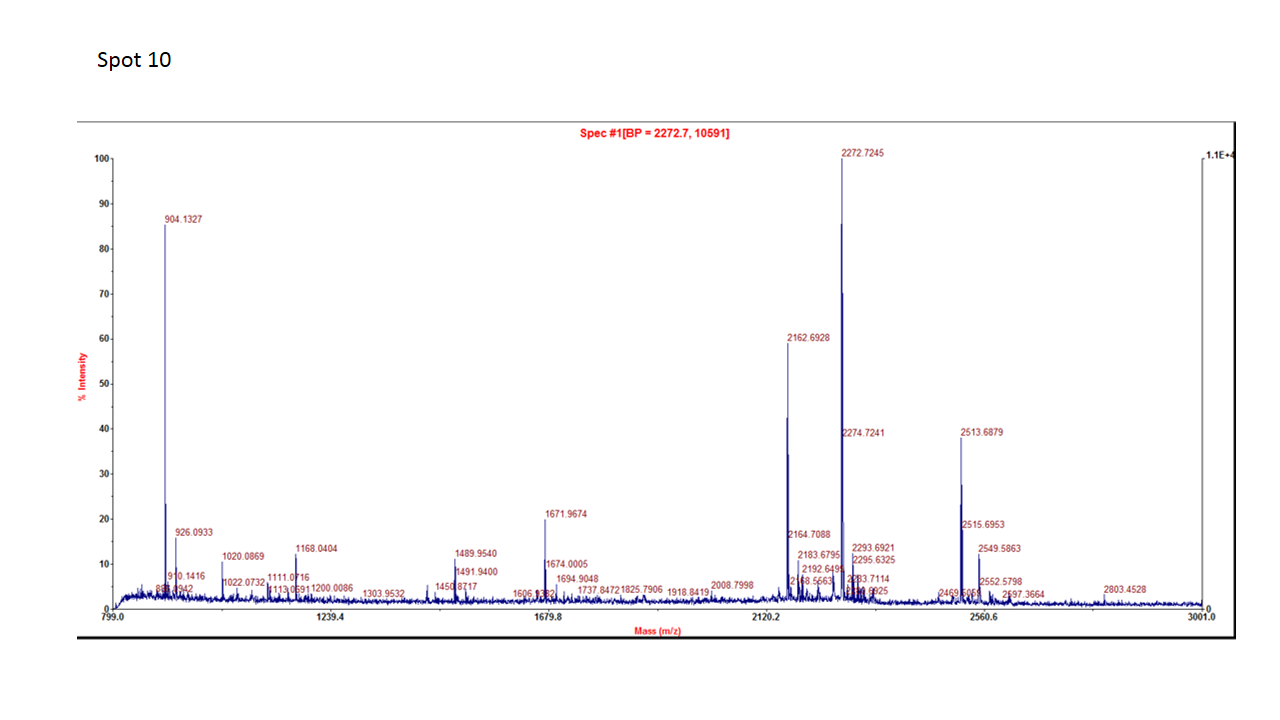

Supplement: S2 File — (ZIP) [file pone.0157439.s007.zip › Slide10.TIF]

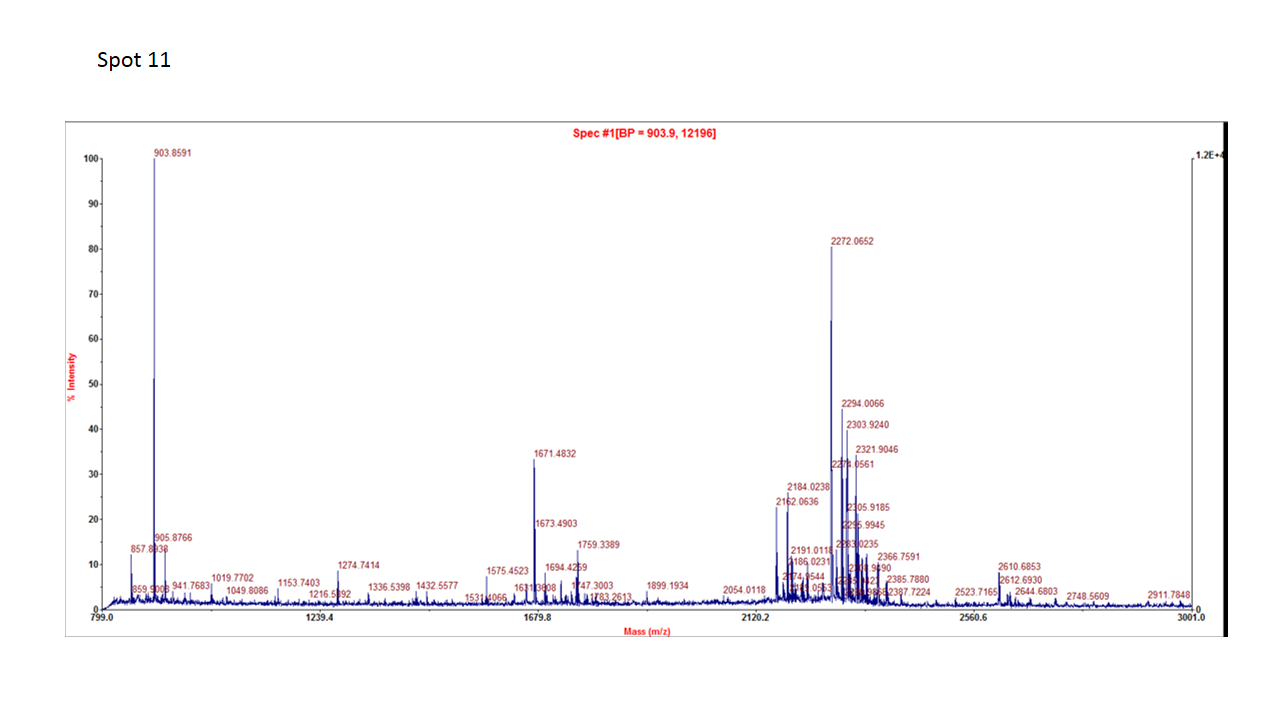

Supplement: S2 File — (ZIP) [file pone.0157439.s007.zip › Slide11.TIF]

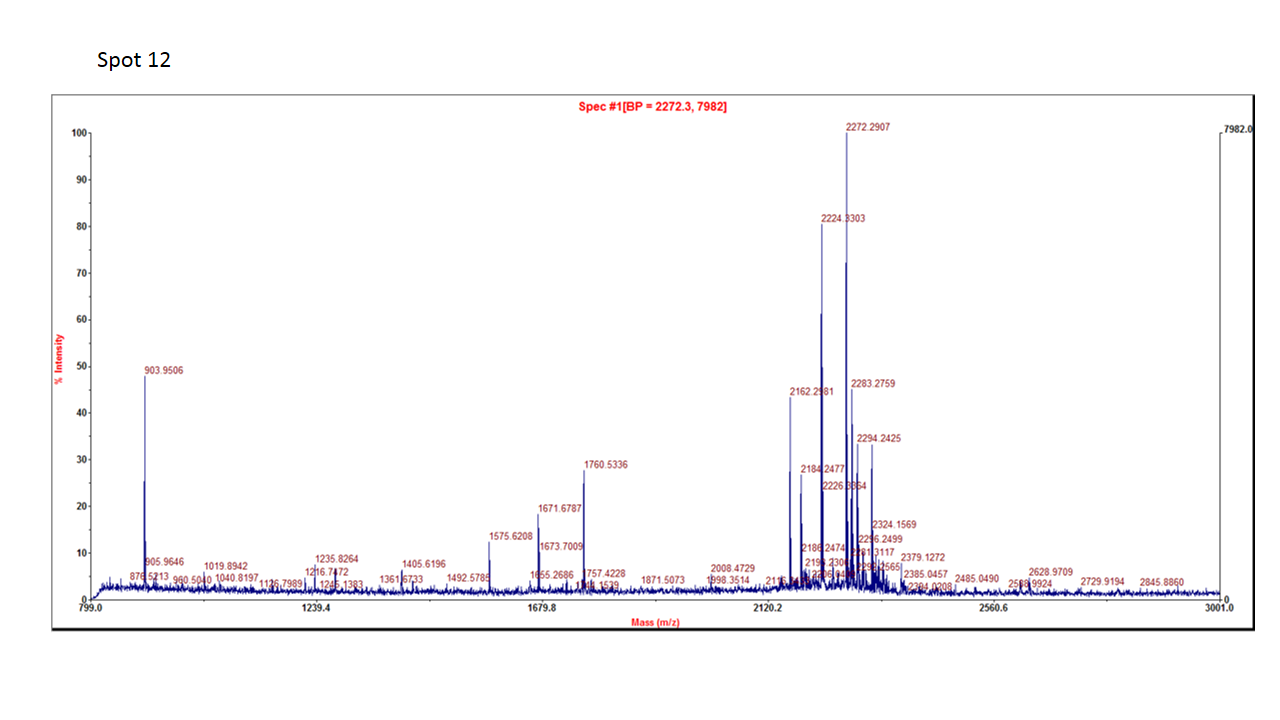

Supplement: S2 File — (ZIP) [file pone.0157439.s007.zip › Slide12.TIF]

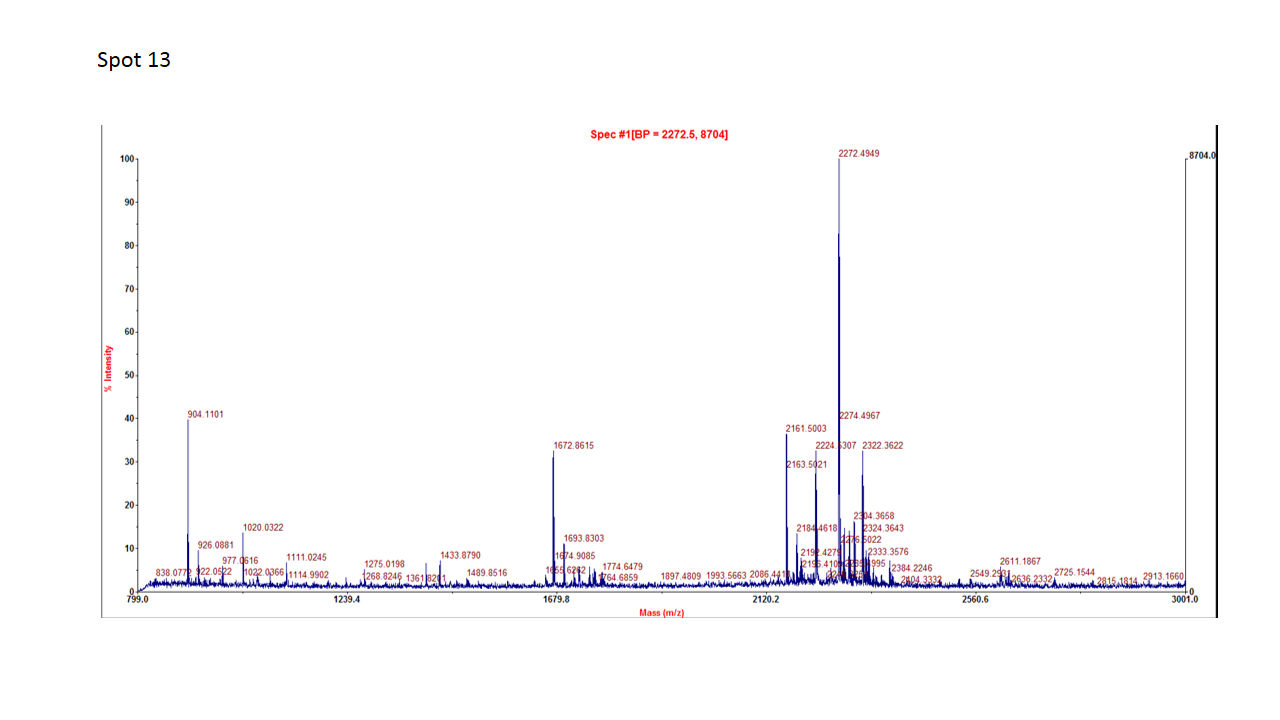

Supplement: S2 File — (ZIP) [file pone.0157439.s007.zip › Slide13.TIF]

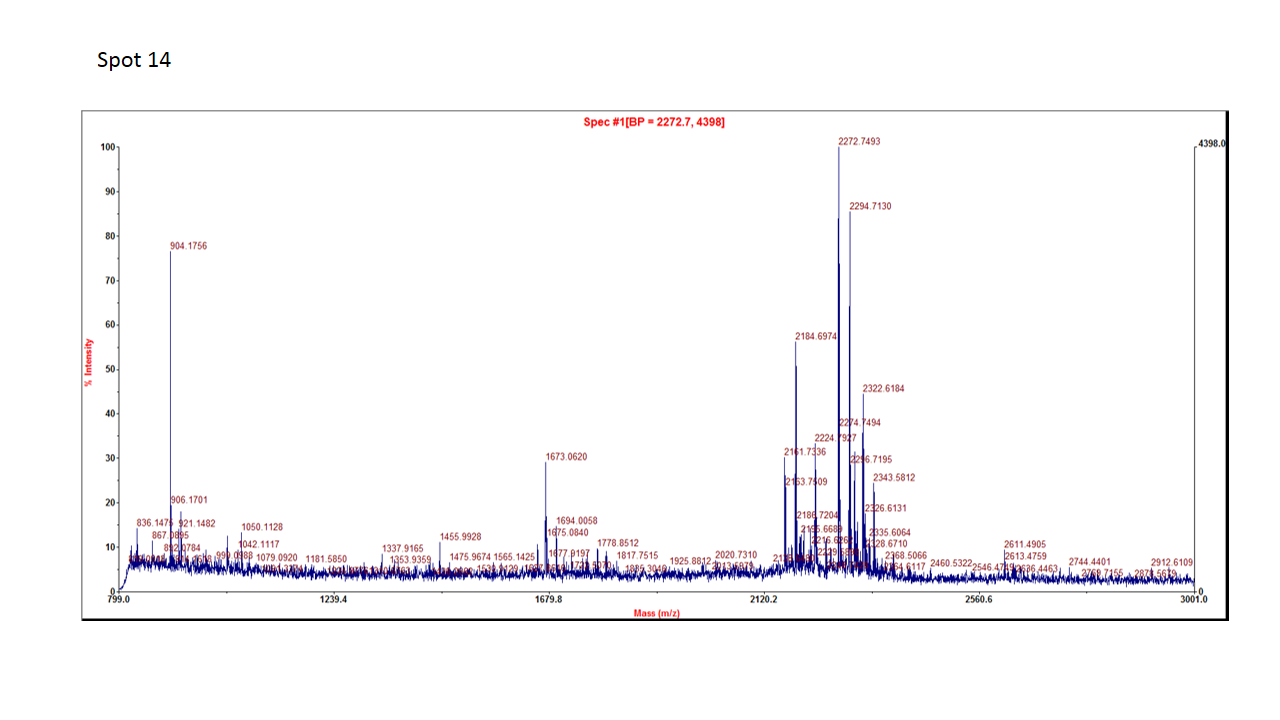

Supplement: S2 File — (ZIP) [file pone.0157439.s007.zip › Slide14.TIF]

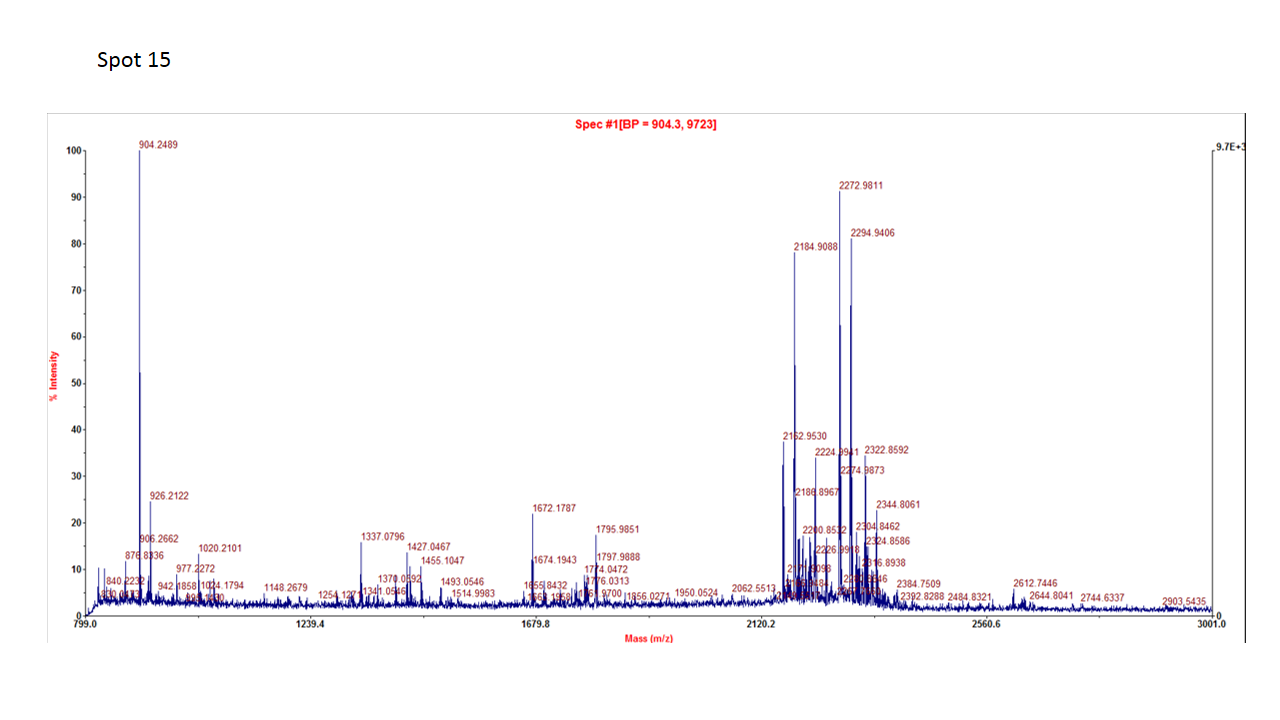

Supplement: S2 File — (ZIP) [file pone.0157439.s007.zip › Slide15.TIF]

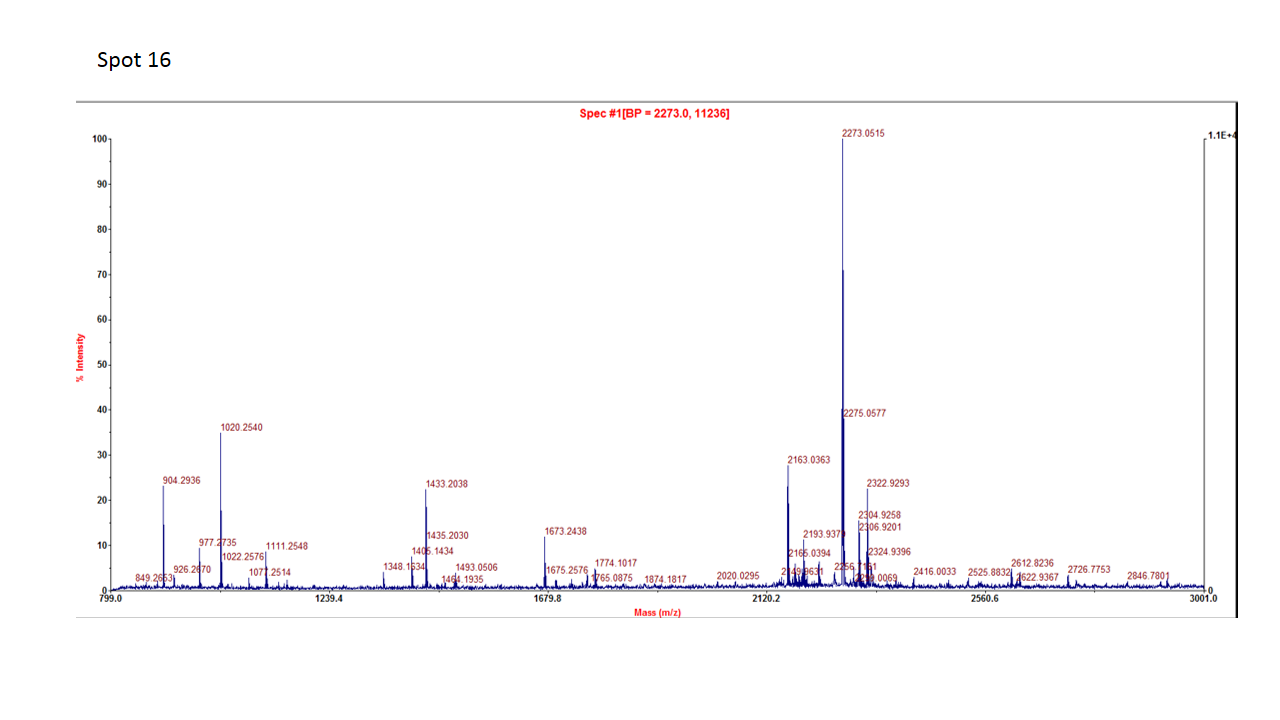

Supplement: S2 File — (ZIP) [file pone.0157439.s007.zip › Slide16.TIF]

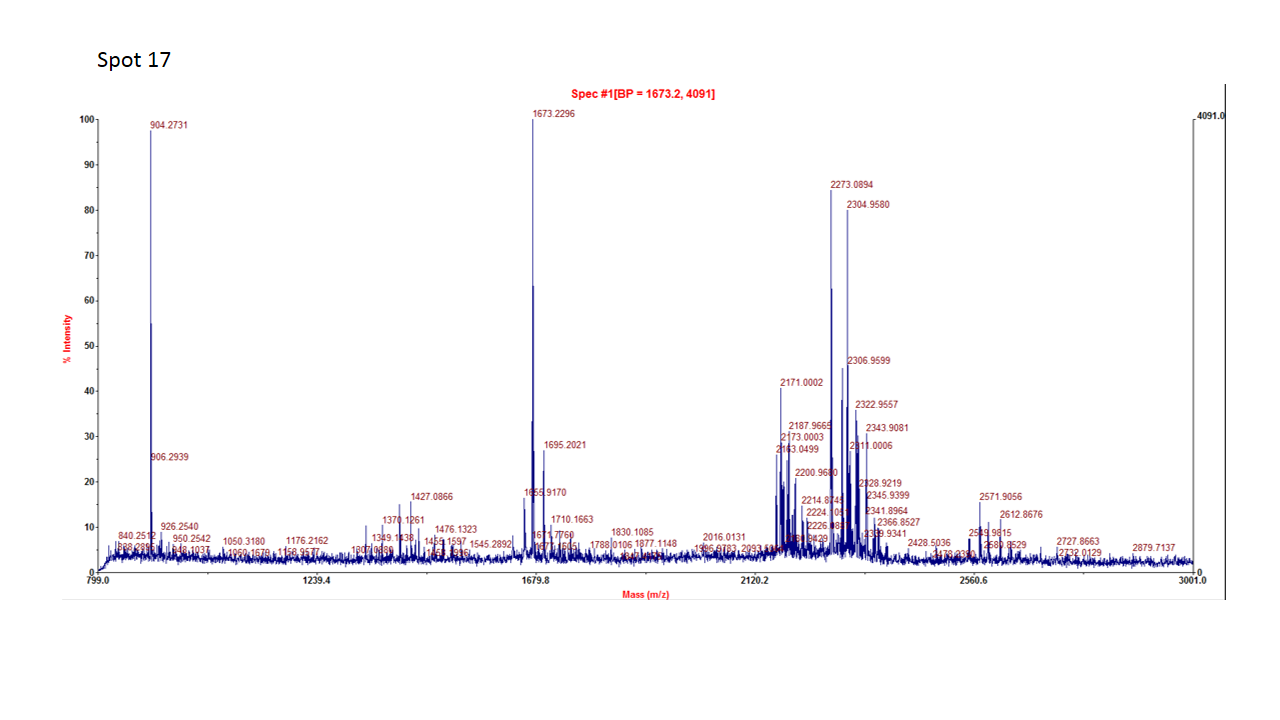

Supplement: S2 File — (ZIP) [file pone.0157439.s007.zip › Slide17.TIF]

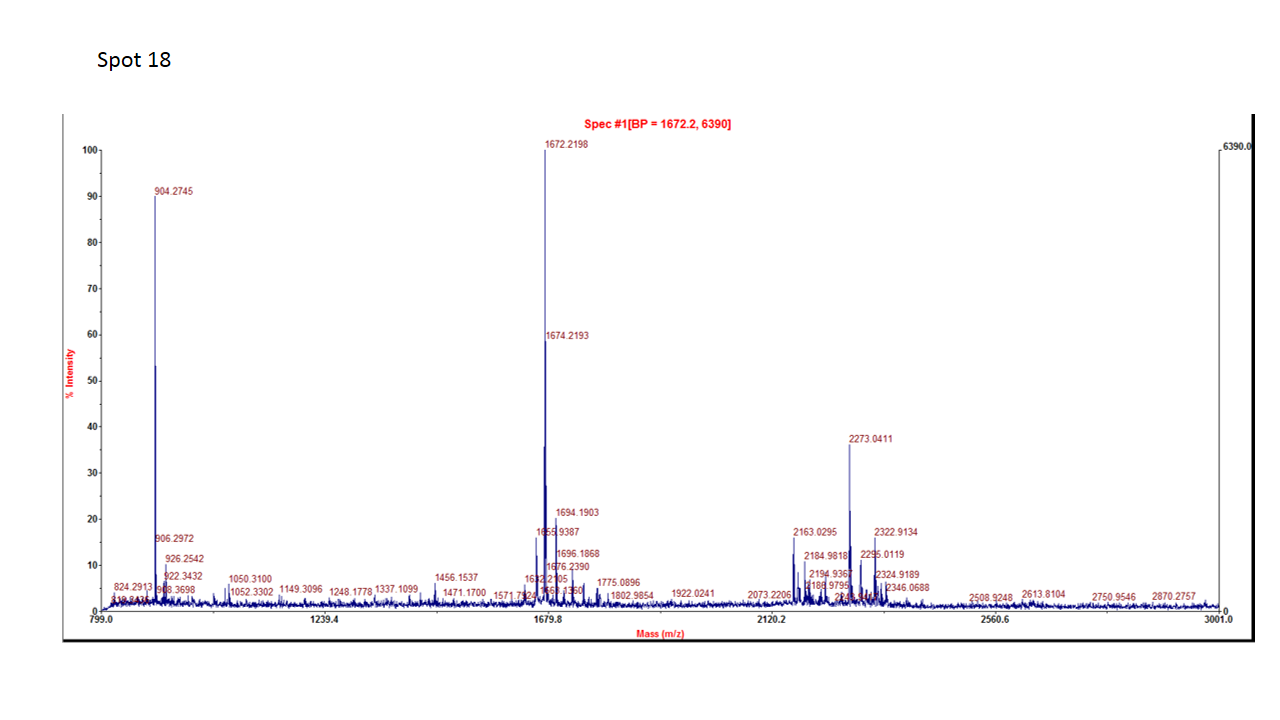

Supplement: S2 File — (ZIP) [file pone.0157439.s007.zip › Slide18.TIF]

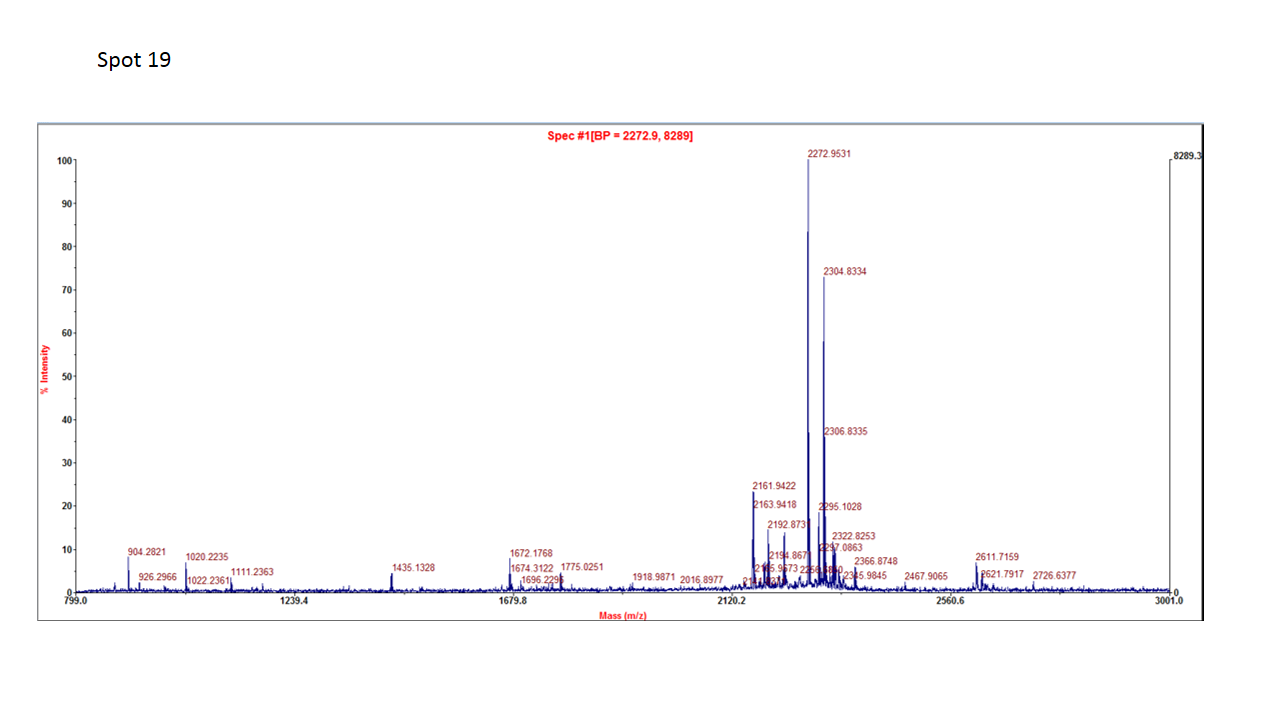

Supplement: S2 File — (ZIP) [file pone.0157439.s007.zip › Slide19.TIF]

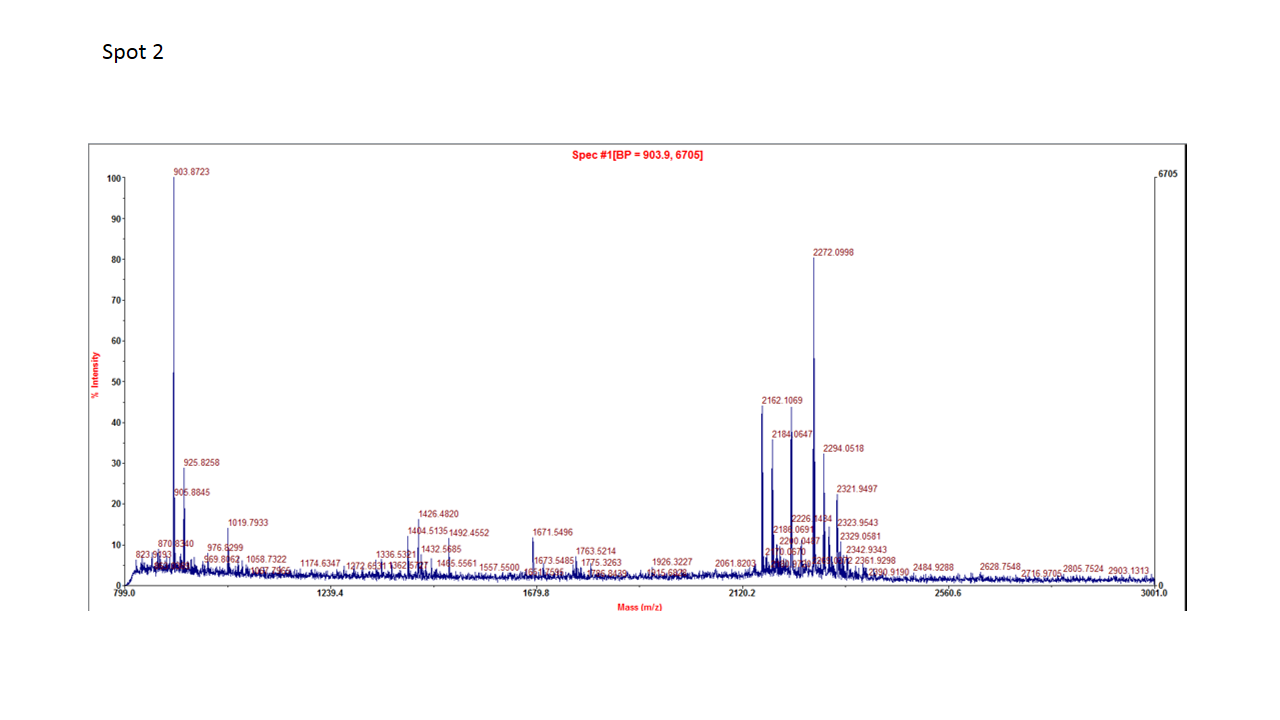

Supplement: S2 File — (ZIP) [file pone.0157439.s007.zip › Slide2.TIF]

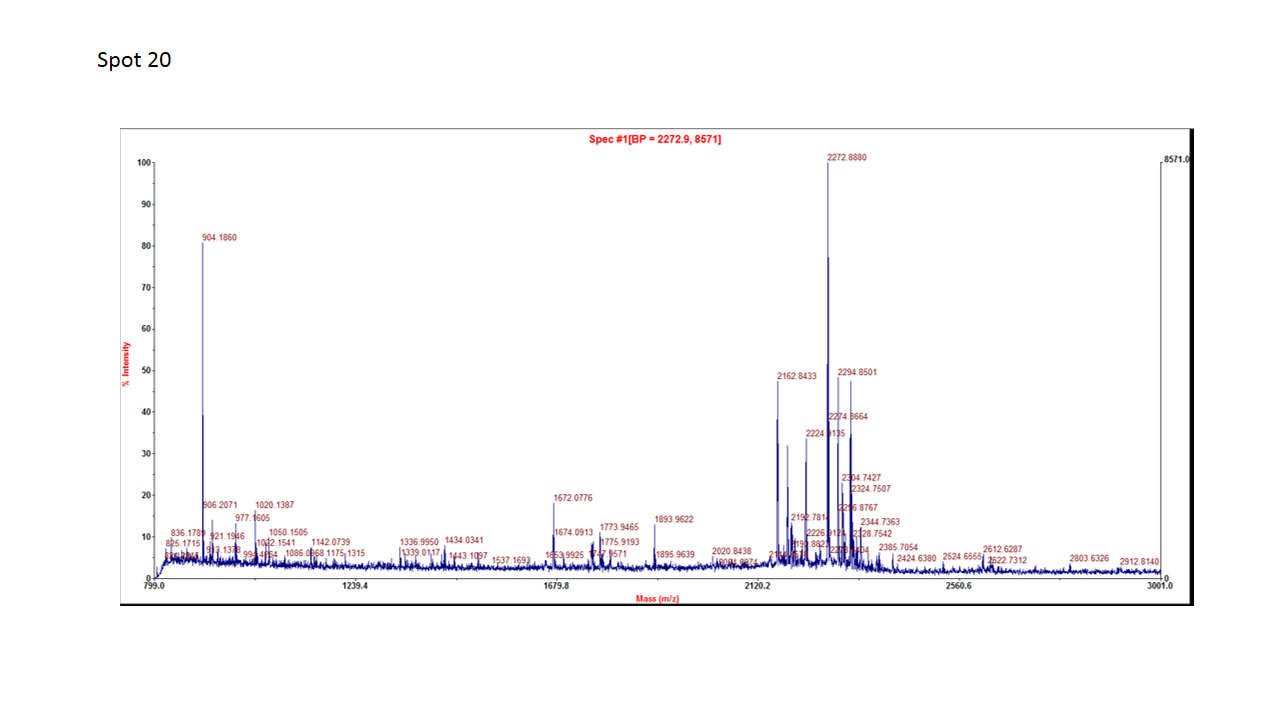

Supplement: S2 File — (ZIP) [file pone.0157439.s007.zip › Slide20.TIF]

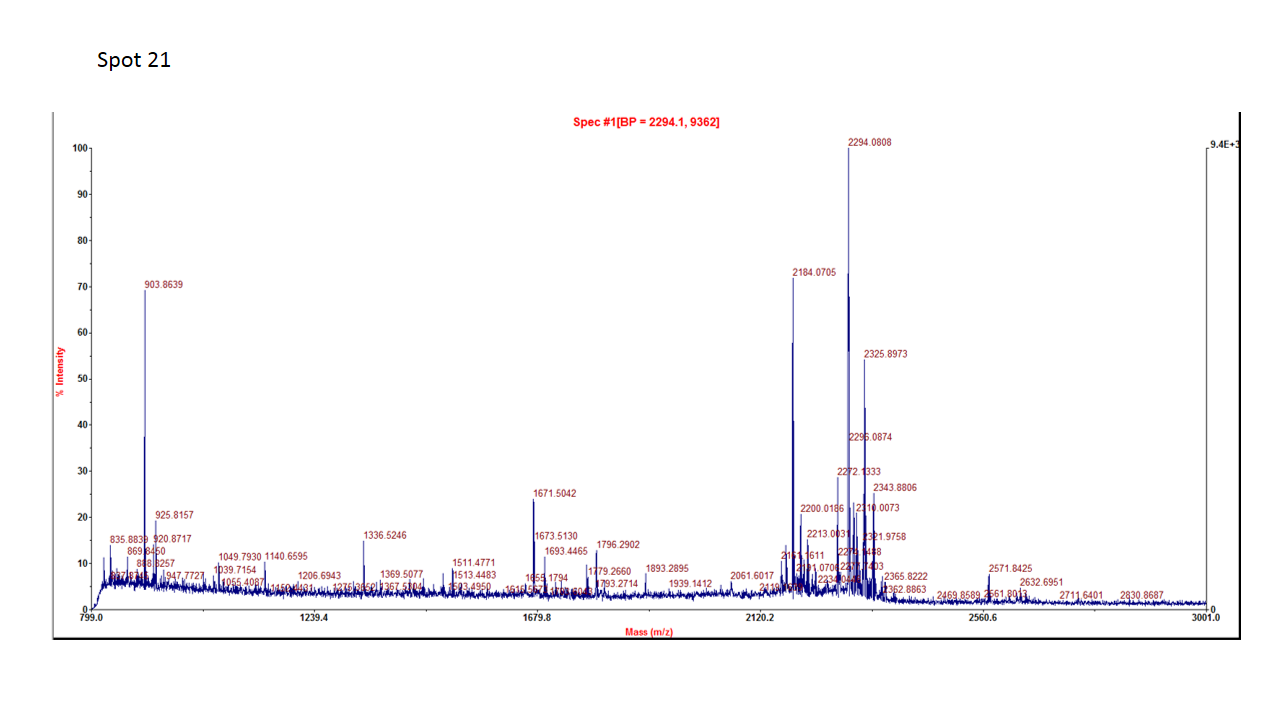

Supplement: S2 File — (ZIP) [file pone.0157439.s007.zip › Slide21.TIF]

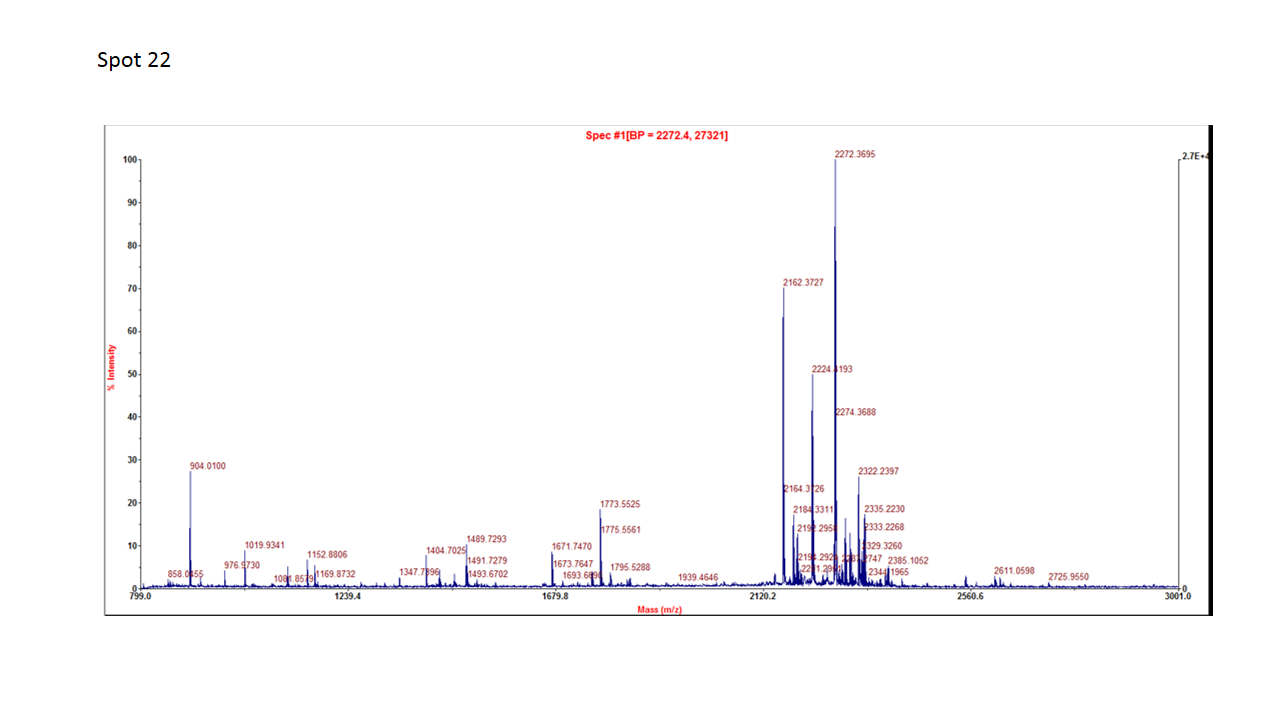

Supplement: S2 File — (ZIP) [file pone.0157439.s007.zip › Slide22.TIF]

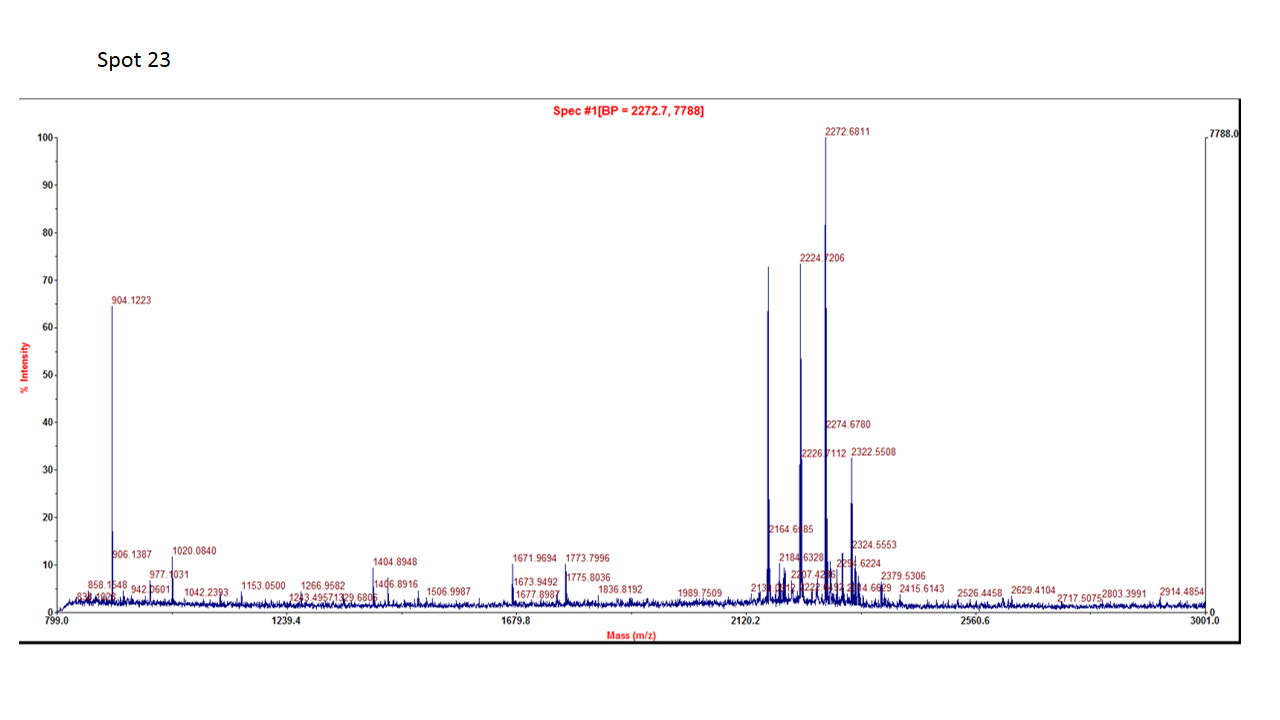

Supplement: S2 File — (ZIP) [file pone.0157439.s007.zip › Slide23.TIF]

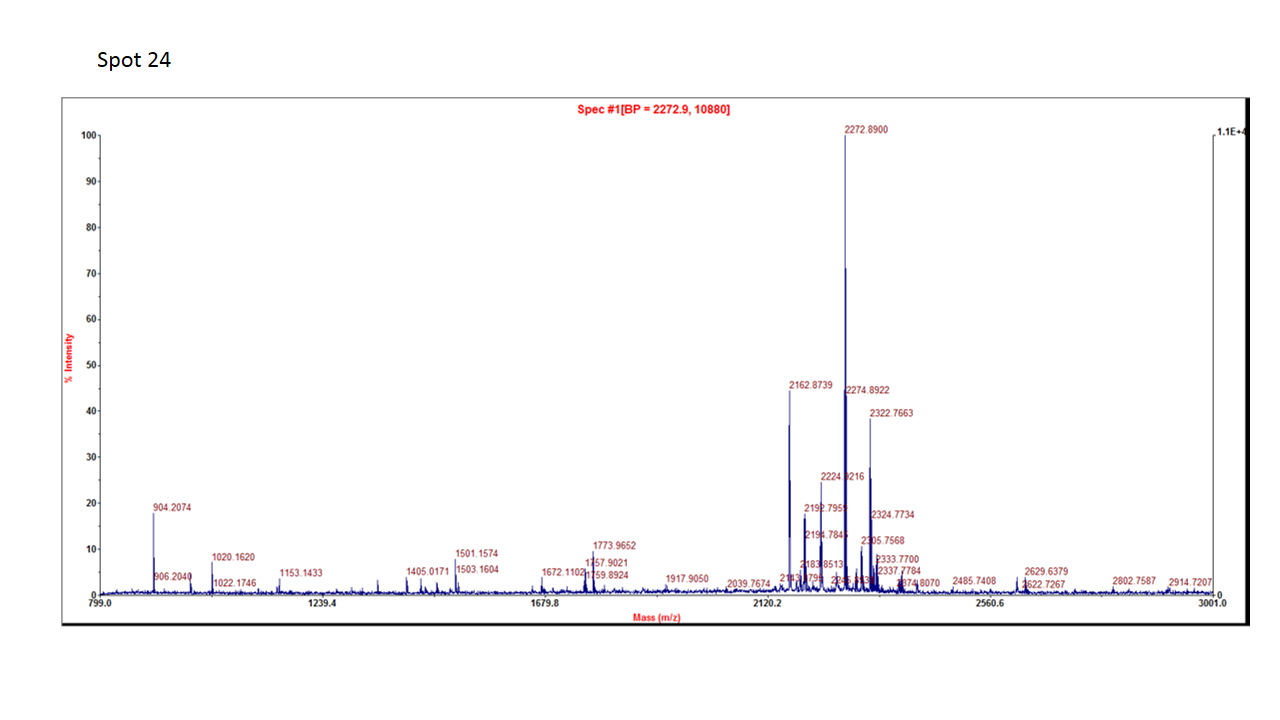

Supplement: S2 File — (ZIP) [file pone.0157439.s007.zip › Slide24.TIF]

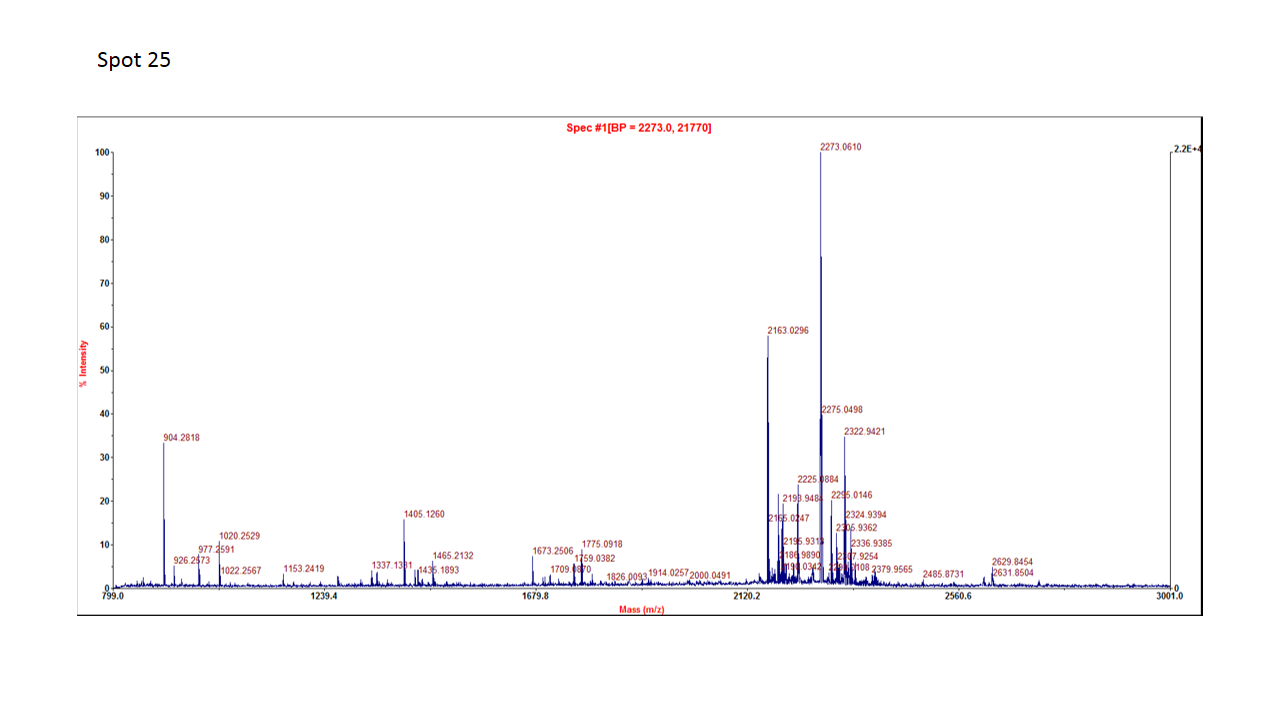

Supplement: S2 File — (ZIP) [file pone.0157439.s007.zip › Slide25.TIF]

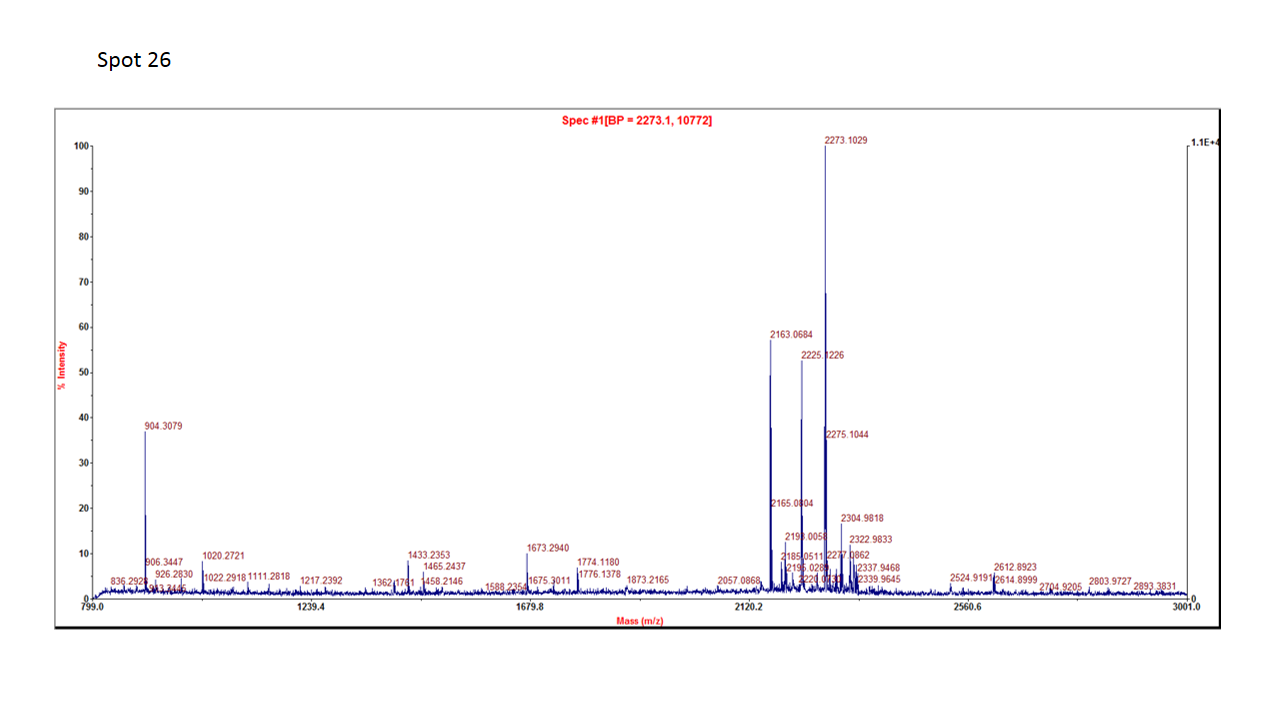

Supplement: S2 File — (ZIP) [file pone.0157439.s007.zip › Slide26.TIF]

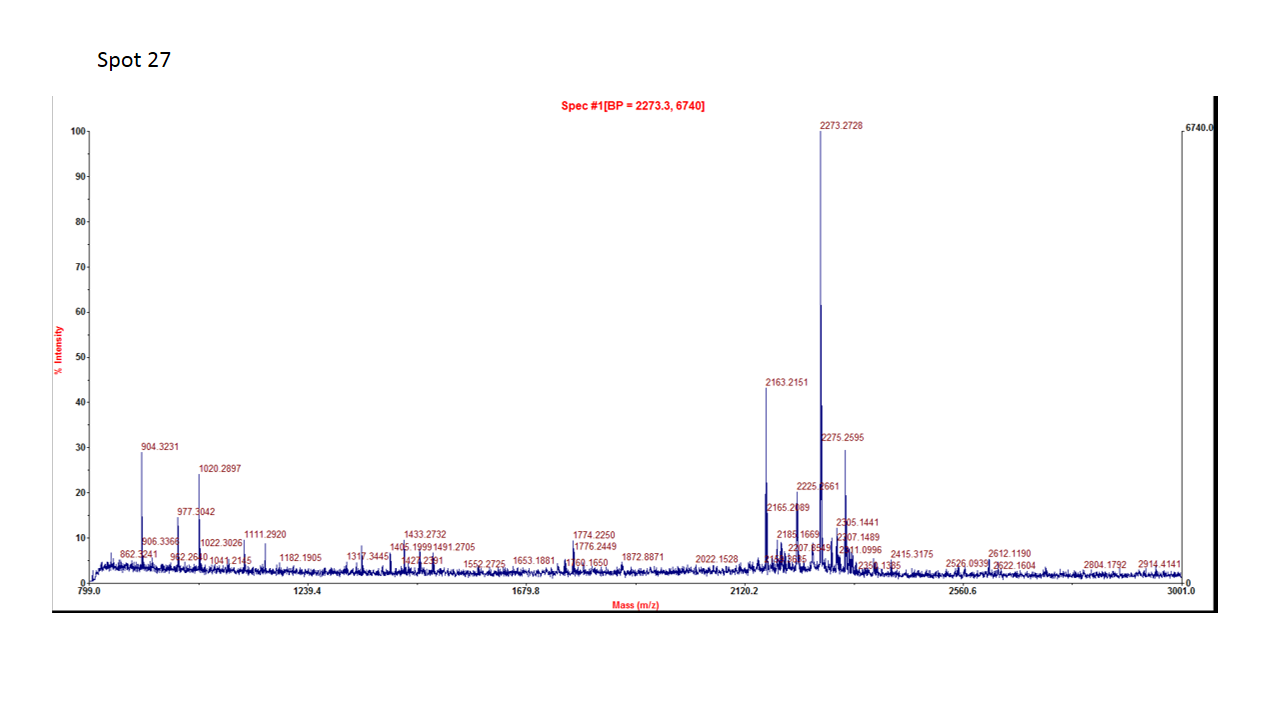

Supplement: S2 File — (ZIP) [file pone.0157439.s007.zip › Slide27.TIF]

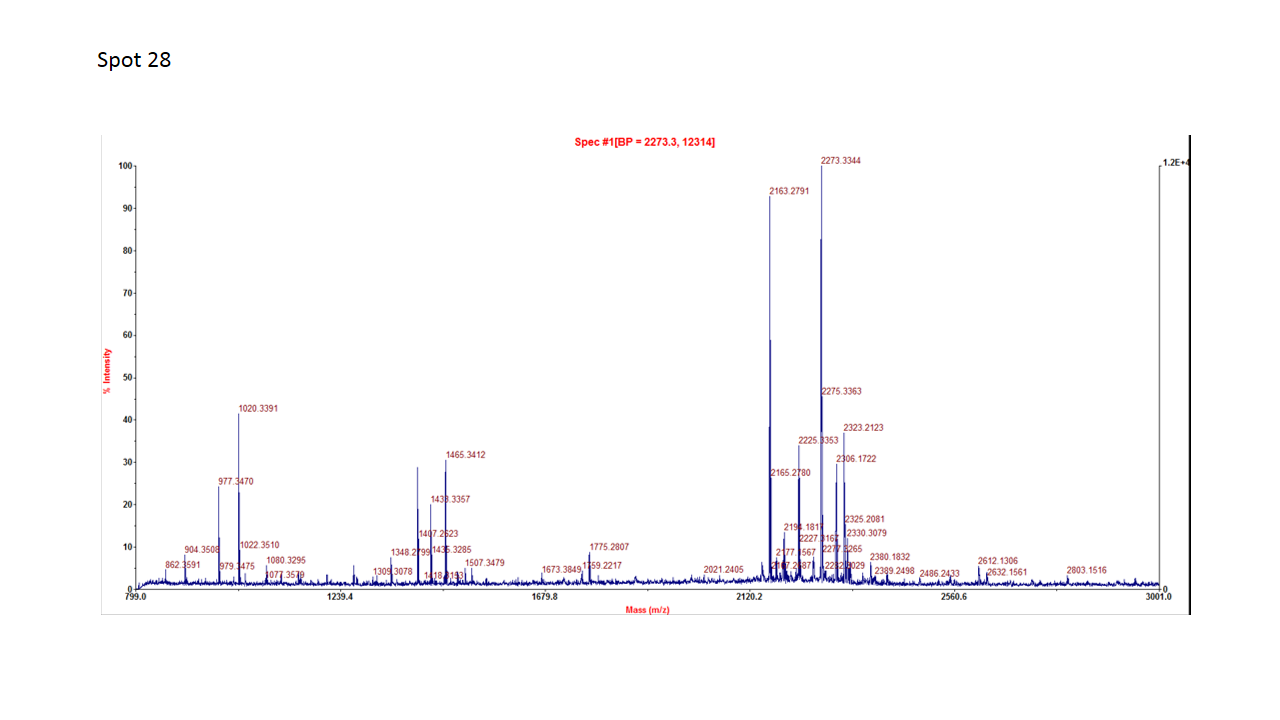

Supplement: S2 File — (ZIP) [file pone.0157439.s007.zip › Slide28.TIF]

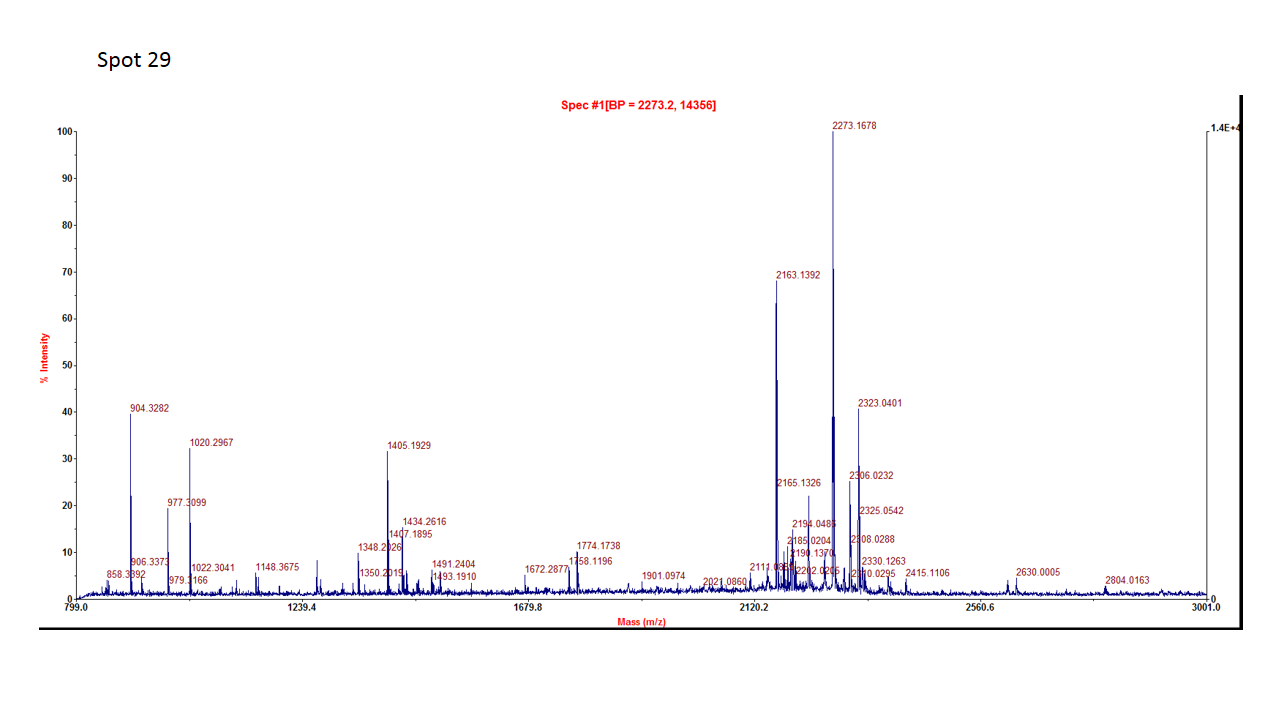

Supplement: S2 File — (ZIP) [file pone.0157439.s007.zip › Slide29.TIF]

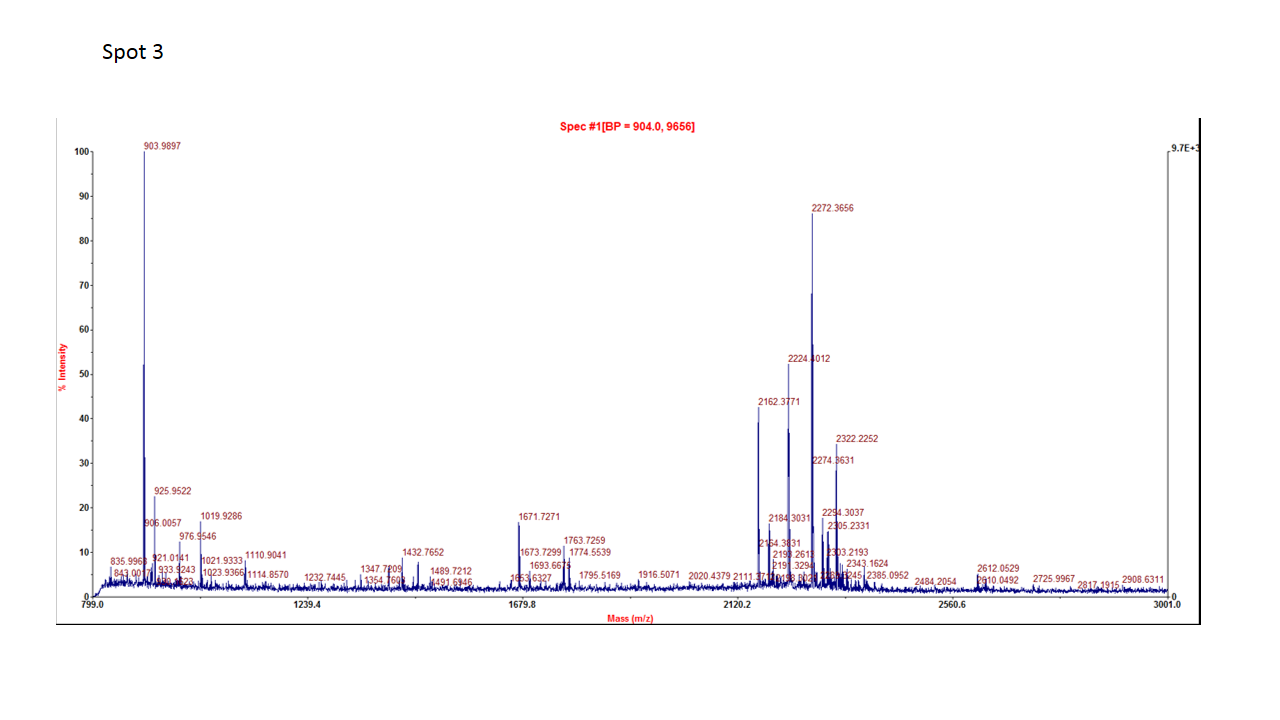

Supplement: S2 File — (ZIP) [file pone.0157439.s007.zip › Slide3.TIF]

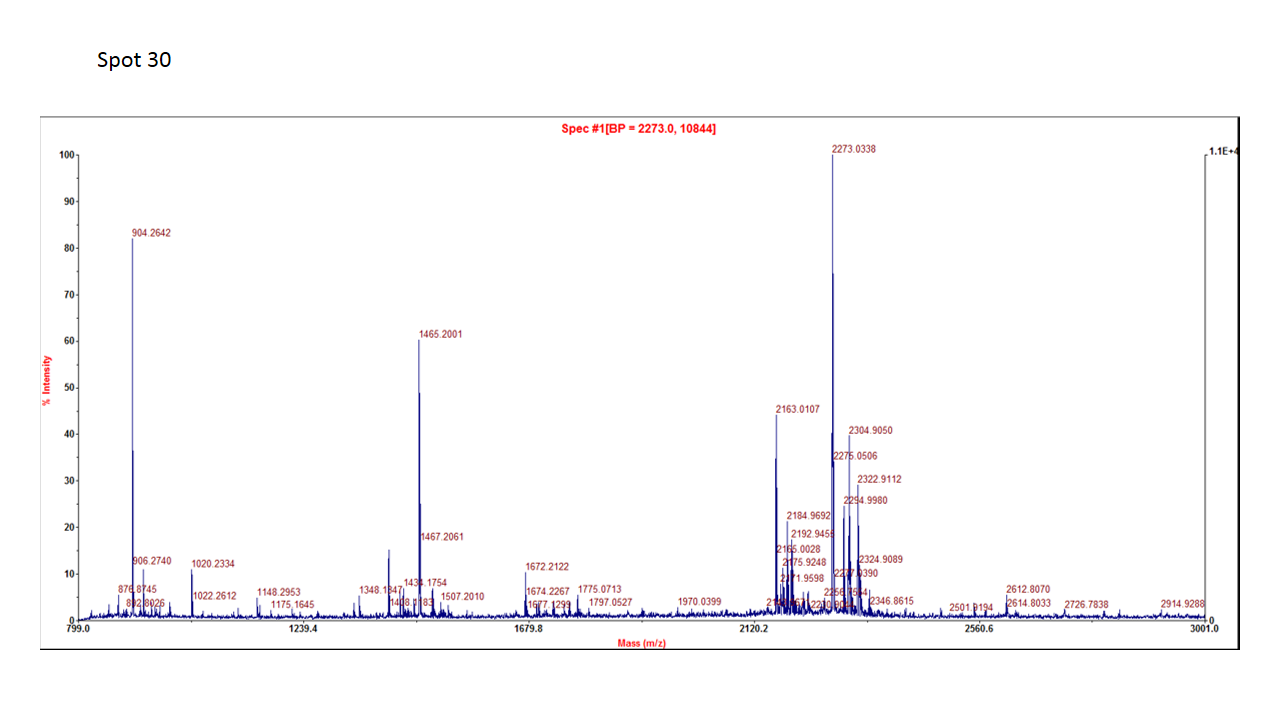

Supplement: S2 File — (ZIP) [file pone.0157439.s007.zip › Slide30.TIF]

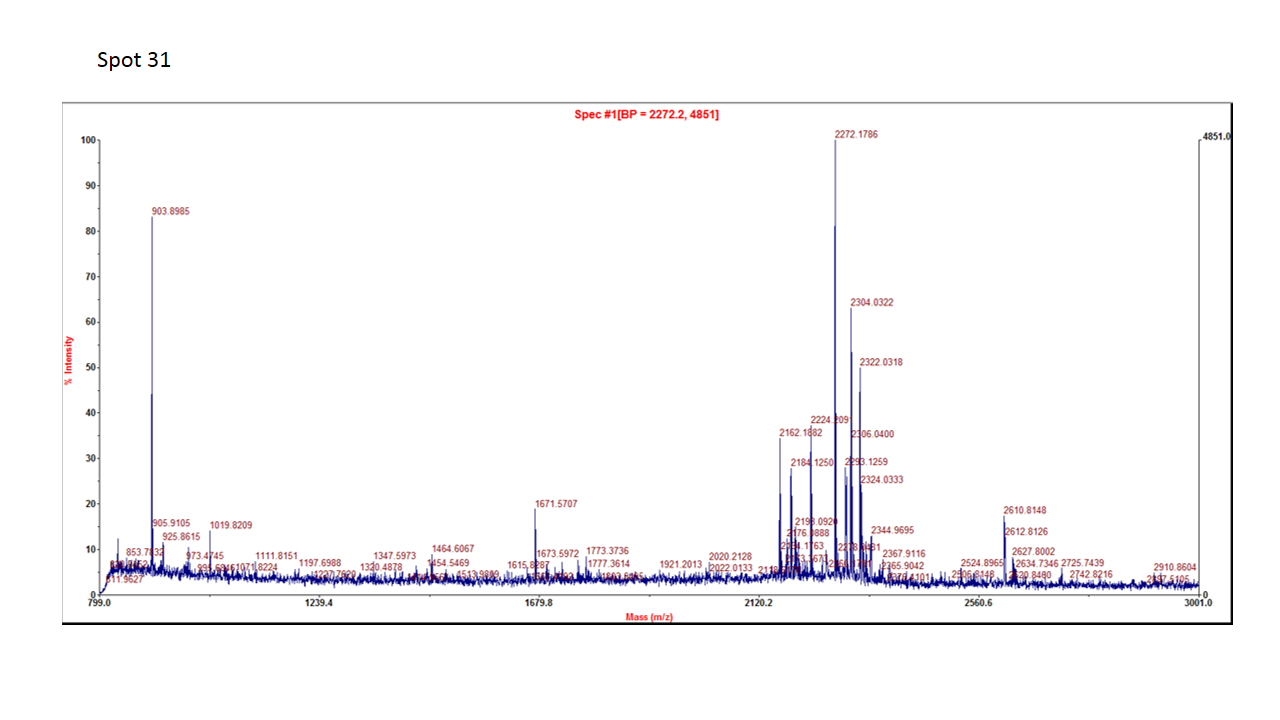

Supplement: S2 File — (ZIP) [file pone.0157439.s007.zip › Slide31.TIF]

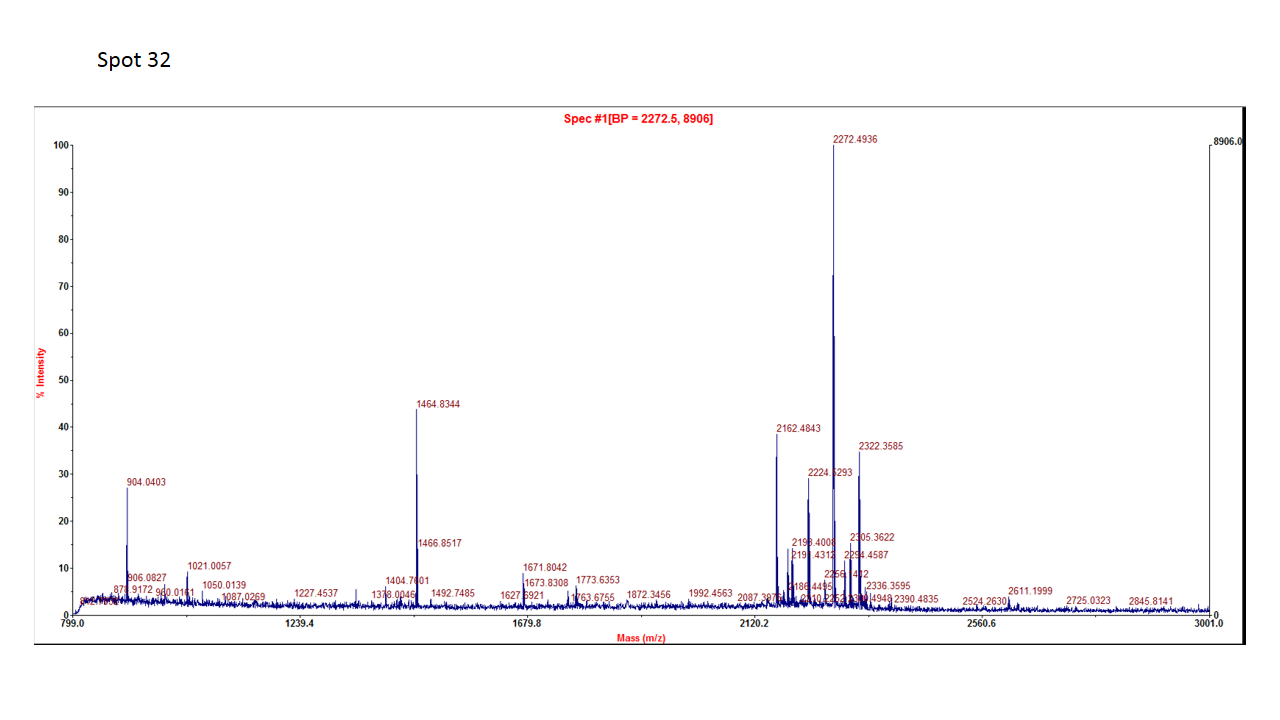

Supplement: S2 File — (ZIP) [file pone.0157439.s007.zip › Slide32.TIF]

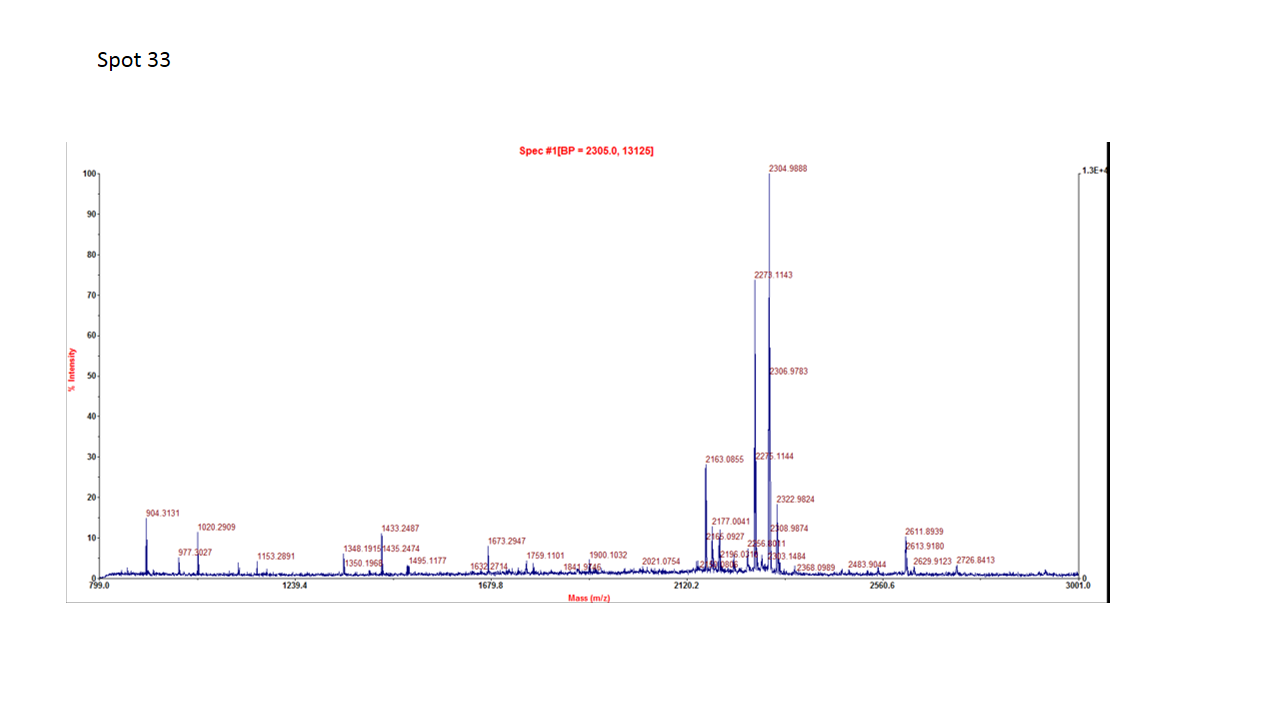

Supplement: S2 File — (ZIP) [file pone.0157439.s007.zip › Slide33.TIF]

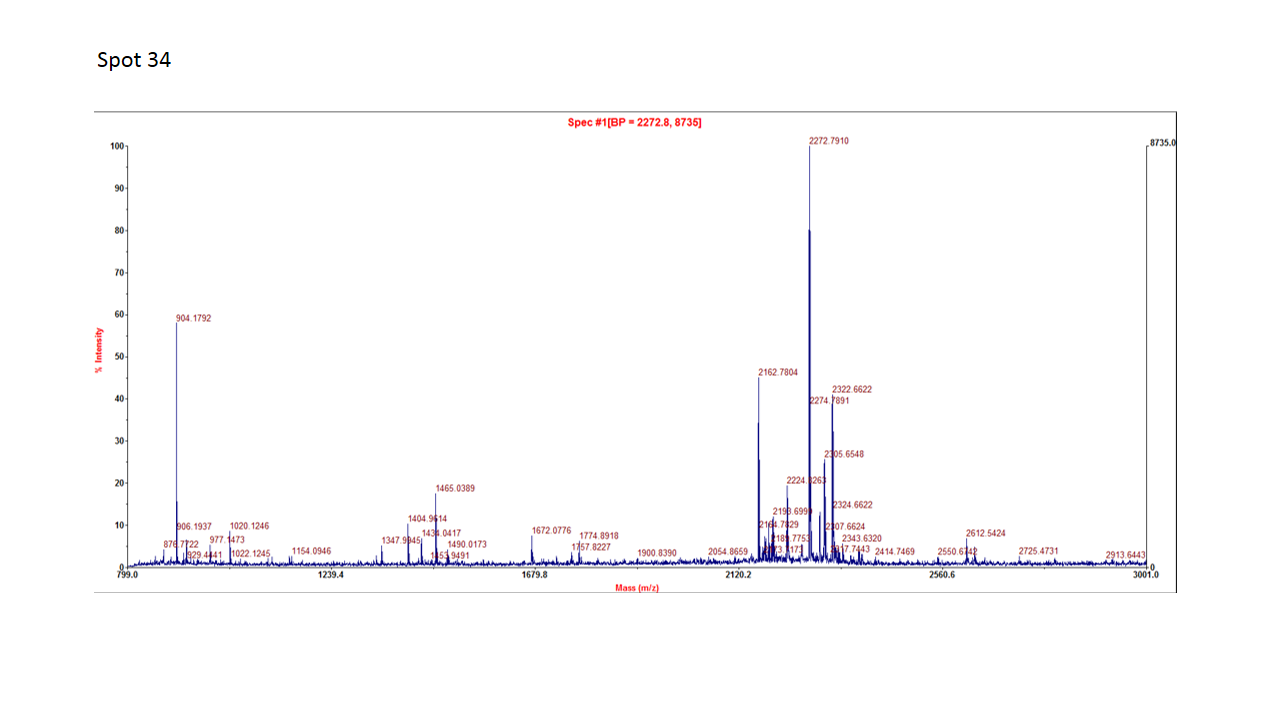

Supplement: S2 File — (ZIP) [file pone.0157439.s007.zip › Slide34.TIF]

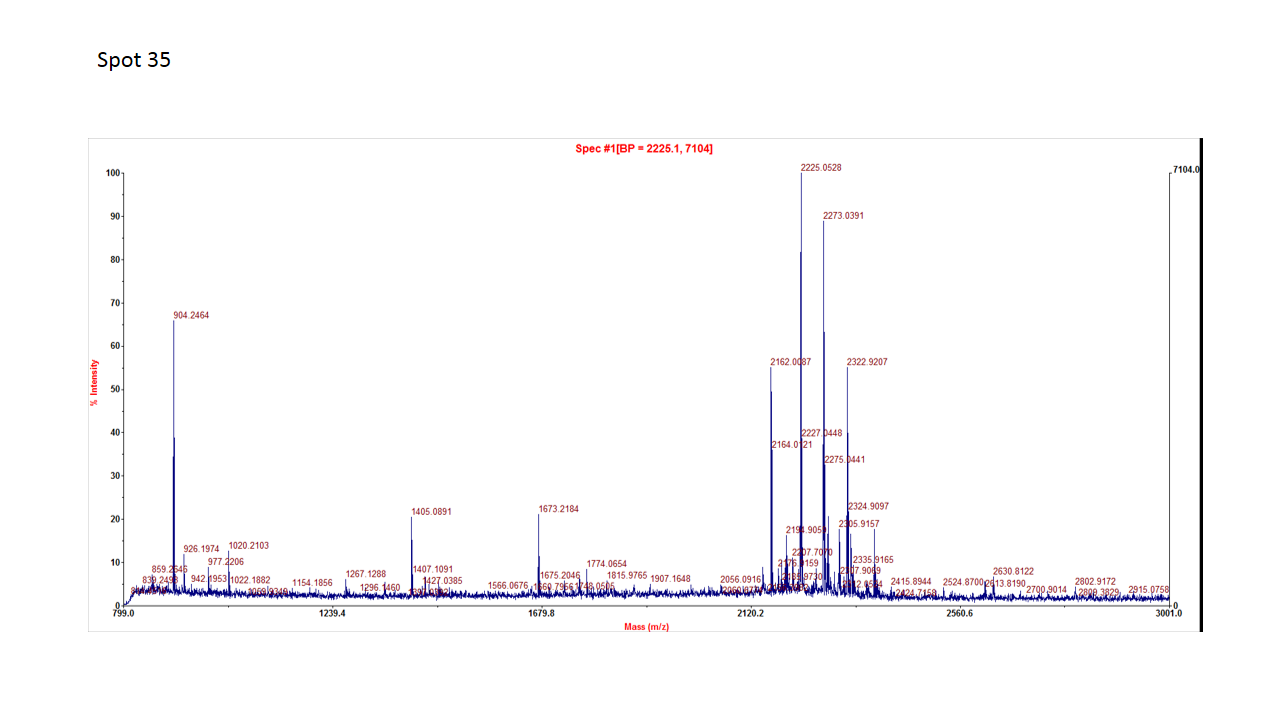

Supplement: S2 File — (ZIP) [file pone.0157439.s007.zip › Slide35.TIF]

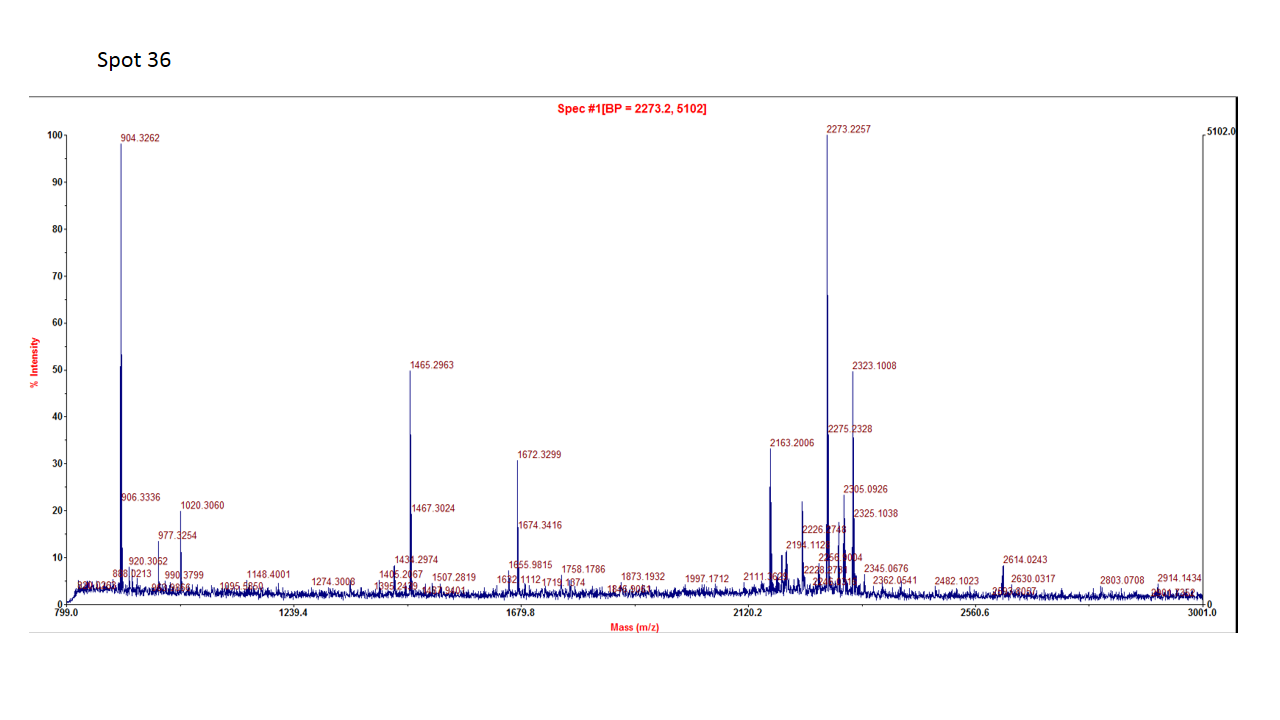

Supplement: S2 File — (ZIP) [file pone.0157439.s007.zip › Slide36.TIF]

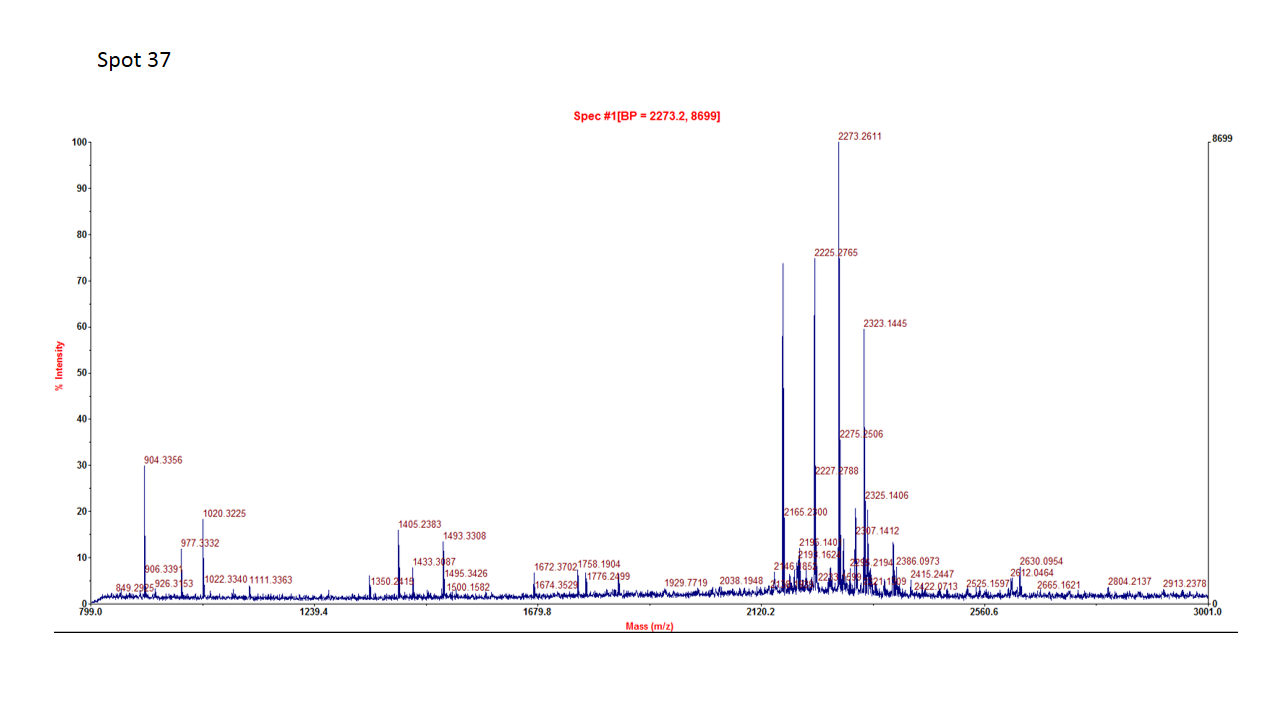

Supplement: S2 File — (ZIP) [file pone.0157439.s007.zip › Slide37.TIF]

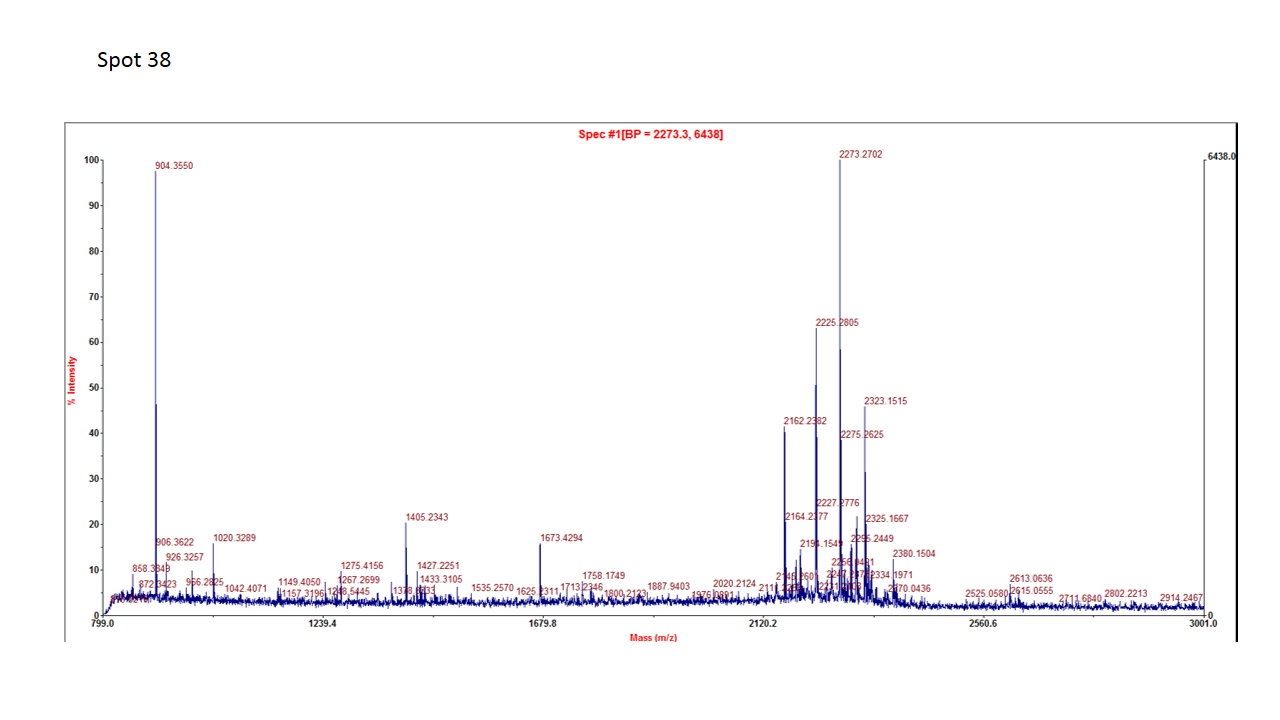

Supplement: S2 File — (ZIP) [file pone.0157439.s007.zip › Slide38.TIF]

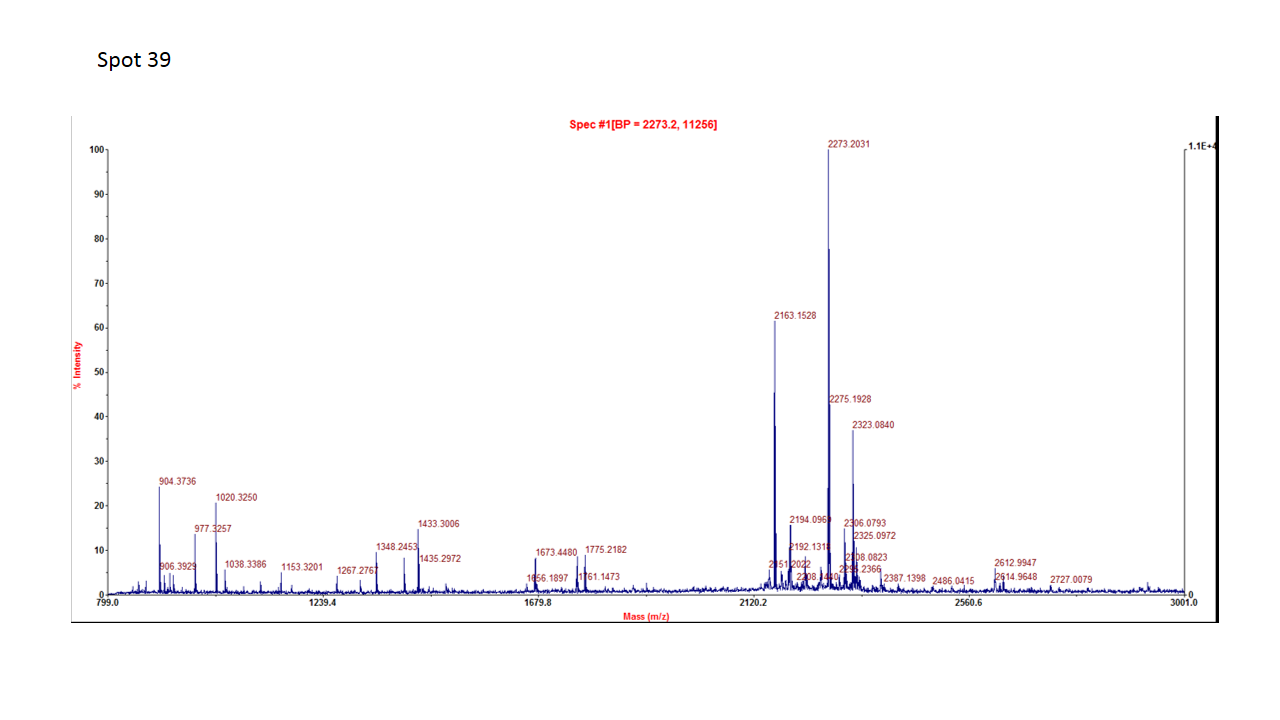

Supplement: S2 File — (ZIP) [file pone.0157439.s007.zip › Slide39.TIF]

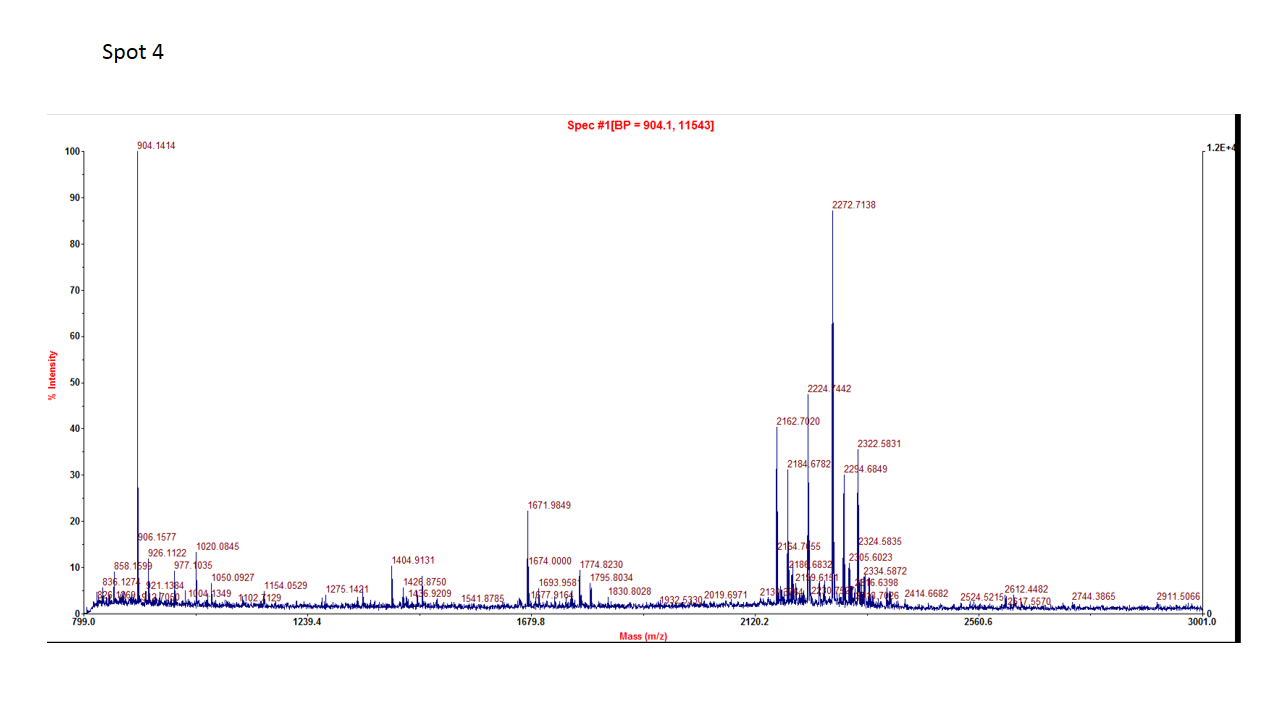

Supplement: S2 File — (ZIP) [file pone.0157439.s007.zip › Slide4.TIF]

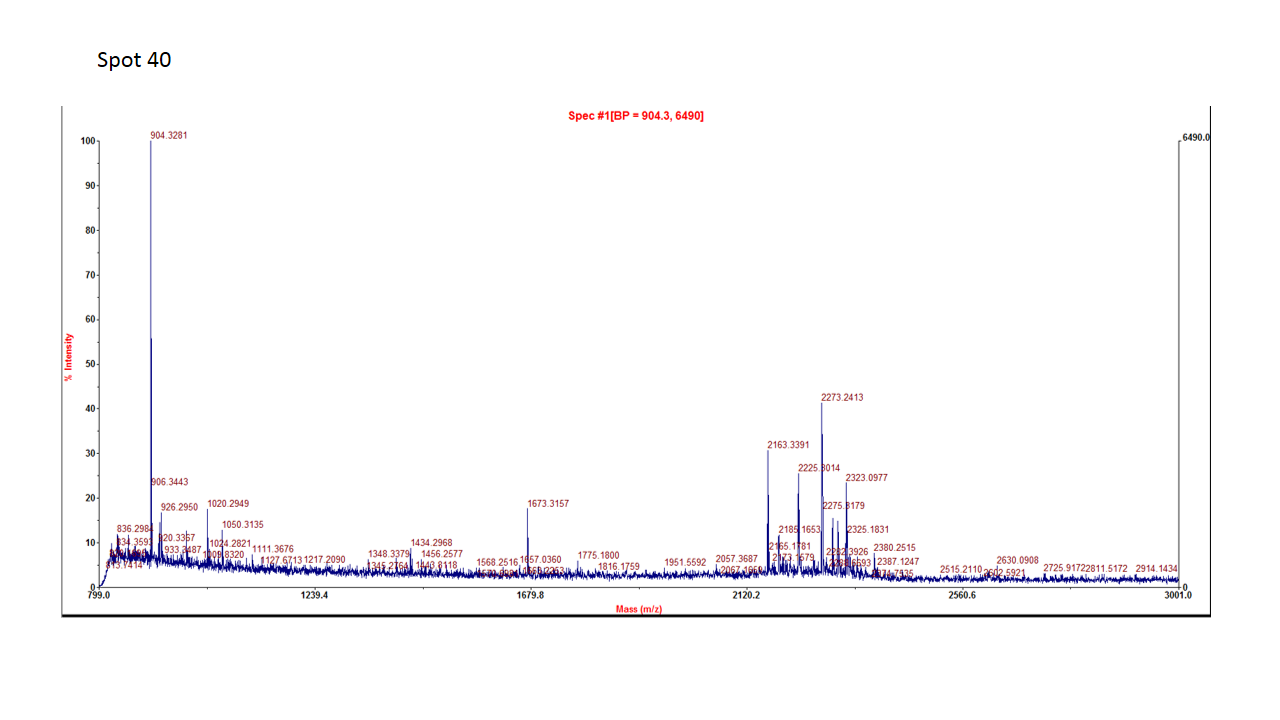

Supplement: S2 File — (ZIP) [file pone.0157439.s007.zip › Slide40.TIF]

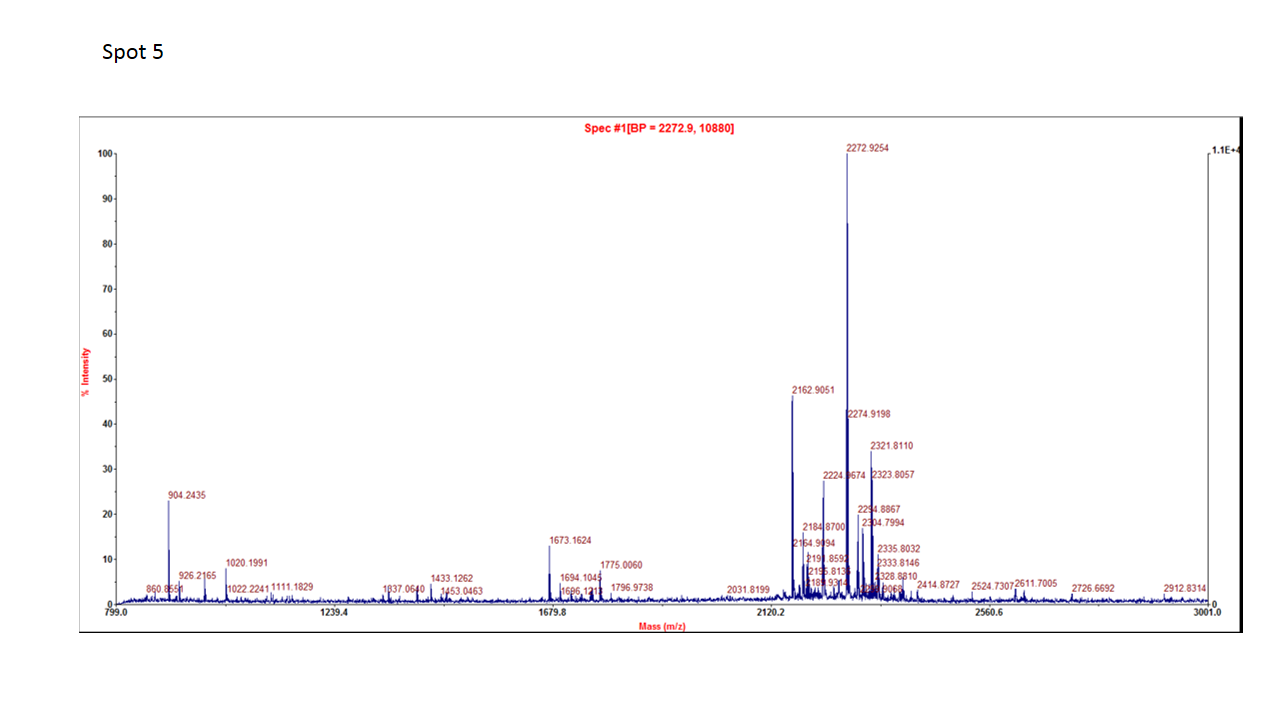

Supplement: S2 File — (ZIP) [file pone.0157439.s007.zip › Slide5.TIF]

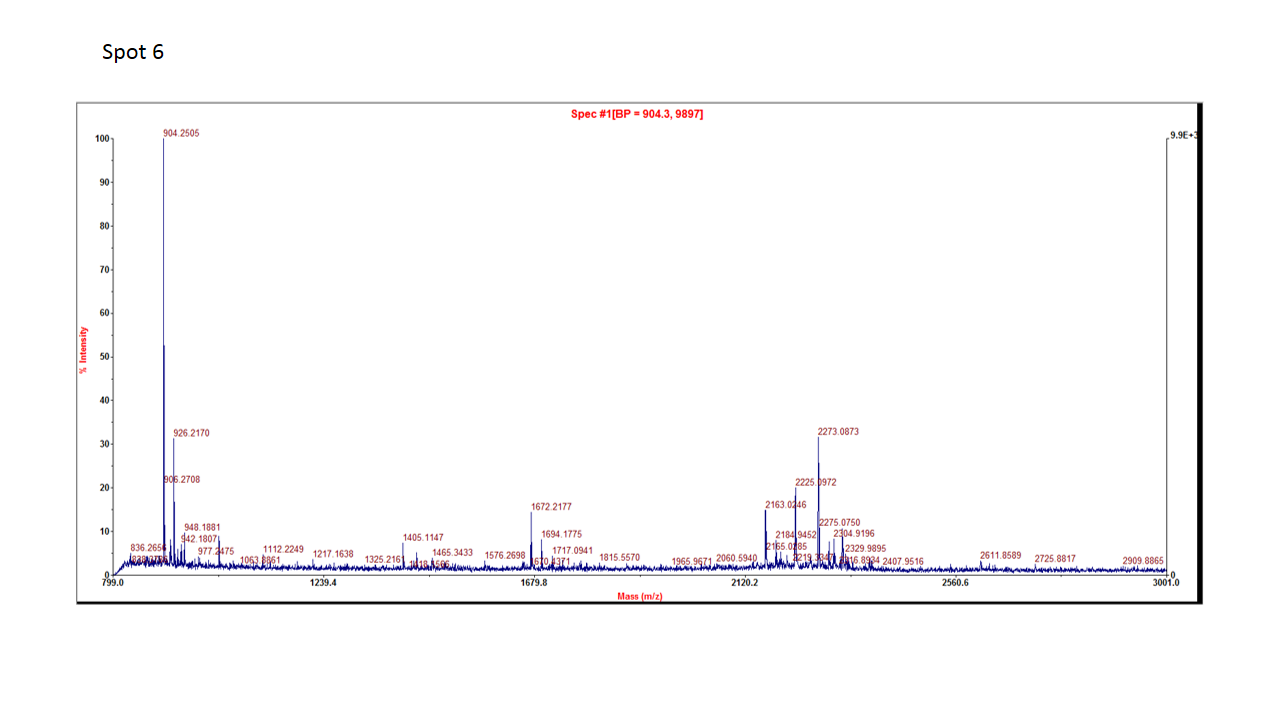

Supplement: S2 File — (ZIP) [file pone.0157439.s007.zip › Slide6.TIF]

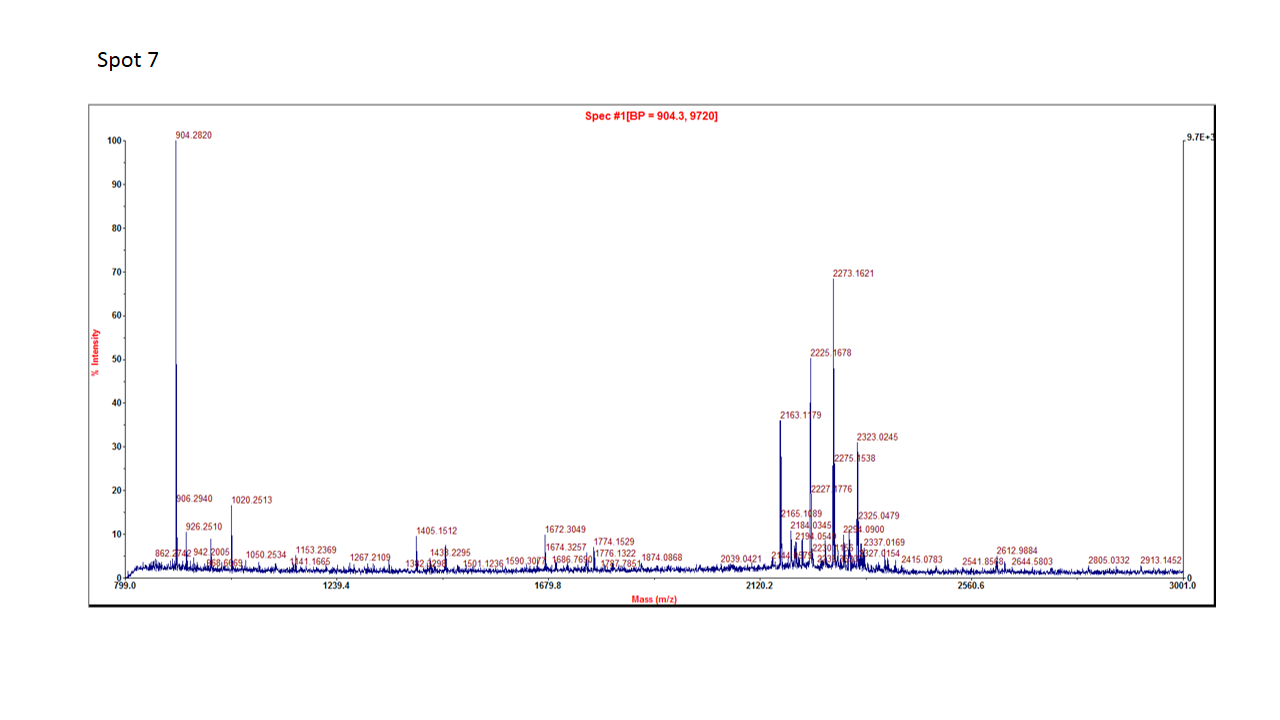

Supplement: S2 File — (ZIP) [file pone.0157439.s007.zip › Slide7.TIF]

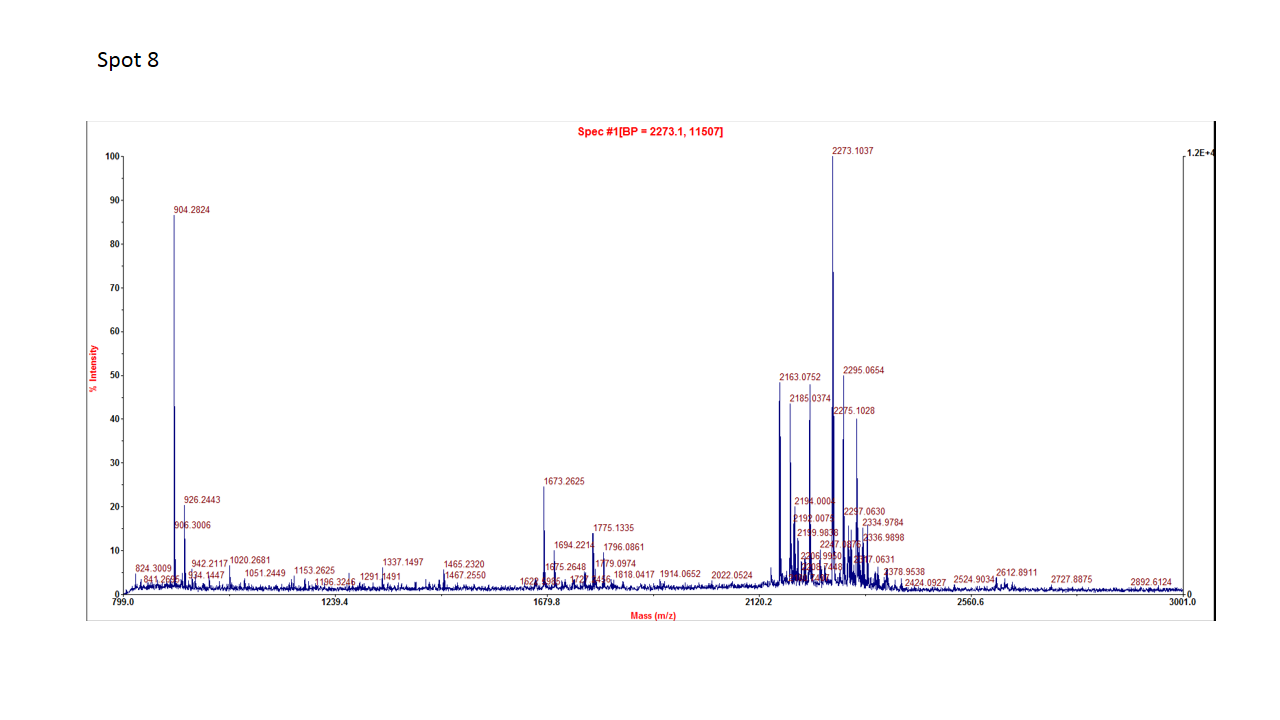

Supplement: S2 File — (ZIP) [file pone.0157439.s007.zip › Slide8.TIF]

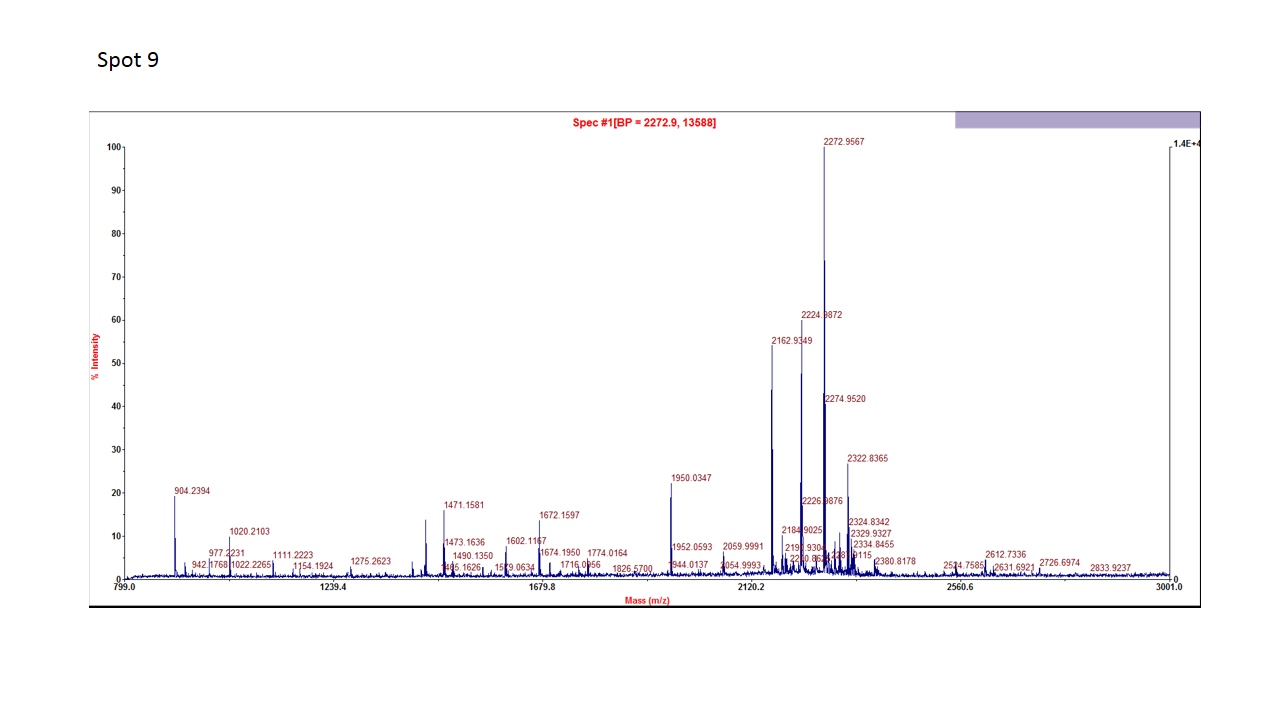

Supplement: S2 File — (ZIP) [file pone.0157439.s007.zip › Slide9.TIF]
